# Boron Lewis Acid Catalysis Enables the Direct Cyanation of Benzyl Alcohols by Means of Isonitrile as Cyanide Source

**DOI:** 10.3390/molecules28052174

**Published:** 2023-02-26

**Authors:** Tong-Tong Xu, Jin-Lan Zhou, Guang-Yuan Cong, Jiang-Yi-Hui Sheng, Shi-Qi Wang, Yating Ma, Jian-Jun Feng

**Affiliations:** State Key Laboratory of Chemo/Biosensing and Chemometrics, Advanced Catalytic Engineering Research Center of the Ministry of Education, College of Chemistry and Chemical Engineering, Hunan University, Changsha 410082, China

**Keywords:** boron Lewis acid, α-aryl nitrile, cyanation, isonitrile, green chemistry

## Abstract

The development of an efficient and straightforward method for cyanation of alcohols is of great value. However, the cyanation of alcohols always requires toxic cyanide sources. Herein, an unprecedented synthetic application of an isonitrile as a safer cyanide source in B(C_6_F_5_)_3_-catalyzed direct cyanation of alcohols is reported. With this approach, a wide range of valuable α-aryl nitriles was synthesized in good to excellent yields (up to 98%). The reaction can be scaled up and the practicability of this approach is further manifested in the synthesis of an anti-inflammatory drug, naproxen. Moreover, experimental studies were performed to illustrate the reaction mechanism.

## 1. Introduction

**Figure 1:**
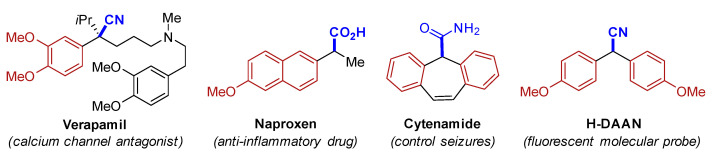
Verapamil, H-DAAN, and some derivatives of α-aryl nitriles.

The need for the development of new reactions that are based on applying the atom-economy concept [1] and avoiding the use of toxic reagents has become a consensus. Alcohols are highly attractive starting materials for synthesis because they are stable, have low toxicity, and are available. Direct nucleophilic substitution of an alcohol is attractive since water is, in principle, the only by-product [2,3,4,5]. However, this reaction is difficult because hydroxide is such a poor leaving group and therefore alcohols are classically derivatized to halides or pseudohalides prior to substitution, which results in the formation of vast amounts of waste. Thus, the development of new catalytic methodologies for dehydrative substitutions of alcohols was considered a central issue, as demonstrated by the inclusion of the “direct substitution of alcohols” in the ACS Green Chemistry Institute^®^ Pharmaceutical Roundtable’s 2018 update on key green chemistry research areas [6]. In this context, the deoxygenative cyanation of readily available benzyl alcohols represent one of the most powerful methods for preparing α-aryl nitriles [7,8,9,10,11,12,13,14,15,16,17,18,19,20], an important class of core structures found in bioactive moleculars [21] and functional materials [22], and precursors that have applications in the synthesis of well-known drugs such as verapamil [23], naproxen [24], and cytenamide [25] as shown in Figure 1.

As early as 1967, the one-pot method for the conversion of alcohols into cyanides based on the concept of the Mitsunobu reaction using NaCN as the cyanide source has been described [26]. Subsequently, there are a few reports on one-pot transformations of alcohols to α-aryl nitrile using Me_3_SiCl/NaI/NaCN [27], PPh_3_/*n*Bu_4_NCN/DDQ [28], *N*-(*p*-toluenesulfonyl)imidazole (TsIm)/NaCN [29], and PPh_3_/DEAD/acetone cyanohydrin [30].

However, these methods suffer from major disadvantages such as the presence of hazardous and toxic cyanide sources and the use of stoichiometric activating reagents.

**Scheme 1:**
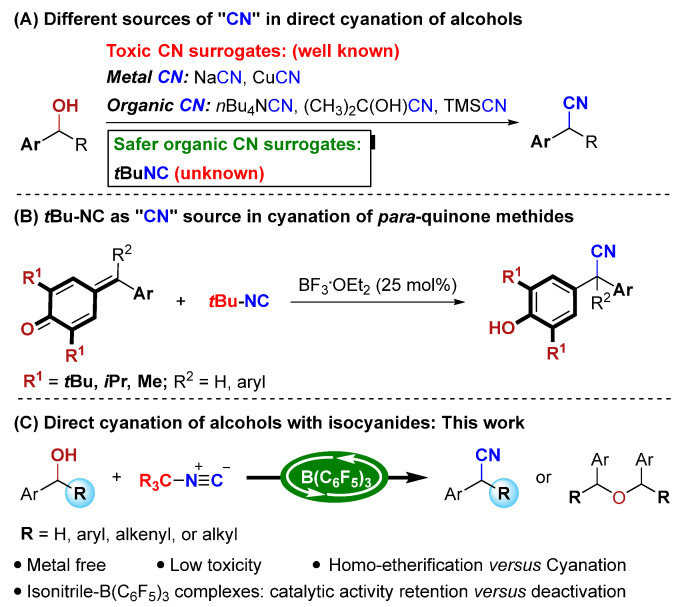
Our catalytic strategy to access α-aryl nitriles and its scientific context.

Recently, catalytic synthesis of α-aryl nitrile from benzyl alcohols was successfully developed. Ding’s group performed pioneering work on the direct cyanation of α-aryl alcohols with trimethylsilyl cyanide (TMSCN) by indium halide catalysis [31]. Later, other Lewis acids, such as FeCl_3_·6H_2_O [32], Zn(OTf)_2_ [33], Brønsted acid montmorillonite catalysts [34], and others [35] were also used to catalyze this transformation. Besides these, ruthenium-catalyzed cyanation of benzyl alcohol with cuprous cyanide (CuCN) was also reported [36]. Again, these methods are typically plagued by notorious toxic cyanide source issues. To solve the severe safety issues associated with the handling of traditional cyanide sources, such as metal cyanides, ketone cyanohydrins [37,38,39], or TMSCN, several safer alternatives have been introduced, including: DMF [40], DMSO/ammonium ion [41], azobisisobutyronitrile (AIBN) [42], TsN(Ph)CN [43], isocyanides [44,45,46,47,48,49,50,51], and so on [52,53]. However, no direct cyanation of alcohols has been described so far for the synthesis of α-aryl nitriles from these safer organic CN surrogates (Scheme 1A).

Isocyanides, which are isoelectronic with carbon monoxide, have emerged as powerful C1 building blocks in organic synthesis [54,55,56,57,58,59,60]. The distinctive reactivity of isocyanides makes them well-known in Passerini and Ugi multicomponent reactions and others [61]. In 1982, Saegusa and co-workers performed seminal work on the conjugate hydrocyanation and 1,2-addition reactions with *tert*-butyl isocyanide in the presence of stoichiometric Lewis acids [44]. Subsequently, commercially available *tert*-butyl isocyanide as a safer cyanide alternative in C-H cyanation [45,46,47,48], noble-metal-catalyzed cyanation of aryl iodides [49], and cyanothiolation of alkynes [50] has been reported, which further broadened the application of isocyanides in organic synthesis. To date, the catalytic method to obtain valuable α-aryl nitriles relied upon the usage of isocyanides as a cyanide source; however, these are rarely known. As a rare example, Muthukrishnan and co-workers reported a BF_3_·OEt_2_-catalyzed 1,6-conjugate addition reaction of *p*-quinone methides (*p*-QMs) with *tert*-butyl isocyanide for synthesis of α-diaryl and α-triaryl nitriles (Scheme 1B) [51]. Nonetheless, the substrates of the reaction are limited to *p*-QMs that feature bulky *tert*-butyl substituents at the 2- and 6-positions. Therefore, the development of new reactions with isocyanides for synthesis of α-aryl nitriles is highly desirable.

In recent years, B(C_6_F_5_)_3_ as a non-metallic Lewis acid has received widespread attention because of its strong Lewis acidity, commercial availability, and environmental friendliness [62,63,64,65,66,67,68]. Although still limited in its success, it mainly involves the B(C_6_F_5_)_3_-catalyzed activation of hydroxyl groups, as reported by Meng, Zhao and Chan [69,70], Marek [71], Tang [72], Maji [73], Gevorgyan [74], and Moran [75]. Inspired by these reports and building on our ongoing interest in the developing atom-economic reactions [76,77,78,79], we questioned if the direct cyanation of alcohols with isocyanides in the presence of B(C_6_F_5_)_3_ could be realized to meet the requirements of atom economy and green chemistry (Scheme 1C). However, this hypothesis may face considerable challenges, such as the following: (a) the catalyst should be stable in wet and Lewis basic conditions, and (b) *tert*-butylisocyanide/B(C_6_F_5_)_3_ and nitrile/B(C_6_F_5_)_3_ adducts can be easily formed, as reported by Berke and Erker and co-workers [80]. It is unknown whether B(C_6_F_5_)_3_ can maintain its catalytic activity during the current cyanation reaction, and (c) the catalyst should be able to dissociate from nitrile products. Finally, (d) another challenge is to suppress the B(C_6_F_5_)_3_-catalyzed homo-etherification of alcohols reported by Chan and co-workers [70].

## 2. Results

We initiated our investigation with the optimization of the reaction of benzhydryl alcohol (**1a**) and *tert*-butyl isocyanide (**2a**). Initially, **1a** and 1.5 equivalents of **2a** were subjected to a solution of FeCl_3_ (10 mol%) in toluene at 100 °C (Table 1, entry 1). However, FeCl_3_ showed almost no catalytic activity. Other commonly used Lewis acids, including AlCl_3_, Cu(OTf)_2_, AgClO_4_, and BF_3_·(OEt)_2_ were also tested, but led to low yield (see entries 2–5 in Table 1). Of note, using AlCl_3_, BF_3_·(OEt)_2_, or TsOH·H_2_O as a catalyst, a mixture of **3a** and some homo-etherification side product **4a** was obtained (entries 2, 5 and 7). With the use of Brønsted acid Tf_2_NH, the cyanation provided the desired **3a** in 72% NMR yield. Interestingly, the reaction favors the formation of etherification product **4a** rather than **3a** when using diphenyl phosphate as the catalyst (entry 8 versus 9). Gratifyingly, we found that B(C_6_F_5_)_3_ affords the desired α-aryl nitrile **3a** in >99% NMR yield at 100 °C (entry 6). No reaction occurred when B(C_6_F_5_)_3_ was used as the catalyst at 50 °C (entry 11). Increasing the temperature to 80 °C improved both the yield of **3a** (20%) and ether **4a** (30%). Different solvents such as 1,2-dichloroethane (DCE), THF, and hexafluoroisopropanol (HFIP) (entries 12–15) were also tested, and the results revealed that toluene was superior to other solvents.

**Scheme 2:**
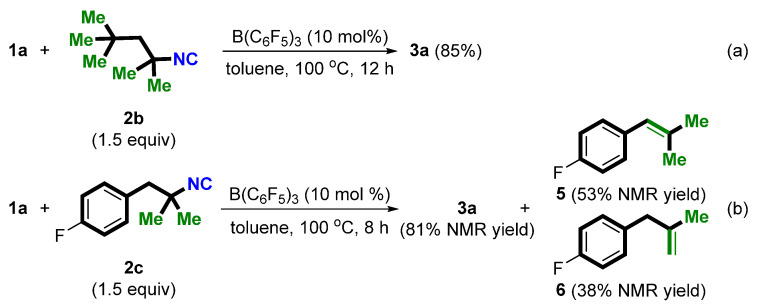
Survey of the scope of isocyanides (**a**,**b**).

With the optimized conditions identified, we then proceeded to explore the scope of isocyanides (Scheme 2). Besides *t*Bu-NC (**2a**), other tertiary amine-derived isocyanides, such as **2b** and **2c**, can also be used as a novel cyano source in the current direct cyanation of α-aryl alcohols. In contrast, when secondary amine- and aniline-derived isocyanides were used as substrates, only the corresponding ether products were obtained (not shown). Of note, treatment of **1a** and **2c** with the standard conditions afforded **3a** in 81% NMR yield together with internal alkene **5** in 53% NMR yield and terminal alkene **6** in 38% NMR yield (Scheme 2b), indicating a tertiary carbon cation might be an intermediate.

**Scheme 3:**
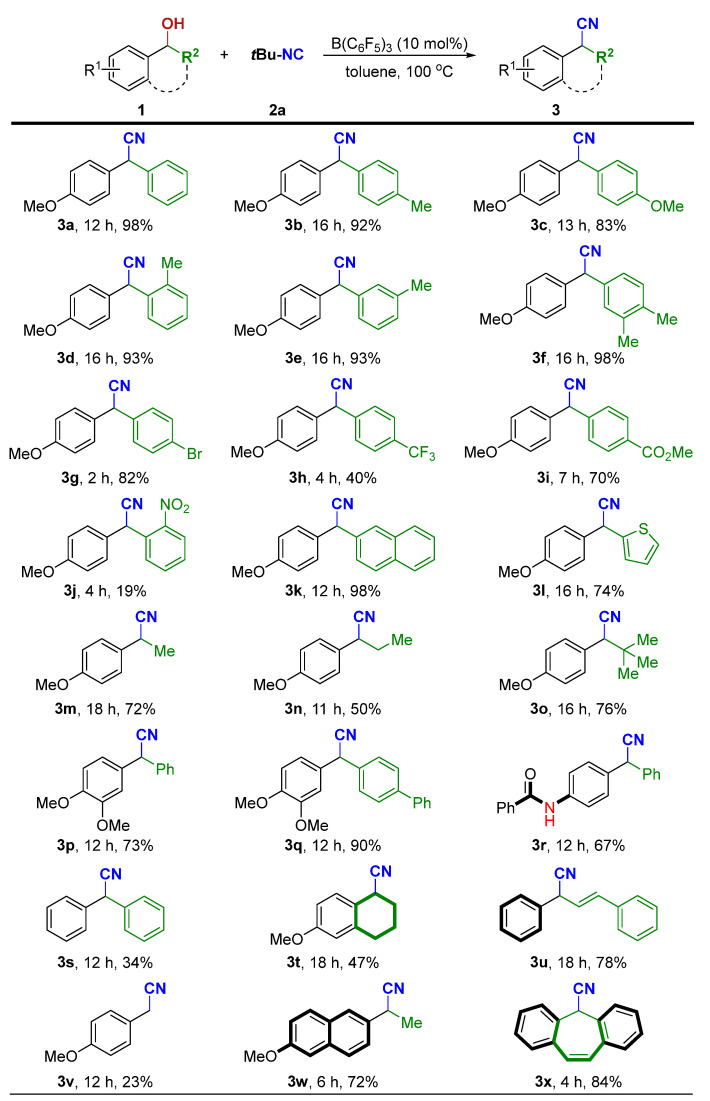
Survey of the scope of α-aryl alcohols ^[a,b]^. ^[a]^ Standard conditions: **1** (0.20 mmol), **2a** (0.30 mmol, 1.5 equiv), B(C_6_F_5_)_3_ (10 mol%), toluene (2 mL), at 100 °C for 2–18 h. ^[b]^ Isolated yield of **3**.

Next, we turned to explore the generality of this cyanidation reaction with a variety of alcohols with *t*Bu-NC (**2a**). As shown in Scheme 3, a wide range of benzylic alcohols can smoothly react with **2a** under the optimized conditions, giving the corresponding α-aryl nitriles in good to excellent yields. Diarylsubstituted alcohols (R^2^ = aryl) underwent reaction with *t*Bu-NC to furnish the corresponding products (**3a**–**3k**) in 19−98% yields.

Diarylsubstituted alcohols bearing electron-donating (methoxy, alkyl) groups at the *para*-, *ortho*- or *meta*-positions of the benzene rings reacted smoothly (**1a**–**1f**). A variety of electron-withdrawing functional groups at the *para*-positions of the benzene rings such as -Br, -CF_3_, and -CO_2_Me were tolerated, affording the desired products in moderate to good yields (**1g**–**1i**). However, a low yield of **3j** was isolated from **1j** having an *ortho*-nitro group on the aromatic substituent. The naphthyl-containing alcohol **1k** and heteroaryl-containing alcohol **1l** also afforded the corresponding products in good to excellent yields. In addition, alkyl-substituted alcohols (R^2^ = methyl, ethyl, and *tert*-butyl) were also well-tolerated to afford the desired products **3m**–**3o**. Of note, a competitive side reaction encountered in the reaction with **1m** or **1n** is the formation of styrene derivatives via the dehydration reactions of alcohols. Besides R^1^ = methoxy (**1p**–**1q**), the substituent R^1^ can be benzamide (**1r**). Benzhydrol **1s** was tested but afforded the corresponding product **3s** in low yield together with (oxybis(methanetriyl))tetrabenzene in 64% NMR yield. However, 1,2,3,4-tetrahydronaphthalen-1-ol (**1t**) and allylic alcohol (**1u**) underwent smooth cyanation. It is worth noting that the reported indium halide-catalyzed protocol with TMSCN as the cyanide source is not amenable to primary alcohol **1v** for cyanation reaction [31]. Our system, however, gives reasonable yield for the same substrate. To our delight, the precursors for naproxen and cytenamide, respectively, can also be efficiently obtained in high yields by this protocol (**3w** and **3x**).

**Scheme 4:**
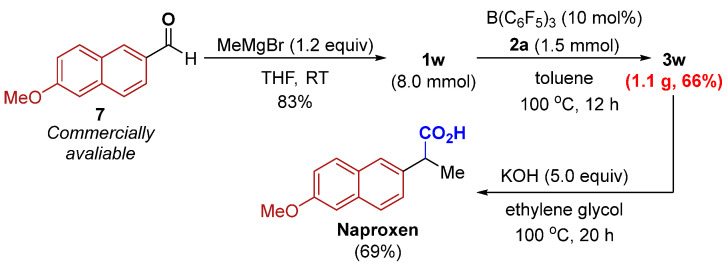
Scale-up synthesis and synthetic transformations.

To disclose the synthetic practicability of the developed method, we studied a gram-scale reaction, and 66% yield of **3w** was obtained, which might provide potential value in synthetic chemistry. Having established a protocol for the efficient synthesis of α-aryl nitrile **3w**, (±)-naproxen, a nonsteroidal anti-inflammatory drug [24], was prepared in three steps from commercially available materials (Scheme 4).

**Scheme 5:**
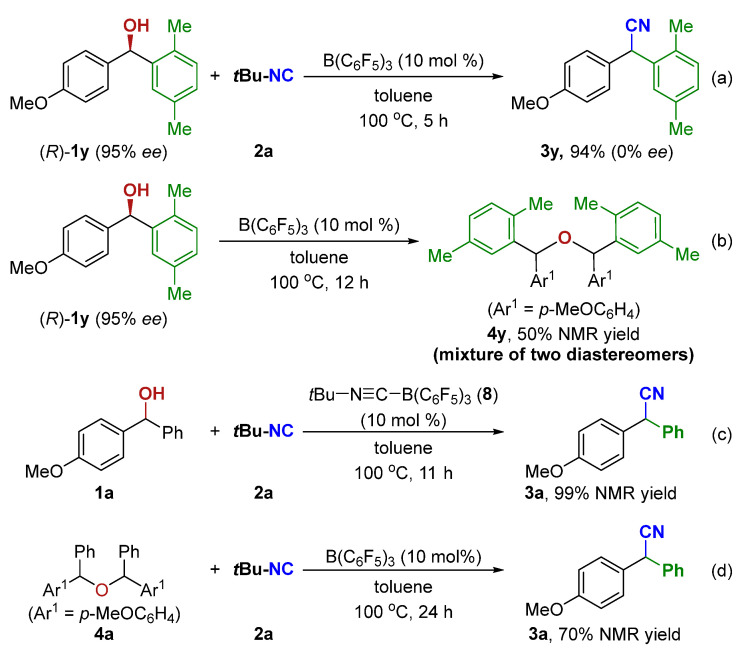
Control experiments (**a**–**d**).

To gain insight into the reaction mechanism, several control experiments were conducted. When an enantiomerically pure sample of (*R*)-**1y** was subjected to standard conditions, the resulting nitrile **3y** was obtained in racemic form (Scheme 5a). Moreover, (*R*)-**1y** was also employed to perform the etherification reaction under the standard condition. The ^1^H NMR spectrum showed the ether **4y** was obtained in 50% NMR yield with a 48/52 ratio of the two diastereomers (Scheme 5b). These control experiments support an S_N_1 pathway and rule out a concerted S_N_2 mechanism. The *tert*-Butylisocyanide-B(C_6_F_5_)_3_ adduct (**8**) was easily prepared [80] and used as a catalyst in the current reaction, affording the corresponding product **3a** in 99% NMR yield (Scheme 5c). As shown in Table 1, entry 10, the reaction did form **3a** in 20% NMR yield along with ether **4a** in 30% NMR yield at 80 °C. Treatment of **4a** with the standard setup then gave the desired **3a** in 70% NMR yield (Scheme 5d).

**Scheme 6:**
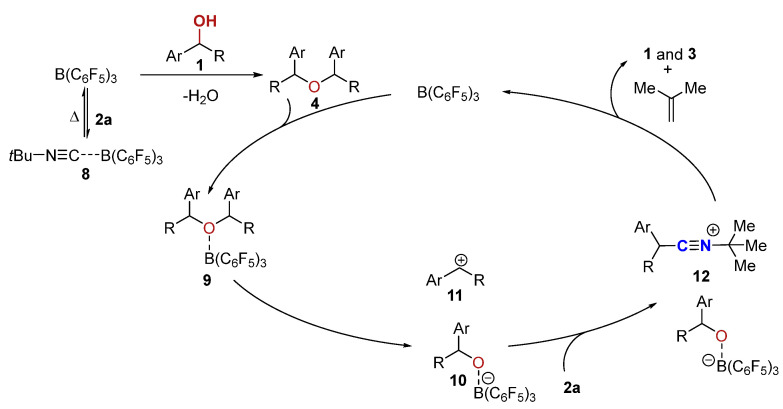
Proposed mechanism.

Based on the results above, a plausible mechanism is proposed in Scheme 6. First, *tert*-butylisocyanide **2a** forms a reversibly Lewis adduct **8** with B(C_6_F_5_)_3_ [80]. The homo-etherification of alcohol in the presence of the B(C_6_F_5_)_3_ quickly delivers the ether **4** [70], which could furnish an adduct **9** with B(C_6_F_5_)_3_ through the oxygen center. Subsequently, the adduct **9** could break into an intermediate **10** and carbocation **11**. However, an alternative reaction path for the formation of the carbocation **11** directly from alcohol in the presence of the in situ-generated strong Brønsted acid B(C_6_F_5_)_3_·nH_2_O or boron Lewis acid B(C_6_F_5_)_3_ (not shown, see Supporting Information for details) cannot be ruled out [69,70]. The carbocation **11** could then be intercepted instantaneously by the *t*Bu-NC (**2a**) to afford an intermediate **12** with a borate anion **10** as the counteranion. The stability of the tertiary carbon cation is the driving force to break the C-N bond in **12**, leading to the α-aryl nitrile **3** and 2-methylpropene by proton elimination via a *tert*-butyl carbon cation intermediate (supported by Scheme 2b) [81]. The borate anion **10**, on the other hand, transforms into alcohol **1** with the regeneration of the B(C_6_F_5_)_3_ catalyst.

## 3. Materials and Methods

### 3.1. General Information

All reactions were performed in flame-dried glassware using conventional Schlenk techniques under a static pressure of nitrogen unless otherwise stated. Liquids and solutions were transferred with syringes. The known alcohols **1** [31,35] and *t*Bu-NC-B(C_5_F_5_)_3_ adduct **8** [80] were prepared according to reported procedures. (*R*)-**1y** was prepared in 80% yield according to the known procedure [82] (95% *ee* of (*R*)-**1y** was determined by HPLC: OJ-H Column, 5/95 iPrOH/hexane, 0.5 mL/min, 254 nm, 35 °C; retention time = 75.36 min (minor), 81.66 min (major)). Tris(pentafluorophenyl)borane (B(C_5_F_5_)_3_, 98%, *Energy Chemical*) and *tert*-butyl isocyanate (97%, *Energy Chemical*) were purchased from commercial suppliers and used as received. Other commercially available reagents were purchased from *Sigma-Adrich*, *Leyan* and *Bide* Chemical Company. All solvents (tetrahydrofuran, toluene, and 1,2-dichloroethane etc.) were dried and purified following standard procedures. Technical grade solvents for extraction or chromatography (petroleum ether, CH_2_Cl_2_, and ethyl acetate) were distilled prior to use. Analytical thin layer chromatography (TLC) was performed on silica gel 60 F254 glass plates by *Merck*. Flash column chromatography was performed on silica gel 60 (40–63 μm, 230–400 mesh, ASTM) by *Grace* using the indicated solvents. ^1^H, ^13^C NMR spectra (Supplementary Materials) were recorded in CDCl_3_ on Bruker AV400 instruments. Chemical shifts are reported in parts per million (ppm) and are referenced to the residual solvent resonance as the internal standard (CHCl_3_: δ = 7.26 ppm for ^1^H NMR and CDCl_3_: δ = 77.0 ppm for ^13^C NMR). Data are reported as follows: chemical shift, multiplicity (s = singlet, d = doublet, t = triplett, q = quartet, m = multiplet), coupling constants (Hz), and integration. Mass spectra were recorded on a THERMO FINNIGAN LTQ-XL. The MS inlet capillary temp was always maintained at 275 °C and capillary voltage at 5 kV. No other source gases were used when digestion was performed in microdroplets. The samples were dissolved in 1:1 methanol:water.

### 3.2. Typical Procedure for Direct Cyanation of Alcohols

In a glove box, alcohol **1** (0.2 mmol), isocyanide **2** (0.3 mmol, 1.5 equiv), B(C_6_F_5_)_3_ (10.2 mg, 20 μmol, 10 mol%), and toluene (2.0 mL) were added to an oven-dried 10 mL pressure vial. The vial was sealed and removed from the glove box. The reaction mixture was stirred at 100 °C (oil bath) for 2–18 h. After the reaction was completed, the reaction mixture was purified by silica gel column chromatography by using petroleum ether/ethyl acetate mixture to obtain the desired nitrile **3**.

### 3.3. Procedure for the Preparation of Naproxen

To a solution of 6-methoxy-2-naphthaldehyde (1.86 g, 10.0 mmol, 1.0 equiv) in THF (20 mL, 0.5 M), methylmagnesium bromide (4.0 mL, 12 mmol, 3.0 M, 1.2 equiv) was added. When the reaction was judged to have reached completion (as determined by TLC), sat. NH_4_Cl was added slowly at 0 °C, and the mixture was extracted with EtOAc. The combined organic layers were washed with brine, dried over MgSO_4_, and purified by column chromatography on silica gel to obtain **1w** (1.60 g, 80% yield).

To an oven-dried 200 mL Schlenk flask containing a magnetic stir bar under an atmosphere of nitrogen, alcohol **1w** (1.62 g, 8.0 mmol), isocyanide **2a** (1.0 g, 12 mmol, 1.5 equiv), B(C_6_F_5_)_3_ (0.41 g, 0.8 mmol, 10 mol%), and toluene (80 mL) were added. The reaction mixture was stirred at 100 °C (oil bath) for 12 h. After the reaction was completed, the reaction mixture was purified by silica gel column chromatography by using petroleum ether/ethyl acetate mixture to obtain the desired nitrile **3w** (1.12 g, 66% yield).

To a suspension of **3w** (42.3 mg, 0.2 mmol, 1.0 equiv) in ethylene glycol (0.6 mL), KOH (0.1 mL, 10 M solution in H_2_O, 5.0 equiv) was added. The reaction vessel was sealed with a rubber septum and submerged in an oil bath at 100 °C for 20 h. We then added 1M HCl (2.5 mL) dropwise, and the mixture was extracted with EtOAc. The combined organic layers were washed with brine, dried over MgSO_4_, and concentrated under reduced pressure. The residue was purified by column chromatography on silica gel to obtain Naproxen (31.8 mg, 69% yield).

### 3.4. Characterization Data of the Products

**2-(4-methoxyphenyl)-2-phenylacetonitrile** (**3a**) [83]. White solid (43.8 mg, 98% yield); mp 130–132 °C; R*_f_* = 0.50 (petroleum ether/EtOAc = 20/1). ^1^H NMR (400 MHz, CDCl_3_): δ 7.39–7.29 (m, 5H), 7.25 (d, *J* = 8.4 Hz, 2H), 6.88 (d, *J* = 8.4 Hz, 2H), 5.09 (s, 1H), 3.79 (s, 3H) ppm. ^13^C NMR (100 MHz, CDCl_3_): δ 159.4, 136.2, 129.1, 128.9, 128.1, 127.9, 127.6, 119.9, 114.5, 55.3, 41.8 ppm.

**2-(4-methoxyphenyl)-2-(*p*-tolyl)acetonitrile** (**3b**) [83]. White solid (43.9 mg, 92% yield); mp 85–87 °C; R*_f_* = 0.50 (petroleum ether/EtOAc = 20/1). ^1^H NMR (400 MHz, CDCl_3_): δ 7.25–7.15 (m, 6H), 6.87 (d, *J* = 8.8 Hz, 2H), 5.05 (s, 1H), 3.79 (s, 3H), 2.33 (s, 3H) ppm. ^13^C NMR (100 MHz, CDCl_3_): δ 159.3, 137.9, 133.2, 129.8, 128.8, 128.2, 127.5, 120.0, 114.4, 55.3, 41.4, 21.0 ppm.

**2,2-bis(4-methoxyphenyl)acetonitrile** (**3c**) [83]. White solid (41.9 mg, 83% yield); mp 156–158 °C; R*_f_* = 0.50 (petroleum ether/EtOAc = 20/1). ^1^H NMR (400 MHz, CDCl_3_): δ 7.23 (d, *J* = 8.4 Hz, 4H), 6.88 (d, *J* = 8.8 Hz, 4H), 5.04 (s, 1H), 3.79 (s, 6H) ppm. ^13^C NMR (100 MHz, CDCl_3_): δ 159.3, 128.7, 128.2, 120.1, 114.4, 55.3, 41.0 ppm.

**2-(4-methoxyphenyl)-2-(*o*-tolyl)acetonitrile** (**3d**) [83]. Colorless oil (44.4 mg, 93% yield); R*_f_* = 0.50 (petroleum ether/EtOAc = 20/1). ^1^H NMR (400 MHz, CDCl_3_): δ 7.40–7.38 (m, 1H), 7.25 (t, *J* = 4.8 Hz, 2H), 7.20–7.17 (m, 3H), 6.87 (d, *J* = 8.8 Hz, 2H), 5.23 (s, 1H), 3.78 (s, 3H), 2.26 (s, 3H) ppm. ^13^C NMR (100 MHz, CDCl_3_): δ 159.3, 135.8, 133.8, 131.2, 128.9, 128.5, 128.4, 126.8, 126.7, 119.8, 114.4, 55.3, 39.1, 19.4 ppm. MS (ESI) *m*/*z*: [M+H]^+^ calcd. for C_16_H_16_NO: 238.12; found: 238.18.

**2-(4-methoxyphenyl)-2-(*m*-tolyl)acetonitrile** (**3e**) [83]. Colorless oil (44.4 mg, 93% yield); R*_f_* = 0.50 (petroleum ether/EtOAc = 20/1). ^1^H NMR (400 MHz, CDCl_3_): δ 7.25–7.22 (m, 3H), 7.14–7.10 (m, 3H), 6.88 (d, *J* = 8.4 Hz, 2H), 5.04 (s, 1H), 3.78 (s, 3H), 2.33 (s, 3H) ppm. ^13^C NMR (100 MHz, CDCl_3_): δ 159.3, 139.0, 136.1, 128.9, 128.84, 128.81, 128.2, 128.0, 124.6, 120.0, 114.4, 55.3, 41.7, 21.3 ppm. MS (ESI) *m*/*z*: [M+H]^+^ calcd. for C_16_H_16_NO: 238.12; found: 238.82.

**2-(3,4-dimethylphenyl)-2-(4-methoxyphenyl)acetonitrile** (**3f**) [83]. Colorless oil (55.1 mg, 98% yield); R*_f_* = 0.50 (petroleum ether/EtOAc = 20/1). ^1^H NMR (400 MHz, CDCl_3_): δ 7.24 (d, *J* = 6.8 Hz, 2H), 7.12–7.03 (m, 3H), 6.87 (d, *J* = 6.4 Hz, 2H), 5.02 (s, 1H), 3.78 (s, 3H), 2.24 (s, 6H) ppm. ^13^C NMR (100 MHz, CDCl_3_): δ 159.3, 137.5, 136.6, 133.6, 130.2, 128.8, 128.7, 128.3, 124.9, 120.1, 114.4, 55.3, 41.4, 19.8, 19.4 ppm. MS (ESI) *m*/*z*: [M+H]^+^ calcd. for C_17_H_18_NO: 252.14; found: 252.18.

**2-(4-bromophenyl)-2-(4-methoxyphenyl)acetonitrile** (**3g**) [83]. Colorless oil (49.4 mg, 82% yield); R*_f_* = 0.50 (petroleum ether/EtOAc = 20/1). ^1^H NMR (400 MHz, CDCl_3_): δ 7.48 (d, *J* = 8.4 Hz, 2H), 7.20 (t, *J* = 7.5 Hz, 4H), 6.88 (d, *J* = 8.4 Hz, 2H), 5.04 (s, 1H), 3.79 (s, 3H) ppm. ^13^C NMR (100 MHz, CDCl_3_): δ 159.6, 135.3, 132.2, 129.2, 128.8, 127.2, 122.2, 119.3, 114.6, 55.3, 41.2 ppm. MS (ESI) *m*/*z*: [M+H]^+^ calcd. for C_15_H_13_BrNO: 302.02; found: 302.36.

**2-(4-methoxyphenyl)-2-(4-(trifluoromethyl)phenyl)acetonitrile** (**3h**). Colorless oil (23.2 mg, 40% yield); R*_f_* = 0.40 (petroleum ether/EtOAc = 10/1). ^1^H NMR (400 MHz, CDCl_3_): δ 7.63 (d, *J* = 8.4 Hz, 2H), 7.47 (d, *J* = 8.0 Hz, 2H), 7.24 (d, *J* = 8.4 Hz, 2H), 6.91 (d, *J* = 8.4 Hz, 2H), 5.14 (s, 1H), 3.80 (s, 3H) ppm. ^13^C NMR (100 MHz, CDCl_3_): δ 159.8, 140.1, 130.6 (q, *J* = 32.8 Hz), 128.9, 128.0, 126.9, 126.1 (q, *J* = 3.7 Hz), 123.8 (q, *J* = 270.5 Hz, 1H), 119.1, 114.8, 55.3, 41.6 ppm. ^19^F NMR (376 MHz, CDCl_3_) δ −62.75 (s) ppm. HRMS (MALDI-TOF/TOF) for C_16_H_13_F_3_NO [M+H]^+^: calculated 292.0944, found 292.0947.

**Methyl 4-(cyano(4-methoxyphenyl)methyl)benzoate** (**3i**). Yellow oil (39.4 mg, 70% yield); R*_f_* = 0.50 (petroleum ether/EtOAc = 5/1). ^1^H NMR (400 MHz, CDCl_3_): δ 8.03 (d, *J* = 8.2 Hz, 2H), 7.42 (d, *J* = 8.2 Hz, 2H), 7.23 (d, *J* = 8.6 Hz, 2H), 6.89 (d, *J* = 8.6 Hz, 2H), 5.14 (s, 1H), 3.91 (s, 3H), 3.79 (s, 3H) ppm. ^13^C NMR (100 MHz, CDCl_3_): δ 166.3, 159.6, 141.0, 130.4, 130.1, 128.9, 127.6, 127.1, 119.2, 114.7, 55.3, 52.2, 41.7 ppm. HRMS (ESI) for C_17_H_16_NO_3_ [M+H]^+^: calculated 282.1125, found 282.1126.

**2-(4-methoxyphenyl)-2-(2-nitrophenyl)acetonitrile** (**3j**) [84]. Yellow oil (10.1 mg, 19% yield); R*_f_* = 0.40 (petroleum ether/EtOAc = 10/1). ^1^H NMR (400 MHz, CDCl_3_): δ 8.04 (d, *J* = 8.0 Hz, 1H), 7.75 (d, *J* = 7.6 Hz, 1H), 7.69 (t, *J* = 7.2 Hz, 1H), 7.53 (t, *J* = 7.6 Hz, 1H), 7.22 (d, *J* = 8.4 Hz, 2H), 6.87 (d, *J* = 8.4 Hz, 2H), 6.11 (s, 1H), 3.79 (s, 3H) ppm. ^13^C NMR (100 MHz, CDCl_3_): δ 159.8, 147.7, 134.0, 130.9, 130.7, 129.5, 129.1, 126.0, 125.7, 118.8, 114.7, 55.3, 37.6 ppm.

**2-(4-methoxyphenyl)-2-(naphthalen-2-yl)acetonitrile** (**3k**) [85]. Colorless oil (53.5 mg, 98% yield). R*_f_* = 0.50 (petroleum ether/EtOAc = 20/1). ^1^H NMR (400 MHz, CDCl_3_): δ 7.87–7.81 (m, 4H), 7.53–7.48 (m, 2H), 7.34 (d, *J* = 8.4 Hz, 1H), 7.29 (d, *J* = 8.8 Hz, 2H), 6.89 (d, *J* = 8.4 Hz, 2H), 5.25 (s, 1H), 3.79 (s, 3H) ppm. ^13^C NMR (100 MHz, CDCl_3_): δ 159.5, 133.4, 133.2, 132.7, 129.2, 129.0, 128.0, 127.8, 127.7, 126.7, 126.6, 126.5, 125.2, 119.8, 114.5, 55.3, 42.0 ppm.

**2-(4-methoxyphenyl)-2-(thiophen-2-yl)acetonitrile** (**3l**). Colorless oil (33.8 mg, 74% yield); R*_f_* = 0.50 (petroleum ether/EtOAc = 20/1). ^1^H NMR (400 MHz, CDCl_3_): δ 7.31 (d, *J* = 8.4 Hz, 2H), 7.25 (d, *J* = 5.2 Hz, 1H), 7.06 (d, *J* = 3.2 Hz, 1H), 6.97–6.95 (m, 1H), 6.91 (d, *J* = 8.4 Hz, 2H), 5.30 (s, 1H), 3.80 (s, 3H) ppm. ^13^C NMR (100 MHz, CDCl_3_): δ 159.7, 139.1, 128.7, 127.5, 127.0, 126.5, 126.3, 119.0, 114.5, 55.3, 37.2 ppm. HRMS (MALDI-TOF/TOF) for C_13_H_12_NOS [M+H]^+^: calculated 230.0634, found 230.0631.

**2-(4-methoxyphenyl)propanenitrile** (**3m**) [48]. Colorless oil (23.3 mg, 72% yield); R*_f_* = 0.70 (petroleum ether/EtOAc = 20/1). ^1^H NMR (400 MHz, CDCl_3_): δ 7.27 (d, *J* = 8.4 Hz, 2H), 6.90 (d, *J* = 8.4 Hz, 2H), 3.85 (q, *J* = 7.6 Hz, 1H), 3.81 (s, 3H), 1.61 (d, *J* = 7.3 Hz, 3H) ppm. ^13^C NMR (100 MHz, CDCl_3_): δ 159.2, 129.0, 127.8, 121.8, 114.4, 55.3, 30.4, 21.5 ppm.

**2-(4-methoxyphenyl)butanenitrile** (**3n**) [86]. Colorless oil (17.5 mg, 50% yield); R*_f_* = 0.50 (petroleum ether/EtOAc = 20/1). ^1^H NMR (400 MHz, CDCl_3_): δ 7.23 (d, *J* = 8.4 Hz, 2H), 6.90 (d, *J* = 8.4 Hz, 2H), 3.81 (s, 3H), 3.68 (t, *J* = 7.2 Hz, 1H), 1.95–1.86 (m, 2H), 1.06 (t, *J* = 7.2 Hz, 3H) ppm. ^13^C NMR (100 MHz, CDCl_3_): δ 159.3, 128.4, 127.7, 121.0, 114.3, 55.3, 38.1, 29.2, 11.4 ppm.

**2-(4-methoxyphenyl)-3,3-dimethylbutanenitrile** (**3o**) [87]. White solid (31.1 mg, 76% yield); mp 50–52 °C; R*_f_* = 0.70 (petroleum ether/EtOAc = 20/1). ^1^H NMR (400 MHz, CDCl_3_): δ 7.19 (d, *J* = 6.8 Hz, 2H), 6.88 (d, *J* = 8.4 Hz, 2H), 3.82 (s, 3H), 3.51 (s, 1H), 1.04 (s, 9H) ppm. ^13^C NMR (100 MHz, CDCl_3_): δ 159.3, 130.4, 125.4, 120.6, 113.6, 55.3, 48.9, 35.1, 27.3 ppm.

**2-(3,4-dimethoxyphenyl)-2-phenylacetonitrile** (**3p**) [88]. Colorless oil (37.0 mg, 73% yield); R*_f_* = 0.50 (petroleum ether/EtOAc = 20/1). ^1^H NMR (400 MHz, CDCl_3_): δ 7.38–7.31 (m, 5H), 6.90–6.80 (m, 3H), 5.10 (s, 1H), 3.86 (s, 3H), 3.84 (s, 3H) ppm. ^13^C NMR (100 MHz, CDCl_3_): δ 149.4, 148.9, 136.0, 129.1, 128.13, 128.12, 127.5, 120.1, 119.8, 111.3, 110.7, 55.89, 55.88, 42.1 ppm.

**2-([1,1′-biphenyl]-4-yl)-2-(3,4-dimethoxyphenyl)acetonitrile** (**3q**) [48]. Colorless oil (59.3 mg, 90% yield); R*_f_* = 0.50 (petroleum ether/EtOAc = 20/1). ^1^H NMR (400 MHz, CDCl_3_): δ 7.60–7.55 (m, 4H), 7.45–7.39 (m, 4H), 7.37–7.33 (m, 1H), 6.92 (d, *J* = 8.4 Hz, 1H), 6.85 (d, *J* = 7.2 Hz, 2H), 5.13 (s, 1H), 3.86 (s, 3H), 3.85 (s, 3H) ppm. ^13^C NMR (100 MHz, CDCl_3_): δ 149.5, 149.0, 141.1, 140.1, 135.0, 128.8, 128.1, 128.0, 127.8, 127.6, 127.0, 120.2, 119.8, 111.4, 110.7, 56.0, 55.9, 41.8 ppm. HRMS (MALDI-TOF/TOF) for C_22_H_20_NO_2_ [M+H]^+^: calculated 330.1489, found 330.1386.

***N*-(4-(cyano(phenyl)methyl)phenyl)benzamide** (**3r**). White solid (41.6 mg, 67% yield); mp 196–198 °C; R*_f_* = 0.50 (petroleum ether/EtOAc = 20/1). ^1^H NMR (400 MHz, DMSO-d6): δ 10.35 (s, 1H), 7.98 (d, *J* = 7.6 Hz, 2H), 7.85 (d, *J* = 8.4 Hz, 2H), 7.65–7.61 (m, 1H), 7.58–7.55 (m, 2H), 7.48–7.39 (m, 7H), 5.81 (s, 1H) ppm. ^13^C NMR (100 MHz, DMSO-d6): δ 166.5, 139.8, 137.7, 135.6, 132.5, 130.1, 129.3, 128.8, 128.7, 128.5, 128.3, 121.8, 121.3, 41.2 ppm. HRMS (ESI) *m*/*z*: [M+H]^+^ calcd. for C_21_H_17_N_2_O: 313.1336; found: 313.1334.

**2,2-diphenylacetonitrile** (**3s**) [83]. White solid (13.1 mg, 34% yield); mp 70–72 °C; R*_f_* = 0.50 (petroleum ether/EtOAc = 15/1). ^1^H NMR (400 MHz, CDCl_3_): δ 7.39–7.30 (m, 10H), 5.14 (s, 1H) ppm. ^13^C NMR (100 MHz, CDCl_3_): δ 135.9, 129.2, 128.2, 127.7, 119.6, 42.6 ppm.

**6-methoxy-1,2,3,4-tetrahydronaphthalene-1-carbonitrile** (**3t**) [48]. Colorless oil (17.5 mg, 47% yield); R*_f_* = 0.50 (petroleum ether/EtOAc = 20/1). ^1^H NMR (400 MHz, CDCl_3_): δ 7.26 (d, *J* = 8.8 Hz, 1H), 6.78–6.75 (m, 1H), 6.64 (s, 1H), 3.92 (t, *J* = 6.4 Hz, 1H), 3.78 (s, 3H), 2.87–2.71 (m, 2H), 2.13–2.09 (m, 2H), 2.07–1.96 (m, 1H), 1.87–1.77 (m, 1H) ppm. ^13^C NMR (100 MHz, CDCl_3_): δ 159.1, 137.7, 129.9, 121.9, 114.2, 112.8, 55.2, 30.1, 28.7, 27.5, 20.7 ppm.

**(*E*)-2,4-diphenylbut-3-enenitrile** (**3u**) [89]. Colorless oil (34.0 mg, 78% yield); R*_f_* = 0.50 (petroleum ether/EtOAc = 20/1). ^1^H NMR (400 MHz, CDCl_3_): δ 7.42–7.26 (m, 10H), 6.81 (d, *J* = 15.6 Hz, 1H), 6.19 (dd, *J* = 16.0, 6.4 Hz, 1H), 4.69 (d, *J* = 6.4 Hz, 1H) ppm. ^13^C NMR (100 MHz, CDCl_3_): δ 135.4, 134.5, 133.2, 129.2, 128.7, 128.4, 127.5, 126.7, 123.2, 118.8, 40.0 ppm.

**2-(4-methoxyphenyl)acetonitrile** (**3v**) [90]. Colorless oil (17.0 mg, 23% yield, 0.5 mmol scale); R*_f_* = 0.50 (petroleum ether/EtOAc = 15/1). ^1^H NMR (400 MHz, CDCl_3_): δ 7.23 (d, *J* = 8.4 Hz, 2H), 6.90 (d, *J* = 8.4 Hz, 2H), 3.80 (s, 3H), 3.68 (s, 2H) ppm. ^13^C NMR (100 MHz, CDCl_3_): δ 159.3, 129.0, 121.8, 118.1, 114.5, 55.3, 22.8 ppm.

**2-(6-methoxynaphthalen-2-yl)propanenitrile** (**3w**) [91]. White solid (42.3 mg, 72% yield); mp 72–74 °C; R*_f_* = 0.70 (petroleum ether/EtOAc = 20/1). ^1^H NMR (400 MHz, CDCl_3_): δ 7.76–7.72 (m, 3H), 7.38 (dd, *J* = 8.4, 2.0 Hz, 1H), 7.18 (dd, *J* = 8.8, 2.4 Hz, 1H), 7.13 (d, *J* = 2.4 Hz, 1H), 4.02 (q, *J* = 7.2 Hz, 1H), 3.92 (s, 3H), 1.71 (d, *J* = 7.2 Hz, 3H) ppm. ^13^C NMR (100 MHz, CDCl_3_): δ 158.1, 134.0, 132.0, 129.3, 128.8, 127.9, 125.4, 124.9, 121.7, 119.6, 105.7, 55.3, 31.2, 21.4 ppm.

**5*H*-dibenzo[*a*,*d*][7]annulene-5-carbonitrile** (**3x**) [25]. Colorless oil (36.6 mg, 84% yield); R*_f_* = 0.50 (petroleum ether/EtOAc = 20/1). ^1^H NMR (400 MHz, CDCl_3_): δ 7.68 (s, 2H), 7.40 (t, *J* = 7.2 Hz, 2H), 7.35–7.28 (m, 4H), 7.11 (s, 2H), 4.72 (s, 1H) ppm. ^13^C NMR (100 MHz, CDCl_3_): δ 133.9, 132.3, 131.3, 129.2, 128.6, 127.8, 125.3, 118.3, 41.1 ppm.

**2-(2,5-dimethylphenyl)-2-(4-methoxyphenyl)acetonitrile** (**3y**) [92]. Colorless oil (47.2 mg, 94% yield); R*_f_* = 0.50 (petroleum ether/EtOAc = 20/1). ^1^H NMR (400 MHz, CDCl_3_): δ 7.22–7.17 (m, 3H), 7.07 (s, 2H), 6.87 (d, *J* = 8.8 Hz, 2H), 5.20 (s, 1H), 3.79 (s, 3H), 2.33 (s, 3H), 2.21 (s, 3H) ppm. ^13^C NMR (100 MHz, CDCl_3_): δ 159.3, 136.4, 133.6, 132.6, 131.1, 129.2, 129.1, 128.9, 127.1, 120.0, 114.4, 55.3, 39.1, 21.0, 18.9 ppm.

**2,2′-(oxybis((4-methoxyphenyl)methylene))bis(1,4-dimethylbenzene)** (**4y**). Colorless oil (17.9 mg, 38% yield); R*_f_* = 0.50 (petroleum ether/EtOAc = 15/1). ^1^H NMR (400 MHz, CDCl_3_): δ 7.46 (s, 1H), 7.39 (s, 1H), 7.20–7.18 (m, 4H), 6.98 (d, *J* = 10.8 Hz, 4H), 6.82 (t, *J* = 6.4 Hz, 4H), 5.46 (s, 1H), 5.45 (s, 1H), 3.77 (s, 6H), 2.33 (s, 3H), 2.31 (s, 3H), 1.96 (s, 6H) ppm. ^13^C NMR (100 MHz, CDCl_3_): δ 158.8, 158.6, 139.9, 139.6, 135.3, 134.0, 133.8, 133.0, 132.3, 130.4, 130.2, 129.1, 128.7, 128.3, 128.0, 127.8, 127.5, 113.6, 113.5, 76.9, 76.5, 55.2, 21.2, 21.2, 18.9, 18.8 ppm. HRMS (ESI) for C_32_H_34_O_3_Na [M+Na]^+^: calculated 489.2400, found 489.2414.

**2-(6-methoxynaphthalen-2-yl)propanoic acid (Naproxen)** [24]. White solid (31.8 mg, 69% yield); mp 156–158 °C; R*_f_* = 0.50 (petroleum ether/EtOAc/MeOH = 5/2/1). ^1^H NMR (400 MHz, CDCl_3_): δ 7.70–7.67 (m, 3H), 7.40 (d, *J* = 8.4 Hz, 1H), 7.14–7.10 (m, 2H), 3.90–3.85 (m, 4H), 1.58 (d, *J* = 6.8 Hz, 3H) ppm. ^13^C NMR (100 MHz, CDCl_3_): δ 179.9, 157.7, 135.1, 133.8, 129.3, 128.9, 127.2, 126.2, 126.1, 118.9, 105.7, 55.3, 45.3, 18.2 ppm.

## 4. Conclusions

In conclusion, by taking advantage of isonitriles as low-toxic CN surrogates in the metal-free cyanation of alcohols, an efficient and green method for the direct catalytic synthesis of α-aryl nitriles was developed (up to 98% yield). To the best of our knowledge, this is the first B(C_6_F_5_)_3_-catalyzed transformation of isonitriles. Control experiments support an S_N_1 pathway and rule out a concerted S_N_2 mechanism. The in situ-generated ether **4** can be converted to the desired α-aryl nitriles under the current catalytic system via cleavage of the C-O bond. The use of readily available starting materials, low catalyst loading, a broad substrate scope, ease of scale-up, and application in the synthesis of the precursors for naproxen and cytenamide make this approach very practical and attractive. With these advantages, we expect that this method will find wide applications in organic synthesis.

## Figures and Tables

**Table 1:** Optimization of reaction conditions ^[a]^.

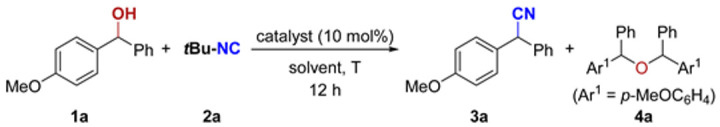						
Entry	Catalyst	Solvent	T [°C]	Conv. (%)	3a [%] ^[b]^	4a [%] ^[b]^
1	FeCl_3_	toluene	100	10	0	0
2	AlCl_3_	toluene	100	86	16	28
3	Cu(OTf)_2_	toluene	100	100	57	0
4	AgClO_4_	toluene	100	21	7	0
5	BF_3_·Et_2_O	toluene	100	48	24	11
6	B(C_6_F_5_)_3_	toluene	100	100	>99(98)	0
7	TsOH·H_2_O	toluene	100	84	32	29
8	Tf_2_NH	toluene	100	100	72	0
9	(PhO)_2_P(=O)OH	toluene	100	100	0	45
10	B(C_6_F_5_)_3_	toluene	80	70	20	30
11	B(C_6_F_5_)_3_	toluene	50	0	0	0
12	B(C_6_F_5_)_3_	DCE	reflux	100	93	0
13	B(C_6_F_5_)_3_	THF	reflux	100	82	0
15	B(C_6_F_5_)_3_	HFIP	reflux	50	20	0

^[a]^ Reaction conditions: **1a** (0.20 mmol), **2a** (0.30 mmol, 1.5 equiv), catalyst (10 mol%), solvent (2 mL), T °C, under N_2_ for 12 h. ^[b]^ The yields were determined by ^1^H NMR spectroscopy using CH_2_Br_2_ as the internal standard. The number in the parentheses is the isolated yield of **3a**.

## Data Availability

Not applicable.

## Supplementary Materials

The following supporting information can be downloaded at: https://www.mdpi.com/article/10.3390/molecules28052174/s1, ^1^H, ^13^C, ^19^F and HPLC spectra.

## Sample Availability

Samples of the compounds **3** are available from the authors.

## References

[1] Trost B.M. (1995). Atom Economy—A Challenge for Organic Synthesis: Homogeneous Catalysis Leads the Way. Angew. Chem. Int. Ed. Engl..

[2] Estopina-Duran S., Taylor J.E. (2021). Bronsted Acid-Catalysed Dehydrative Substitution Reactions of Alcohols. Chem. Eur. J..

[3] Huy P.H. (2020). Lewis Base Catalysis Promoted Nucleophilic Substitutions—Recent Advances and Future Directions. Eur. J. Org. Chem..

[4] Moran J., Dryzhakov M., Richmond E. (2016). Recent Advances in Direct Catalytic Dehydrative Substitution of Alcohols. Synthesis.

[5] Emer E., Sinisi R., Capdevila M.G., Petruzziello D., De Vincentiis F., Cozzi P.G. (2011). Direct Nucleophilic S_N_1-Type Reactions of Alcohols. Eur. J. Org. Chem..

[6] Bryan M.C., Dunn P.J., Entwistle D., Gallou F., Koenig S.G., Hayler J.D., Hickey M.R., Hughes S., Kopach M.E., Moine G. (2018). Key Green Chemistry Research Areas from a Pharmaceutical Manufacturers’ Perspective Revisited. Green Chem..

[7] Zhang W., Wang F., McCann S.D., Wang D., Chen P., Stahl S.S., Liu G. (2016). Enantioselective Cyanation of Benzylic C–H Bonds Via Copper-Catalyzed Radical Relay. Science.

[8] Wang D., Zhu N., Chen P., Lin Z., Liu G. (2017). Enantioselective Decarboxylative Cyanation Employing Cooperative Photoredox Catalysis and Copper Catalysis. J. Am. Chem. Soc..

[9] Yuan Y., Yang J., Zhang J. (2023). Cu-Catalyzed Enantioselective Decarboxylative Cyanation Via the Synergistic Merger of Photocatalysis and Electrochemistry. Chem. Sci..

[10] Culkin D.A., Hartwig J.F. (2003). Palladium-Catalyzed α-Arylation of Carbonyl Compounds and Nitriles. Acc. Chem. Res..

[11] Wu G., Deng Y., Wu C., Zhang Y., Wang J. (2014). Synthesis of α-Aryl Esters and Nitriles: Deaminative Coupling of α-Aminoesters and α-Aminoacetonitriles with Arylboronic Acids. Angew. Chem. Int. Ed..

[12] Falk A., Goderz A.L., Schmalz H.G. (2013). Enantioselective Nickel-Catalyzed Hydrocyanation of Vinylarenes Using Chiral Phosphine-Phosphite Ligands and TMS-CN as a Source of HCN. Angew. Chem. Int. Ed..

[13] Singh D.K., Prasad S.S., Kim J., Kim I. (2019). One-Pot, Three-Component Approach to Diarylacetonitriles. Org. Chem. Front..

[14] Tan J.-P., Chen Y., Ren X., Guo Y., Yi B., Zhang H., Gao G., Wang T. (2022). In Situ Phosphonium-Containing Lewis Base-Catalyzed 1,6-Cyanation Reaction: A Facile Way to Obtain α-Diaryl and α-Triaryl Acetonitriles. Org. Chem. Front..

[15] Kadunce N.T., Reisman S.E. (2015). Nickel-Catalyzed Asymmetric Reductive Cross-Coupling between Heteroaryl Iodides and α-Chloronitriles. J. Am. Chem. Soc..

[16] Jiao Z., Chee K.W., Zhou J.S. (2016). Palladium-Catalyzed Asymmetric α-Arylation of Alkylnitriles. J. Am. Chem. Soc..

[17] Liu R.Y., Bae M., Buchwald S.L. (2018). Mechanistic Insight Facilitates Discovery of a Mild and Efficient Copper-Catalyzed Dehydration of Primary Amides to Nitriles Using Hydrosilanes. J. Am. Chem. Soc..

[18] Fang X., Yu P., Morandi B. (2016). Catalytic Reversible Alkene-Nitrile Interconversion through Controllable Transfer Hydrocyanation. Science.

[19] Goswami P., Singh G., Vijaya Anand R. (2017). *N*-Heterocyclic Carbene Catalyzed 1,6-Conjugate Addition of Me_3_Si-CN to *para*-Quinone Methides and Fuchsones: Access to α-Arylated Nitriles. Org. Lett..

[20] Michel N.W.M., Jeanneret A.D.M., Kim H., Rousseaux S.A.L. (2018). Nickel-Catalyzed Cyanation of Benzylic and Allylic Pivalate Esters. J. Org. Chem..

[21] Fleming F.F., Yao L., Ravikumar P.C., Funk L., Shook B.C. (2010). Nitrile-Containing Pharmaceuticals: Efficacious Roles of the Nitrile Pharmacophore. J. Med. Chem..

[22] Yamamoto T., Kato S., Aoki D., Otsuka H. (2021). A Diarylacetonitrile as a Molecular Probe for the Detection of Polymeric Mechanoradicals in the Bulk State through a Radical Chain-Transfer Mechanism. Angew. Chem. Int. Ed..

[23] Sica D.A., Prisant L.M. (2007). Pharmacologic and Therapeutic Considerationsin Hypertension Therapy with Calcium Channel Blockers: Focus on Verapamil. J. Clin. Hypertens..

[24] Harrington P.J., Lodewijk E. (1997). Twenty Years of Naproxen Technology. Org. Process Res. Dev..

[25] Bedford C.T. (2018). An Efficient, Large-Scale Synthesis of Cytenamide. J. Chem. Res..

[26] Brett D., Downie I.M., Lee J.B. (1967). Sugars with Potential Antiviral Activity. Conversion of Hydroxy Compounds to Nitriles. J. Org. Chem..

[27] Davis R., Untch K.G. (1981). Direct One-Step Conversion of Alcohols into Nitriles. J. Org. Chem..

[28] Iranpoor N., Firouzabadi H., Akhlaghinia B., Nowrouzi N. (2004). Conversion of Alcohols, Thiols, and Trimethysilyl Ethers to Alkyl Cyanides Using Triphenylphosphine/2,3-Dichloro-5,6-Dicyanobenzoquinone/*n*-Bu_4_NCN. J. Org. Chem..

[29] Soltani Rad M.N., Khalafi-Nezhad A., Behrouz S., Faghihi M.A. (2007). A Simple One-Pot Procedure for the Direct Conversion of Alcohols into Alkyl Nitriles Using TsIm. Tetrahedron Lett..

[30] Aesa M.C., Baán G., Novák L., Szántay C. (1995). Preparation of Unsaturated Nitriles by the Modification of Mitsunobu-Wilk Procedure. Synth. Commun..

[31] Chen G., Wang Z., Wu J., Ding K. (2008). Facile Preparation of α-Aryl Nitriles by Direct Cyanation of Alcohols with TMSCN under the Catalysis of InX_3_. Org. Lett..

[32] Trillo P., Baeza A., Nájera C. (2013). Direct Nucleophilic Substitution of Free Allylic Alcohols in Water Catalyzed by FeCl_3_⋅6 H_2_O: Which Is the Real Catalyst?. ChemCatChem.

[33] Theerthagiri P., Lalitha A. (2012). Zn(OTf)_2_—Catalyzed Direct Cyanation of Benzylic Alcohols—a Novel Synthesis of α-Aryl Nitriles. Tetrahedron Lett..

[34] Wang J., Masui Y., Onaka M. (2011). Direct Synthesis of Nitriles from Alcohols with Trialkylsilyl Cyanide Using Brønsted Acid Montmorillonite Catalysts. ACS Catal..

[35] Rajagopal G., Kim S.S. (2009). Synthesis of α-Aryl Nitriles through B(C_6_F_5_)_3_-Catalyzed Direct Cyanation of α-Aryl Alcohols and Thiols. Tetrahedron.

[36] Bhor M.D., Panda A.G., Nandurkar N.S., Bhanage B.M. (2008). Synthesis of Alkyl Iodides/Nitriles from Carbonyl Compounds Using Novel Ruthenium Tris(2,2,6,6-Tetramethyl-3,5-Heptanedionate) as Catalyst. Tetrahedron Lett..

[37] Yu R., Rajasekar S., Fang X. (2020). Enantioselective Nickel-Catalyzed Migratory Hydrocyanation of Nonconjugated Dienes. Angew. Chem. Int. Ed..

[38] Gao J., Jiao M., Ni J., Yu R., Cheng G.J., Fang X. (2021). Nickel-Catalyzed Migratory Hydrocyanation of Internal Alkenes: Unexpected Diastereomeric-Ligand-Controlled Regiodivergence. Angew. Chem. Int. Ed..

[39] Sun F., Wang T., Cheng G.-J., Fang X. (2021). Enantioselective Nickel-Catalyzed Hydrocyanative Desymmetrization of Norbornene Derivatives. ACS Catal..

[40] Ding S., Jiao N. (2011). Direct Transformation of *N*,*N*-Dimethylformamide to −CN: Pd-Catalyzed Cyanation of Heteroarenes Via C–H Functionalization. J. Am. Chem. Soc..

[41] Ren X., Chen J., Chen F., Cheng J. (2011). The Palladium-Catalyzed Cyanation of Indole C-H Bonds with the Combination of NH_4_HCO_3_ and DMSO as a Safe Cyanide Source. Chem. Commun..

[42] Fan C., Zhou Q.-L. (2021). Nickel-Catalyzed Group Transfer of Radicals Enables Hydrocyanation of Alkenes and Alkynes. Chem. Catal..

[43] Cui J., Song J., Liu Q., Liu H., Dong Y. (2018). Transition-Metal-Catalyzed Cyanation by Using an Electrophilic Cyanating Agent, *N*-Cyano-*N*-Phenyl-*p*-Toluenesulfonamide (NCTS). Chem. Asian J..

[44] Ito Y., Kato H., Imai H., Saegusa T. (1982). A Novel Conjugate Hydrocyanation with TiCl_4_-*tert*-Butyl Isocyanide. J. Am. Chem. Soc..

[45] Xu S., Huang X., Hong X., Xu B. (2012). Palladium-Assisted Regioselective C–H Cyanation of Heteroarenes Using Isonitrile as Cyanide Source. Org. Lett..

[46] Peng J., Zhao J., Hu Z., Liang D., Huang J., Zhu Q. (2012). Palladium-Catalyzed C(Sp^2^)–H Cyanation Using Tertiary Amine Derived Isocyanide as a Cyano Source. Org. Lett..

[47] Dewanji A., van Dalsen L., Rossi-Ashton J.A., Gasson E., Crisenza G.E.M., Procter D.J. (2022). A General Arene C-H Functionalization Strategy Via Electron Donor-Acceptor Complex Photoactivation. Nat. Chem..

[48] Tang S., Guillot R., Grimaud L., Vitale M.R., Vincent G. (2022). Electrochemical Benzylic C-H Functionalization with Isocyanides. Org. Lett..

[49] Jiang X., Wang J.-M., Zhang Y., Chen Z., Zhu Y.-M., Ji S.-J. (2015). Synthesis of Aryl Nitriles by Palladium-Assisted Cyanation of Aryl Iodides Using *tert*-Butyl Isocyanide as Cyano Source. Tetrahedron.

[50] Higashimae S., Kurata D., Kawaguchi S.I., Kodama S., Sonoda M., Nomoto A., Ogawa A. (2018). Palladium-Catalyzed Cyanothiolation of Internal Alkynes Using Organic Disulfides and *tert*-Butyl Isocyanide. J. Org. Chem..

[51] Shirsath S.R., Shinde G.H., Shaikh A.C., Muthukrishnan M. (2018). Accessing α-Arylated Nitriles Via BF_3_.OEt_2_ Catalyzed Cyanation of *para*-Quinone Methides Using *tert*-Butyl Isocyanide as a Cyanide Source. J. Org. Chem..

[52] Nauth A.M., Opatz T. (2019). Non-Toxic Cyanide Sources and Cyanating Agents. Org. Biomol. Chem..

[53] Kim J., Kim H.J., Chang S. (2012). Synthesis of Aromatic Nitriles Using Nonmetallic Cyano-Group Sources. Angew. Chem. Int. Ed..

[54] Wang W., Liu T., Ding C.-H., Xu B. (2021). C(Sp^3^)–H Functionalization with Isocyanides. Org. Chem. Front..

[55] Wang J., Li D., Li J., Zhu Q. (2021). Advances in Palladium-Catalysed Imidoylative Cyclization of Functionalized Isocyanides for the Construction of N-Heterocycles. Org. Biomol. Chem..

[56] Giustiniano M., Basso A., Mercalli V., Massarotti A., Novellino E., Tron G.C., Zhu J. (2017). To Each His Own: Isonitriles for All Flavors. Functionalized Isocyanides as Valuable Tools in Organic Synthesis. Chem. Soc. Rev..

[57] Song B., Xu B. (2017). Metal-Catalyzed C-H Functionalization Involving Isocyanides. Chem. Soc. Rev..

[58] Zhang B., Studer A. (2015). Recent Advances in the Synthesis of Nitrogen Heterocycles via Radical Cascade Reactions Using Isonitriles as Radical Acceptors. Chem. Soc. Rev..

[59] Boyarskiy V.P., Bokach N.A., Luzyanin K.V., Kukushkin V.Y. (2015). Metal-Mediated and Metal-Catalyzed Reactions of Isocyanides. Chem. Rev..

[60] Qiu G., Ding Q., Wu J. (2013). Recent Advances in Isocyanide Insertion Chemistry. Chem. Soc. Rev..

[61] Dömling A. (2006). Recent Developments in Isocyanide Based Multicomponent Reactions in Applied Chemistry. Chem. Rev..

[62] Fang H., Oestreich M. (2020). Defunctionalisation Catalysed by Boron Lewis Acids. Chem. Sci..

[63] Ma Y., Lou S.J., Hou Z. (2021). Electron-Deficient Boron-Based Catalysts for C-H Bond Functionalisation. Chem. Soc. Rev..

[64] Basak S., Winfrey L., Kustiana B.A., Melen R.L., Morrill L.C., Pulis A.P. (2021). Electron Deficient Borane-Mediated Hydride Abstraction in Amines: Stoichiometric and Catalytic Processes. Chem. Soc. Rev..

[65] Kumar G., Roy S., Chatterjee I. (2021). Tris(Pentafluorophenyl)Borane Catalyzed C-C and C-Heteroatom Bond Formation. Org. Biomol. Chem..

[66] Oestreich M., Hermeke J., Mohr J. (2015). A Unified Survey of Si-H and H-H Bond Activation Catalysed by Electron-Deficient Boranes. Chem. Soc. Rev..

[67] Melen R.L. (2014). Applications of Pentafluorophenyl Boron Reagents in the Synthesis of Heterocyclic and Aromatic Compounds. Chem. Commun..

[68] Erker G. (2005). Tris(Pentafluorophenyl)Borane: A Special Boron Lewis Acid for Special Reactions. Dalton Trans..

[69] Meng S.-S., Tang X., Luo X., Wu R., Zhao J.-L., Chan A.S.C. (2019). Borane-Catalyzed Chemoselectivity-Controllable N-Alkylation and *ortho* C-Alkylation of Unprotected Arylamines Using Benzylic Alcohols. ACS Catal..

[70] Meng S.S., Wang Q., Huang G.B., Lin L.R., Zhao J.L., Chan A.S.C. (2018). B(C_6_F_5_)_3_ Catalyzed Direct Nucleophilic Substitution of Benzylic Alcohols: An Effective Method of Constructing C-O, C-S and C-C Bonds from Benzylic Alcohols. RSC Adv..

[71] Chen X., Patel K., Marek I. (2023). Stereoselective Construction of Tertiary Homoallyl Alcohols and Ethers by Nucleophilic Substitution at Quaternary Carbon Stereocenters. Angew. Chem. Int. Ed..

[72] San H.H., Huang J., Lei Aye S., Tang X.Y. (2021). Boron-Catalyzed Dehydrative Friedel-Crafts Alkylation of Arenes Using *β*-Hydroxyl Ketone as MVK Precursor. Adv. Synth. Catal..

[73] Guru M.M., Thorve P.R., Maji B. (2020). Boron-Catalyzed *N*-Alkylation of Arylamines and Arylamides with Benzylic Alcohols. J. Org. Chem..

[74] Rubin M., Gevorgyan V. (2001). B(C_6_F_5_)_3_-Catalyzed Allylation of Secondary Benzyl Acetates with Allylsilanes. Org. Lett..

[75] Dryzhakov M., Hellal M., Wolf E., Falk F.C., Moran J. (2015). Nitro-Assisted Bronsted Acid Catalysis: Application to a Challenging Catalytic Azidation. J. Am. Chem. Soc..

[76] Xiao Y., Tang L., Xu T.T., Feng J.J. (2022). Boron Lewis Acid Catalyzed Intermolecular Trans-Hydroarylation of Ynamides with Hydroxyarenes. Org. Lett..

[77] Lin T.-Y., Wu H.-H., Feng J.-J., Zhang J. (2017). Design and Enantioselective Synthesis of *β*-Vinyl Tryptamine Building Blocks for Construction of Privileged Chiral Indole Scaffolds. ACS Catal..

[78] Feng J.-J., Zhang J. (2017). Rhodium-Catalyzed Stereoselective Intramolecular Tandem Reaction of Vinyloxiranes with Alkynes: Atom- and Step-Economical Synthesis of Multifunctional Mono-, Bi-, and Tricyclic Compounds. ACS Catal..

[79] Zhu C.Z., Feng J.J., Zhang J. (2017). Rhodium(I)-Catalyzed Intermolecular Aza-[4+3] Cycloaddition of Vinyl Aziridines and Dienes: Atom-Economical Synthesis of Enantiomerically Enriched Functionalized Azepines. Angew. Chem. Int. Ed..

[80] Jacobsen H., Berke H., Döring S., Kehr G., Erker G., Fröhlich R., Meyer O. (1999). Lewis Acid Properties of Tris(Pentafluorophenyl)Borane. Structure and Bonding in L−B(C_6_F_5_)_3_ Complexes. Organometallics.

[81] Tumanov V.V., Tishkov A.A., Mayr H. (2007). Nucleophilicity Parameters for Alkyl and Aryl Isocyanides. Angew. Chem. Int. Ed..

[82] Zhou B., Zhang Y.Q., Zhang K., Yang M.Y., Chen Y.B., Li Y., Peng Q., Zhu S.F., Zhou Q.L., Ye L.W. (2019). Stereoselective Synthesis of Medium Lactams Enabled by Metal-Free Hydroalkoxylation/Stereospecific [1,3]-Rearrangement. Nat. Commun..

[83] Nambo M., Yar M., Smith J.D., Crudden C.M. (2015). The Concise Synthesis of Unsymmetric Triarylacetonitriles via Pd-Catalyzed Sequential Arylation: A New Synthetic Approach to Tri- and Tetraarylmethanes. Org. Lett..

[84] Choi I., Chung H., Park J.W., Chung Y.K. (2016). Active and Recyclable Catalytic Synthesis of Indoles by Reductive Cyclization of 2-(2-Nitroaryl)Acetonitriles in the Presence of Co-Rh Heterobimetallic Nanoparticles with Atmospheric Hydrogen under Mild Conditions. Org. Lett..

[85] Asai K., Hirano K., Miura M. (2020). Divergent Synthesis of Isonitriles and Nitriles by Palladium-Catalyzed Benzylic Substitution with TMSCN. J. Org. Chem..

[86] Shu X., Jiang Y.Y., Kang L., Yang L. (2020). Ni-Catalyzed hydrocyanation of alkenes with formamide as the cyano source. Green Chem..

[87] Weinreb S., Sengupta R. (2012). A One-Pot Umpolung Method for Preparation of α-Aryl Nitriles from α-Chloro Aldoximes via Organocuprate Additions to Transient Nitrosoalkenes. Synthesis.

[88] Aksenov A.V., Aksenov N.A., Dzhandigova Z.V., Aksenov D.A., Rubin M. (2015). Nitroalkenes as surrogates for cyanomethylium species in a one-pot synthesis of non-symmetric diarylacetonitriles. RSC Adv..

[89] Liu S.W., Meng L.L., Zeng X.J., Hammond G.B., Xu B. (2021). Synthesis of Acrylonitriles via Mild Base Promoted Tandem Nucleophilic Substitution-Isomerization of α-Cyanohydrin Methanesulfonates. Chin. J. Chem..

[90] Lindsay-Scott P.J., Clarke A., Richardson J. (2015). Two-Step Cyanomethylation Protocol: Convenient Access to Functionalized Aryl- and Heteroarylacetonitriles. Org. Lett..

[91] Chen Y., Xu L.T., Jiang Y.W., Ma D.W. (2021). Assembly of α-(Hetero)aryl Nitriles via Copper-Catalyzed Coupling Reactions with (Hetero)aryl Chlorides and Bromides. Angew. Chem. Int. Ed..

[92] Yuen O.Y., Chen X., Wu J., So C.M. (2020). Palladium-Catalyzed Direct α-Arylation of Arylacetonitriles with Aryl Tosylates and Mesylates. Eur. J. Org. Chem..

