# Supplementary material for: Boron Lewis Acid Catalysis Enables the Direct Cyanation of Benzyl Alcohols by Means of Isonitrile as Cyanide Source
Source: Molecules. 2023 Feb 26;28(5):2174. doi: 10.3390/molecules28052174 (PMC10004367; doi:10.3390/molecules28052174)
Supplement: Supplementary file 1 [file molecules-28-02174-s001.zip › molecules-2215511-supplementary.pdf]

Supporting Information

Boron Lewis Acid Catalysis Enables the Direct Cyanation of Benzyl Alcohols by means of Isonitrile as Cyanide Source

Tong-Tong Xu, Jin-Lan Zhou, Guang-Yuan Cong, Jiang-Yi-Hui Sheng, Shi-Qi Wang, Yating Ma, and Jian-Jun Feng*

State Key Laboratory of Chemo/Biosensing and Chemometrics, Advanced Catalytic Engineering Research Center of the Ministry of Education, College of Chemistry and Chemical Engineering, Hunan University, Changsha, Hunan 410082, P. R. China

jianjunfeng@hnu.edu.cn

NMR Spectra

^{1}H and ^{13}C NMR Spectra for Compound 3a:

^{1}H NMR (400 MHz, $CDCl_3$)

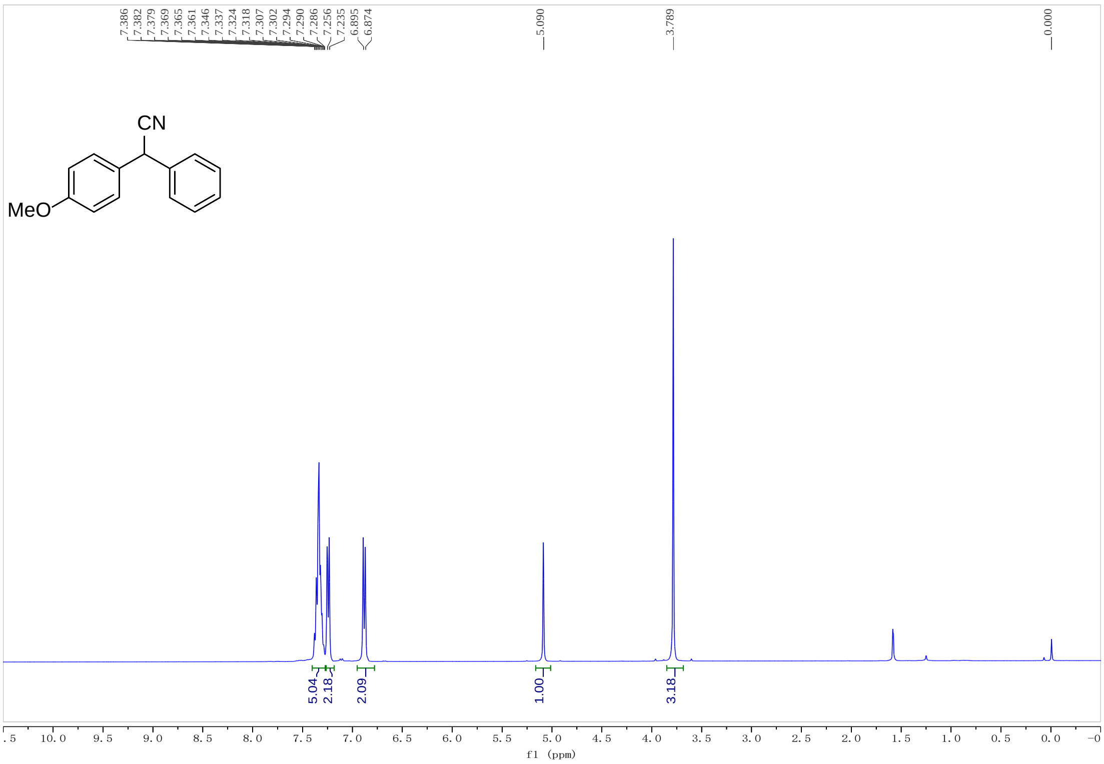

^{13}C NMR (100 MHz, $CDCl_3$)

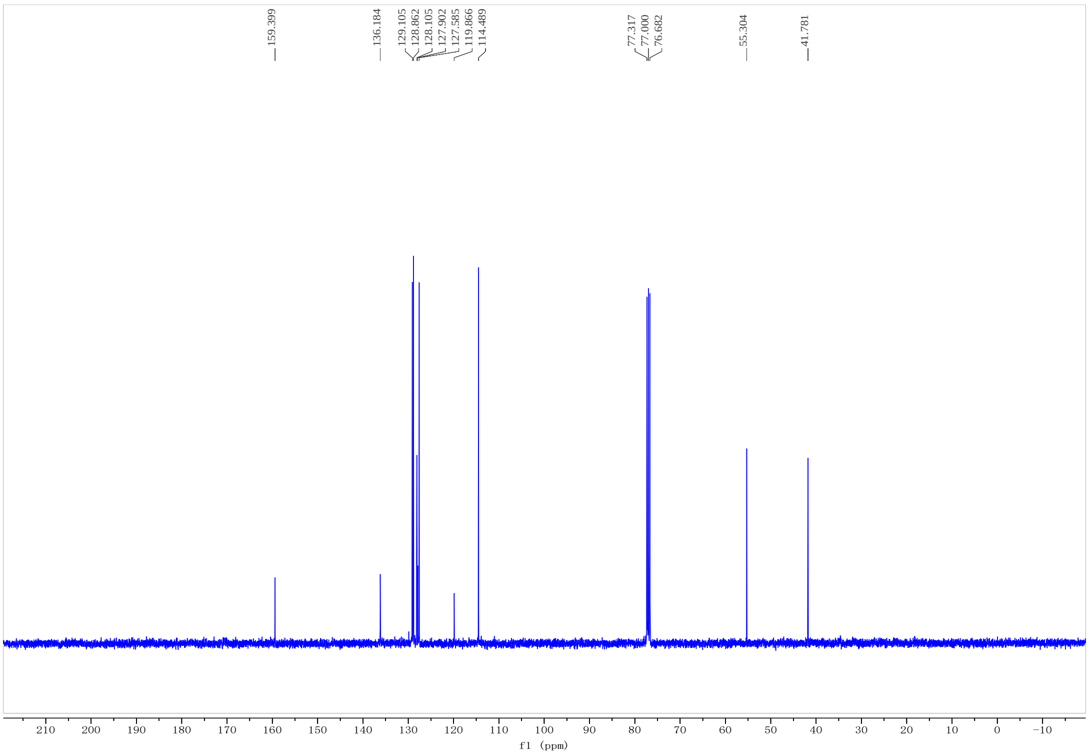

^{1}H and ^{13}C NMR Spectra for Compound 3b:

^{1}H NMR (400 MHz, $CDCl_3$)

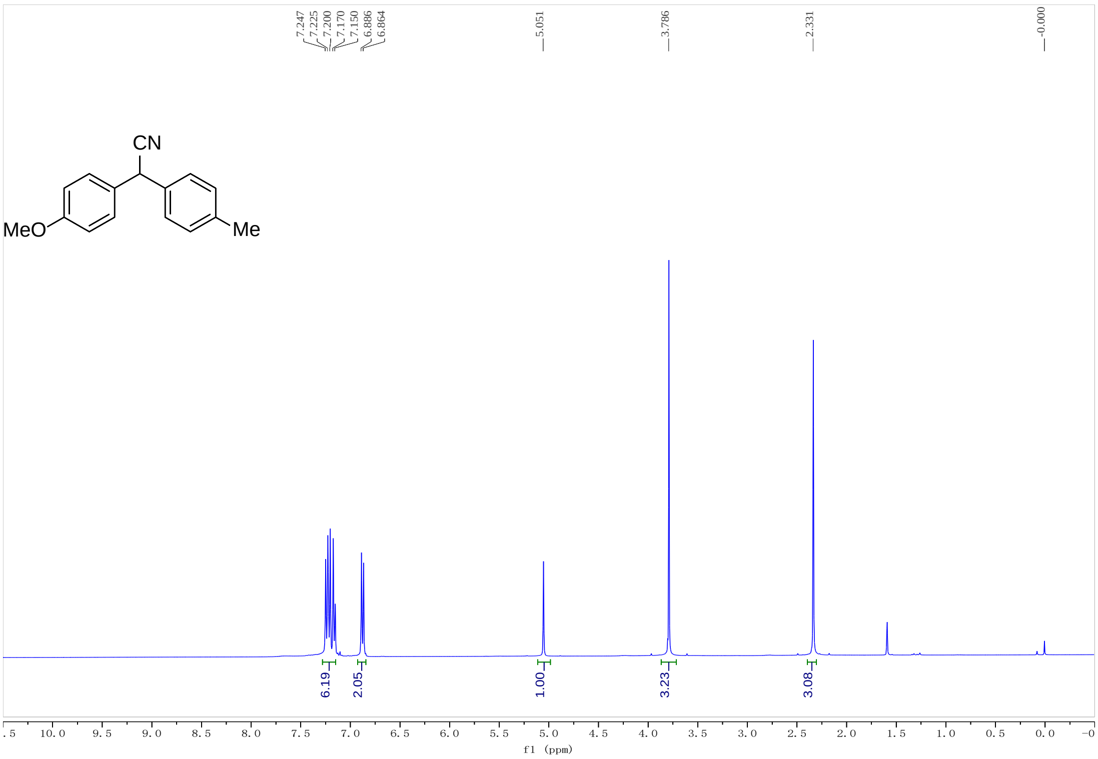

^{13}C NMR (100 MHz, $CDCl_3$)

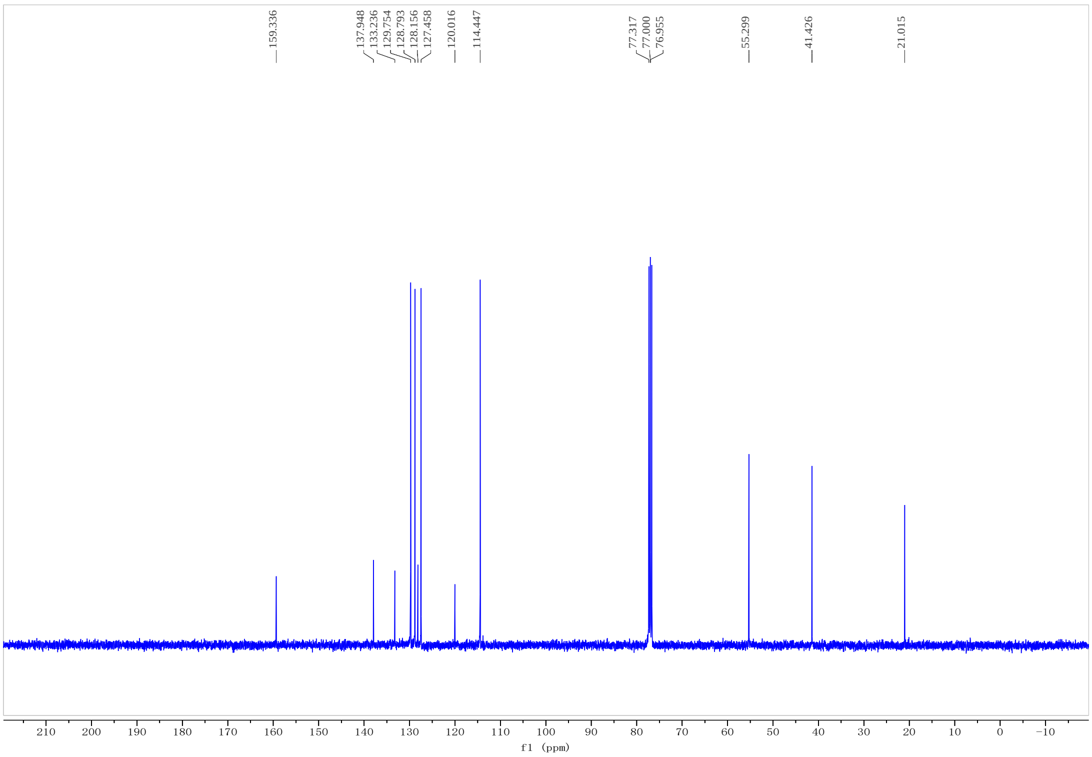

^{1}H and ^{13}C NMR Spectra for Compound 3c:

^{1}H NMR (400 MHz, $CDCl_3$)

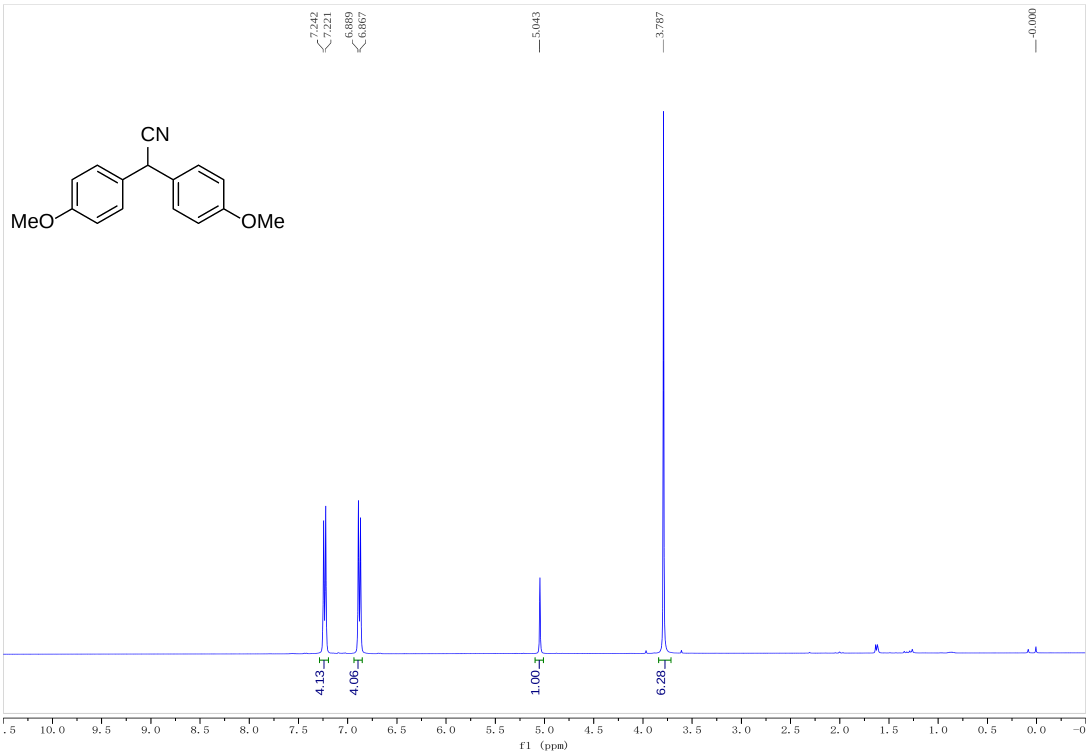

^{13}C NMR (100 MHz, $CDCl_3$)

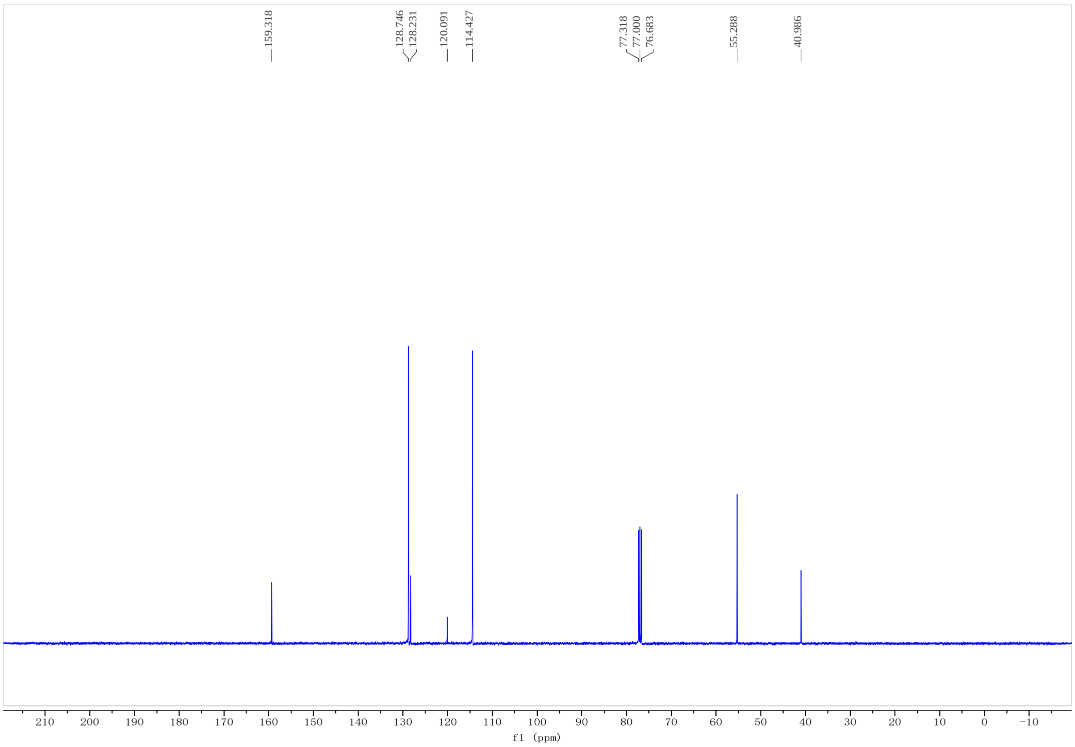

^{1}H and ^{13}C NMR Spectra for Compound 3d:

^{1}H NMR (400 MHz, $CDCl_3$)

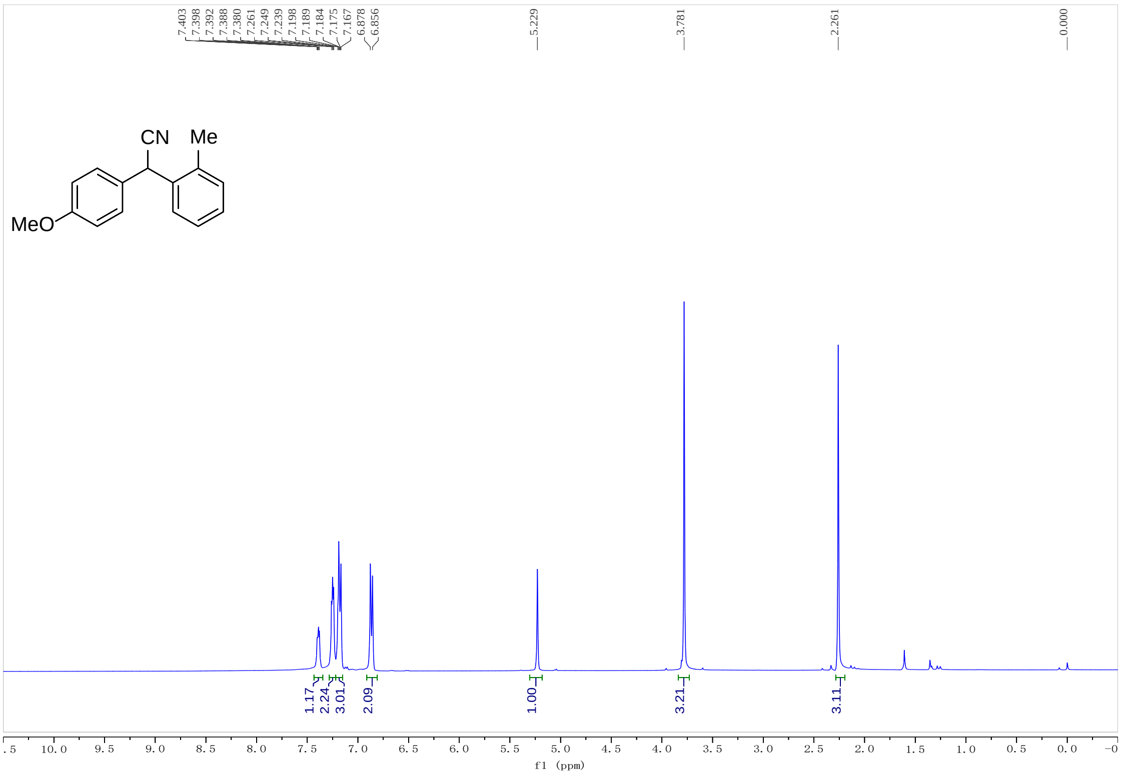

^{13}C NMR (100 MHz, $CDCl_3$)

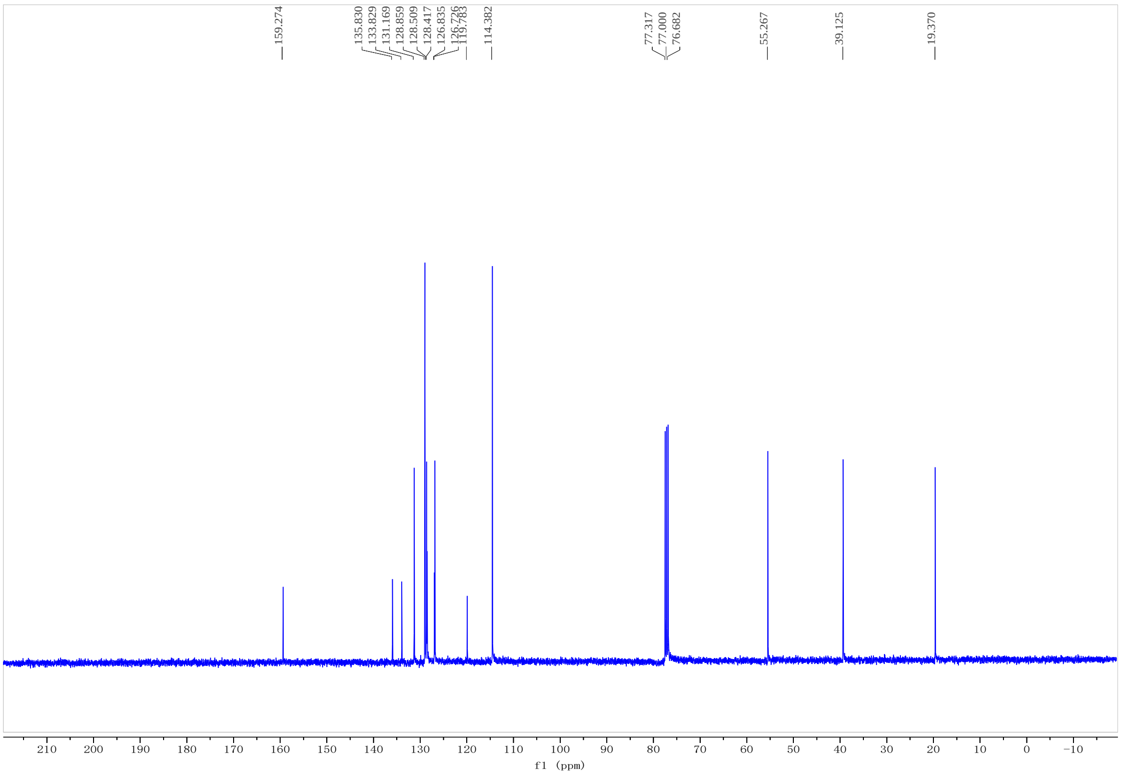

^{1}H and ^{13}C NMR Spectra for Compound 3e:

^{1}H NMR (400 MHz, $CDCl_3$)

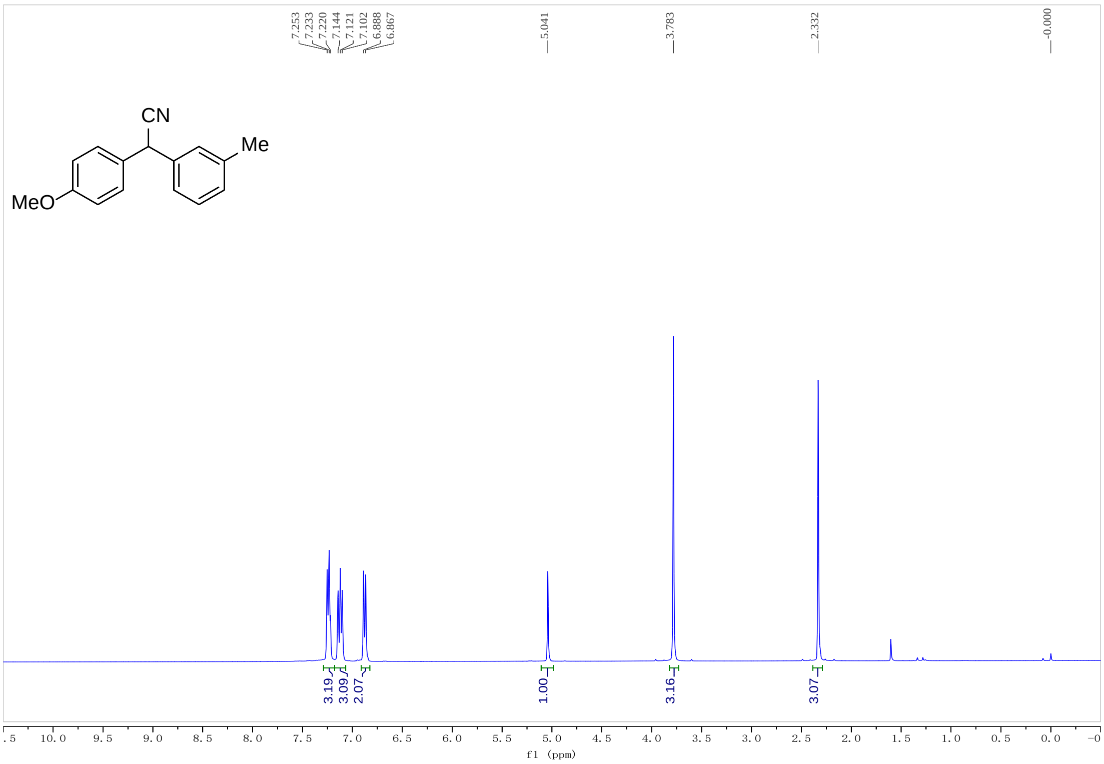

^{13}C NMR (100 MHz, $CDCl_3$)

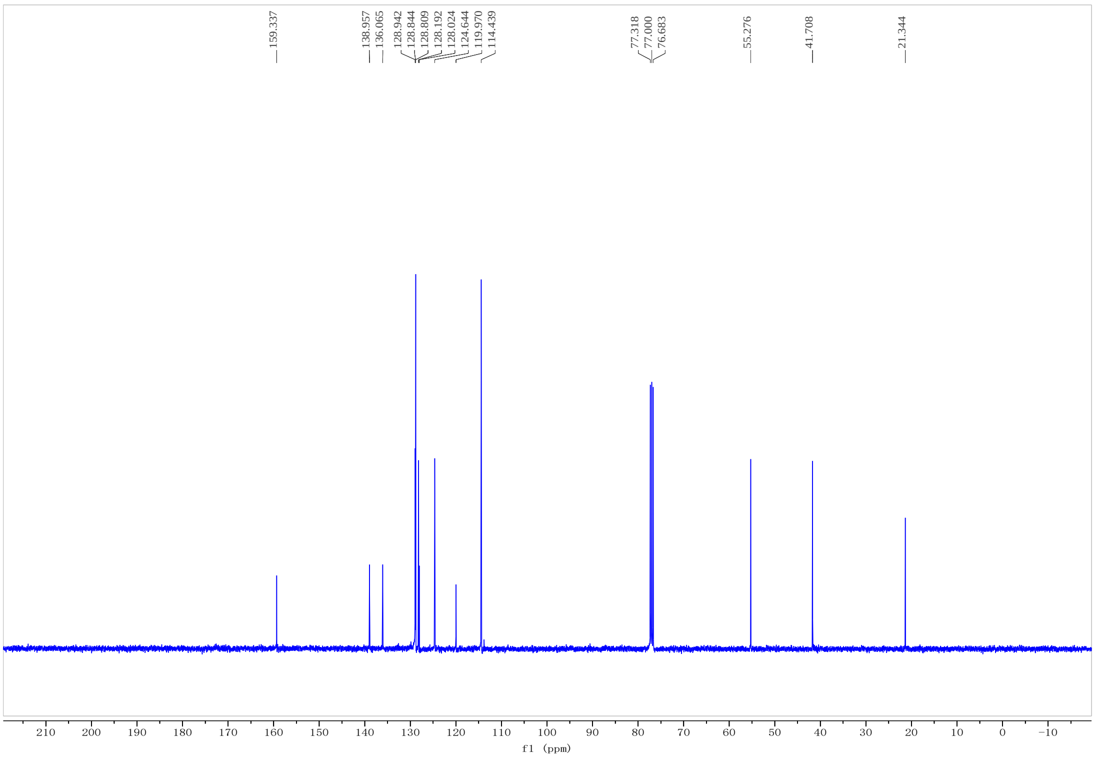

^{1}H and ^{13}C NMR Spectra for Compound 3f:

^{1}H NMR (400 MHz, $CDCl_3$)

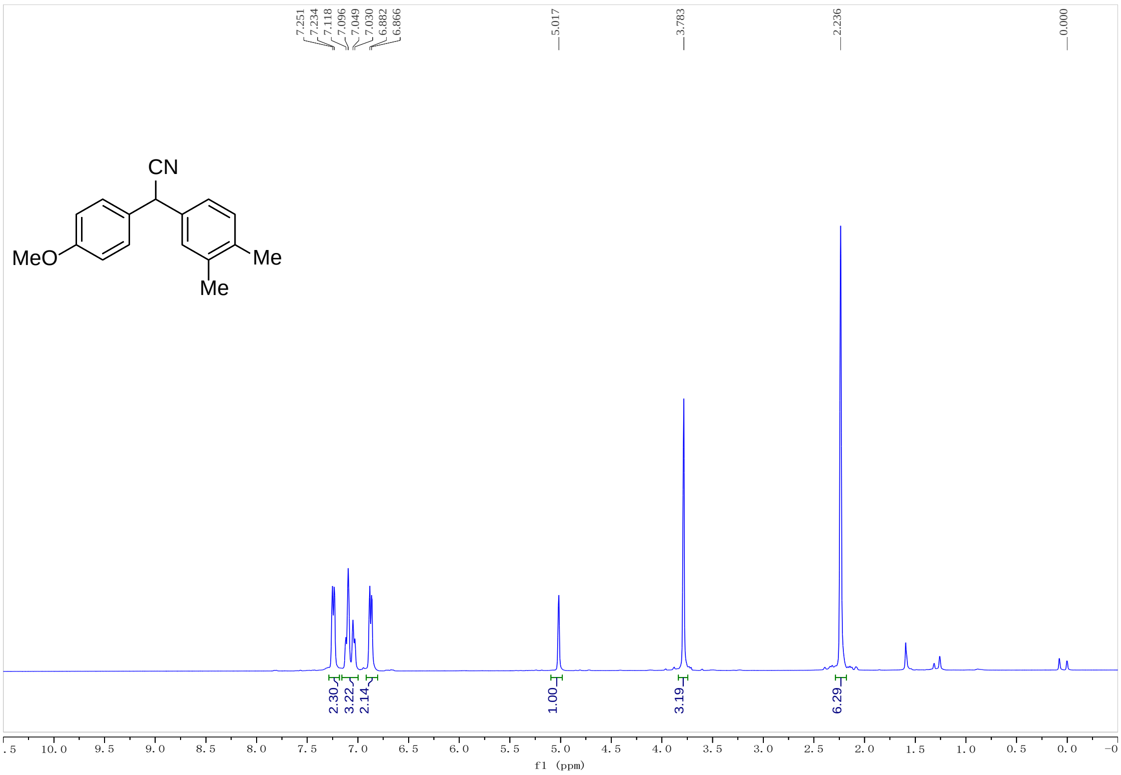

^{13}C NMR (100 MHz, $CDCl_3$)

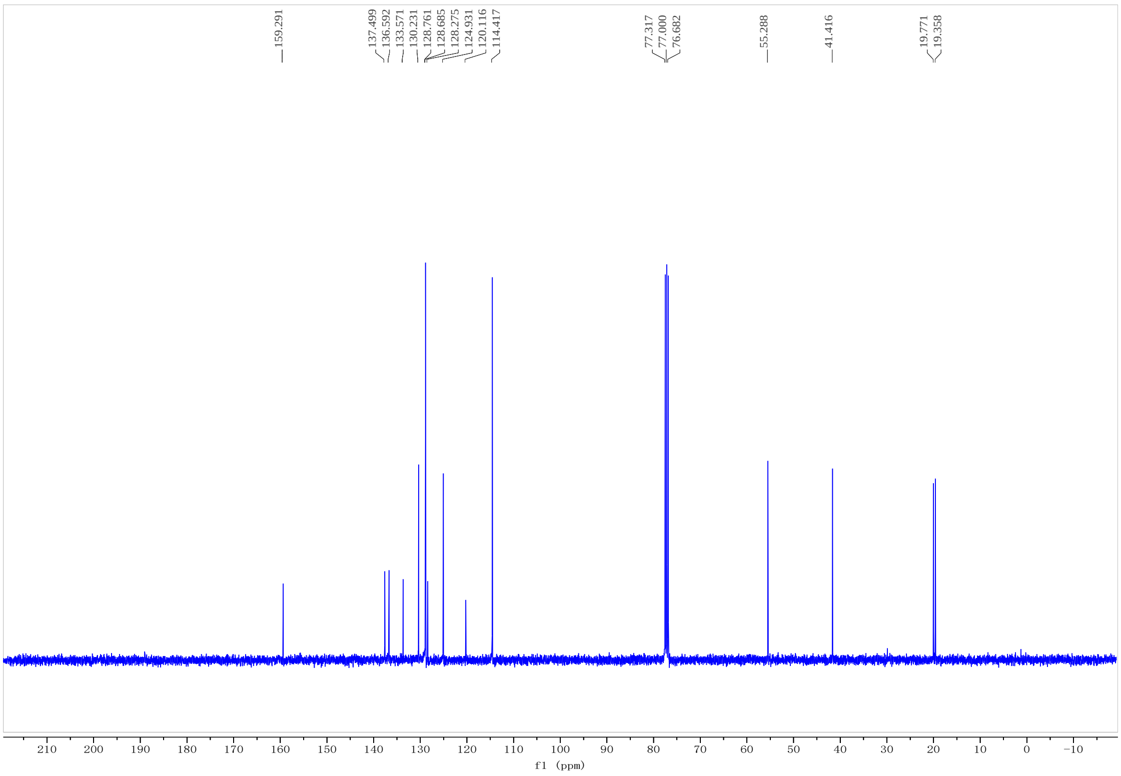

^{1}H and ^{13}C NMR Spectra for Compound 3g:

^{1}H NMR (400 MHz, $CDCl_3$)

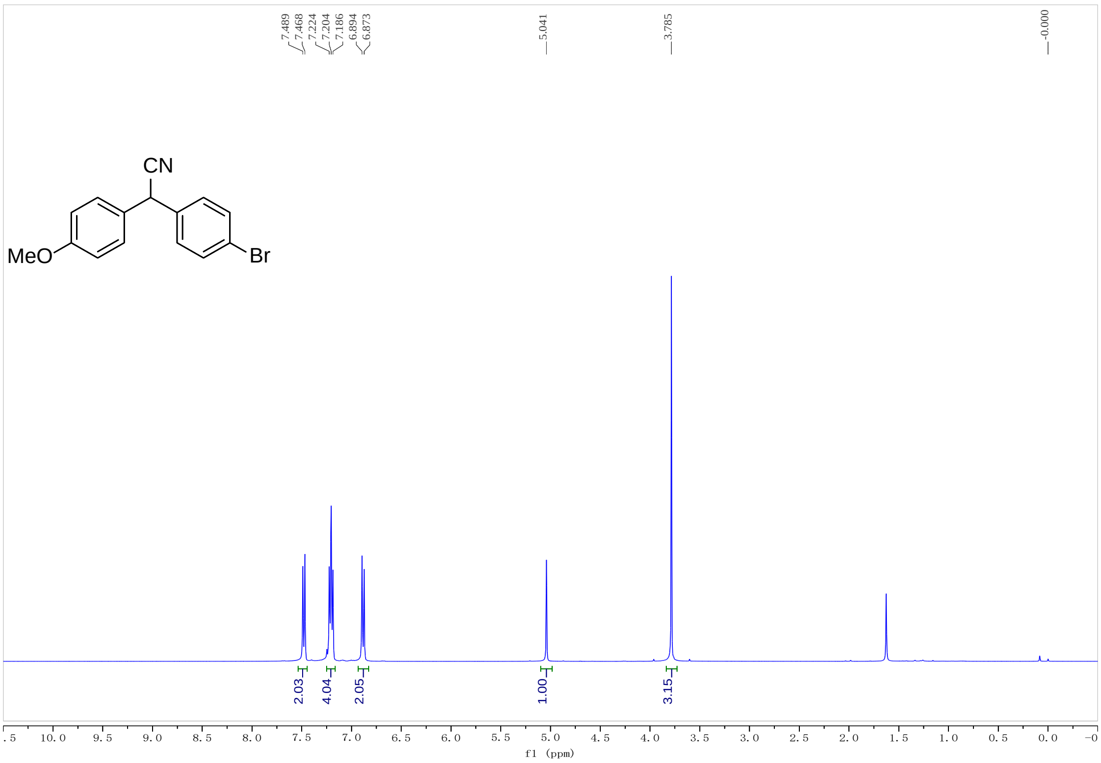

^{13}C NMR (100 MHz, $CDCl_3$)

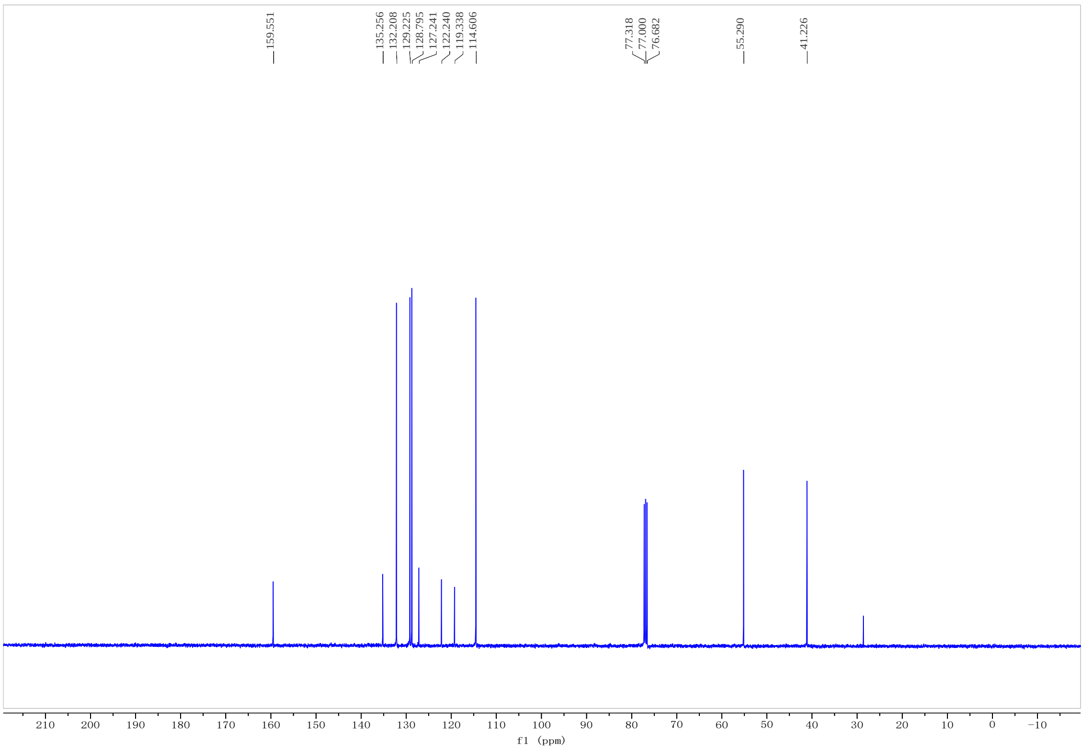

^{1}H, ^{13}C and ^{19}F NMR Spectra for Compound 3h:

^{1}H NMR (400 MHz, $CDCl_3$)

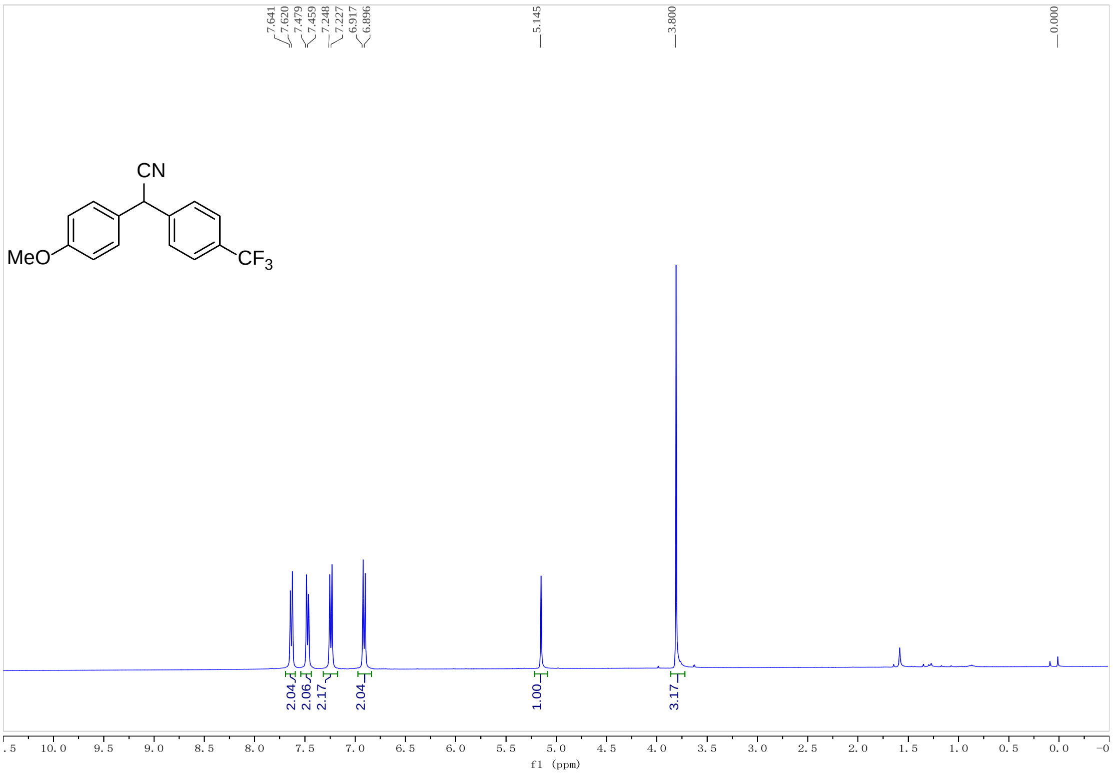

^{13}C NMR (100 MHz, $CDCl_3$)

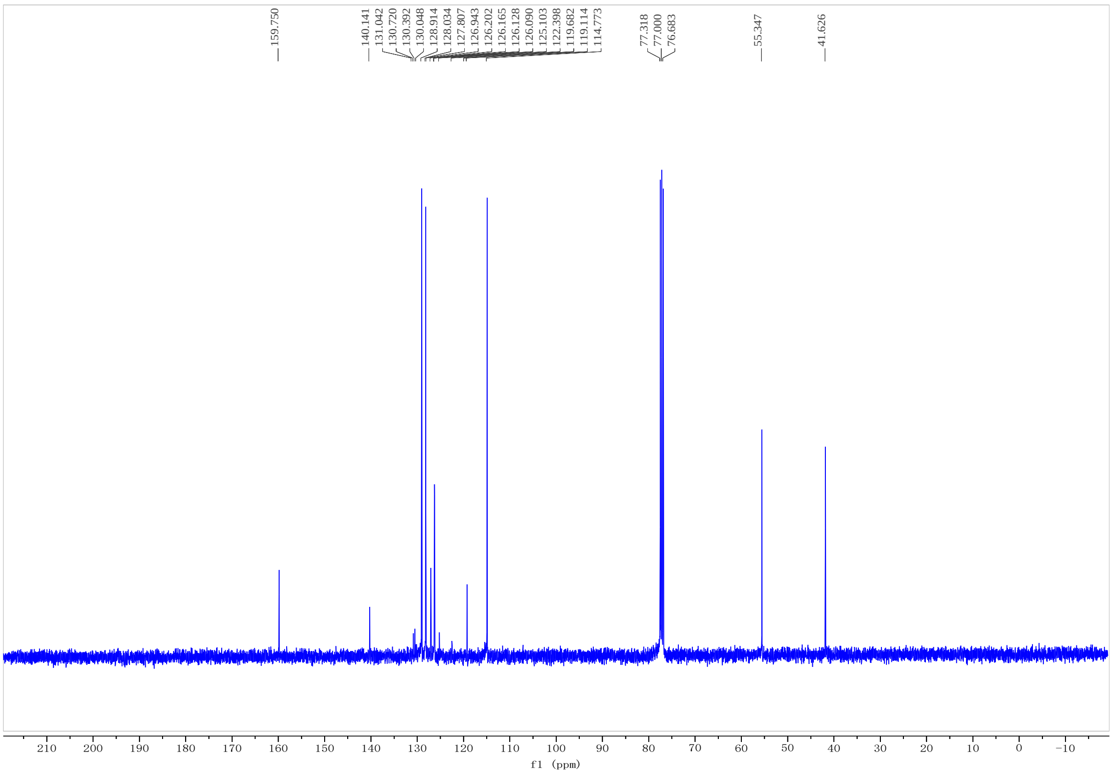

^{19}F NMR (376 MHz, $CDCl_3$)

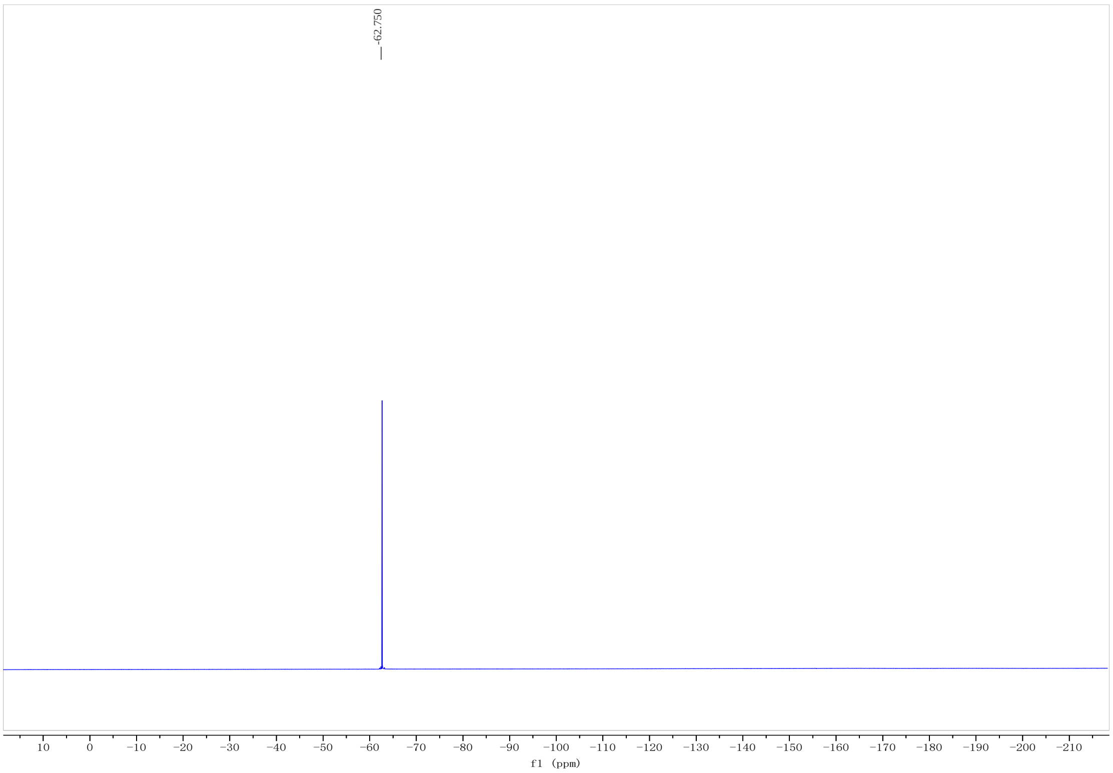

^{1}H and ^{13}C NMR Spectra for Compound 3i:

^{1}H NMR (400 MHz, $CDCl_3$)

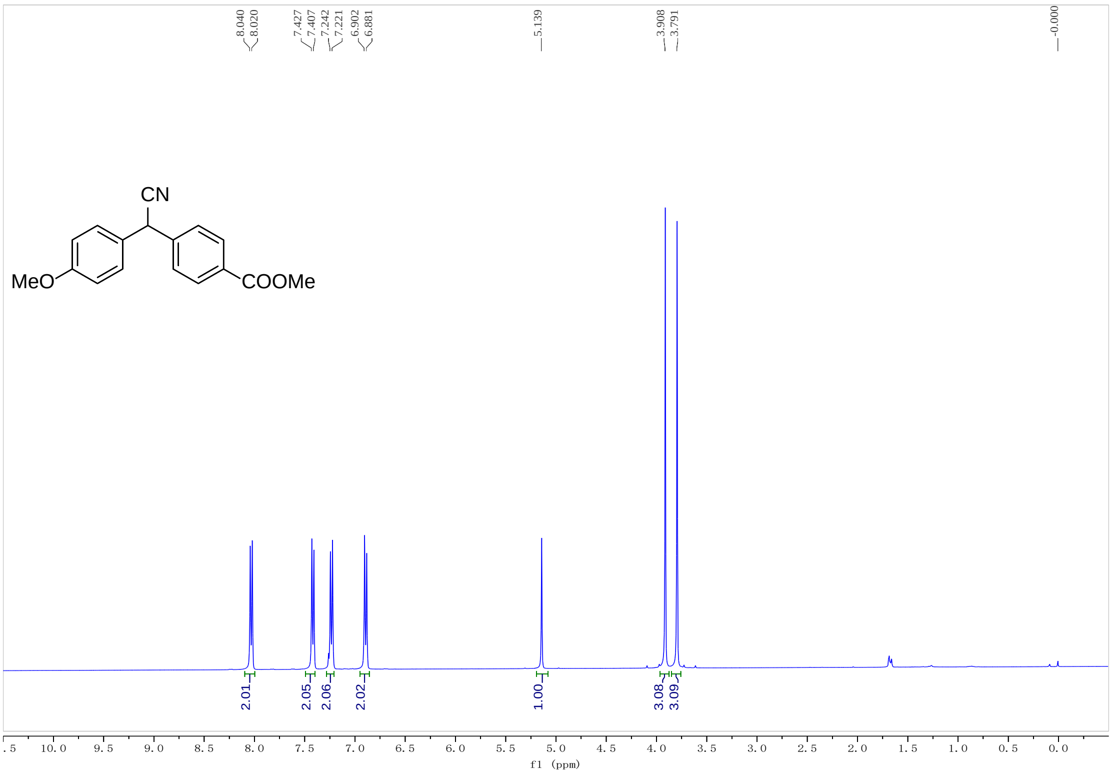

^{13}C NMR (100 MHz, $CDCl_3$)

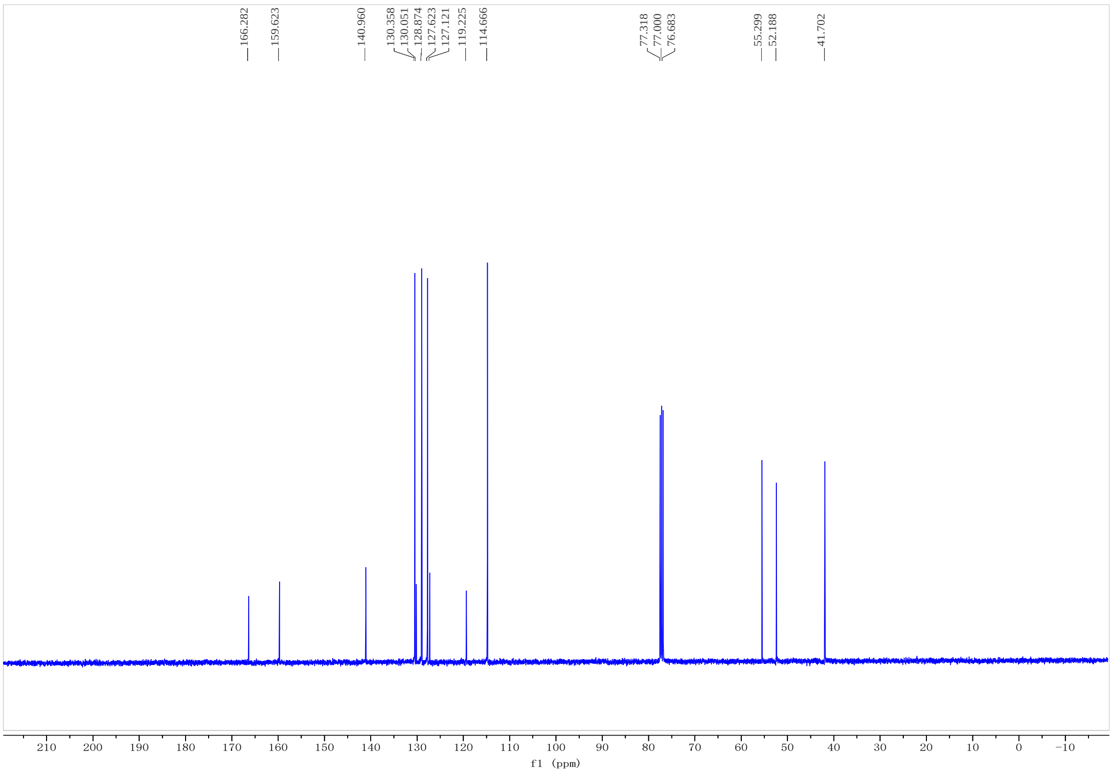

^{1}H and ^{13}C NMR Spectra for Compound 3j:

^{1}H NMR (400 MHz, $CDCl_3$)

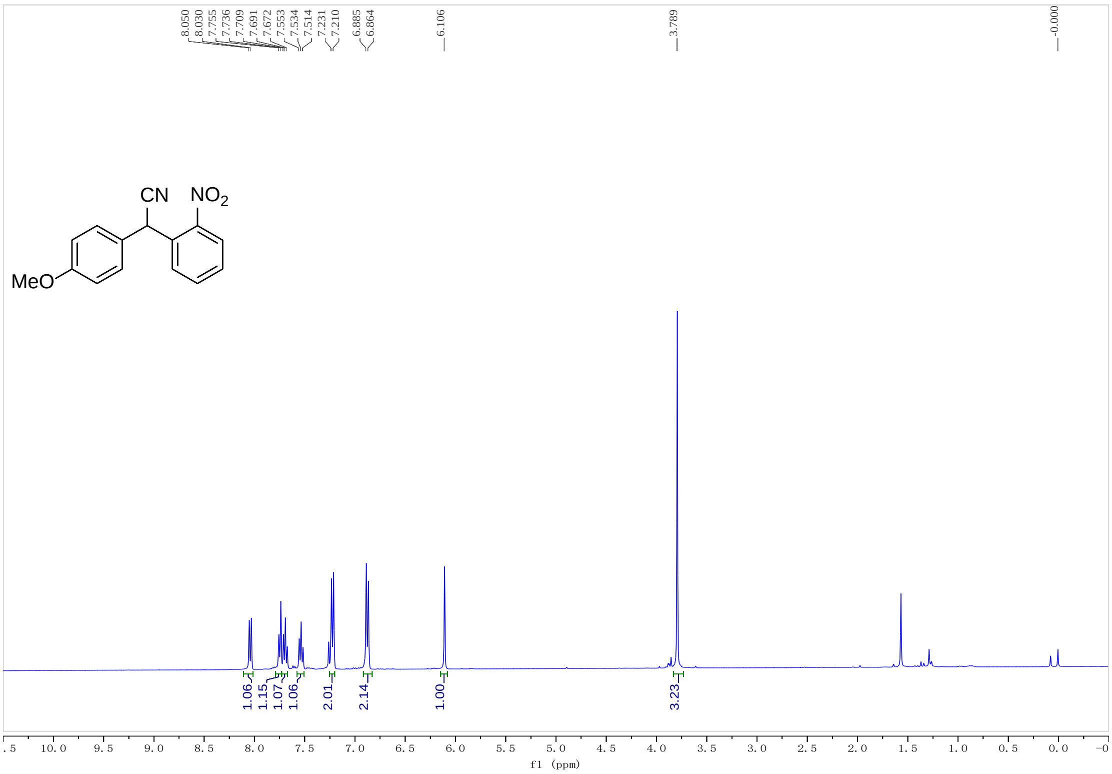

^{13}C NMR (100 MHz, $CDCl_3$)

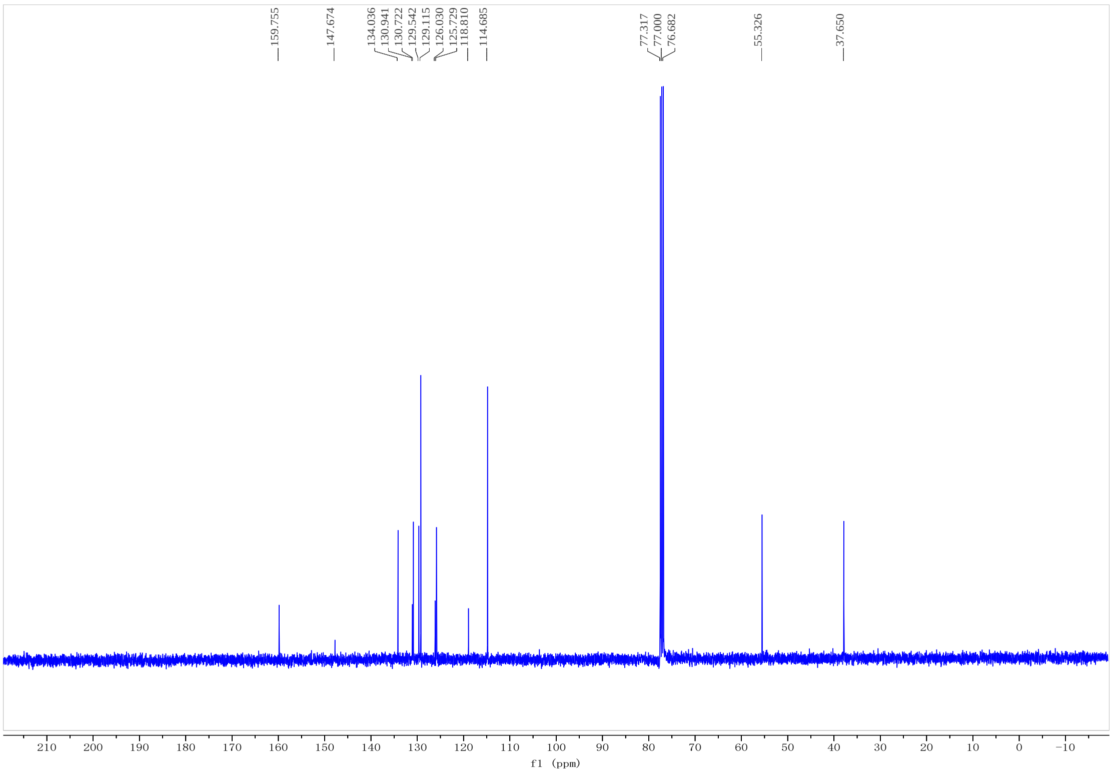

^{1}H and ^{13}C NMR Spectra for Compound 3k:

^{1}H NMR (400 MHz, $CDCl_3$)

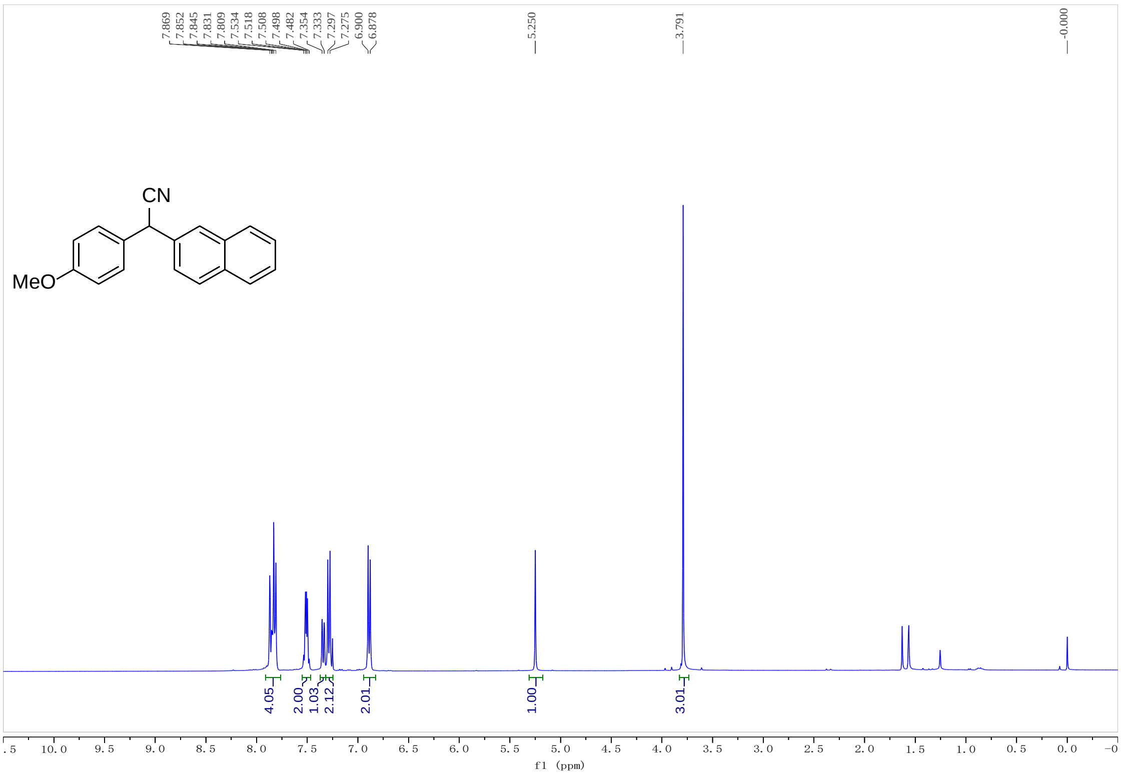

^{13}C NMR (100 MHz, $CDCl_3$)

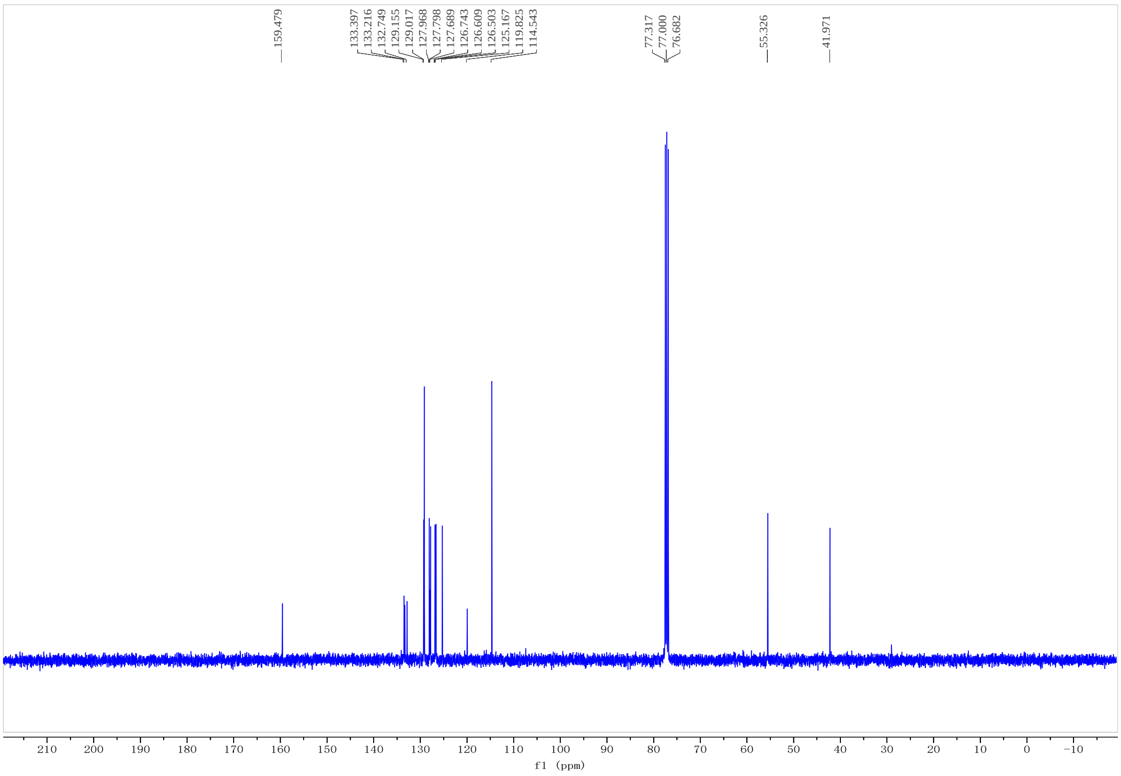

^{1}H and ^{13}C NMR Spectra for Compound 3l:

^{1}H NMR (400 MHz, $CDCl_3$)

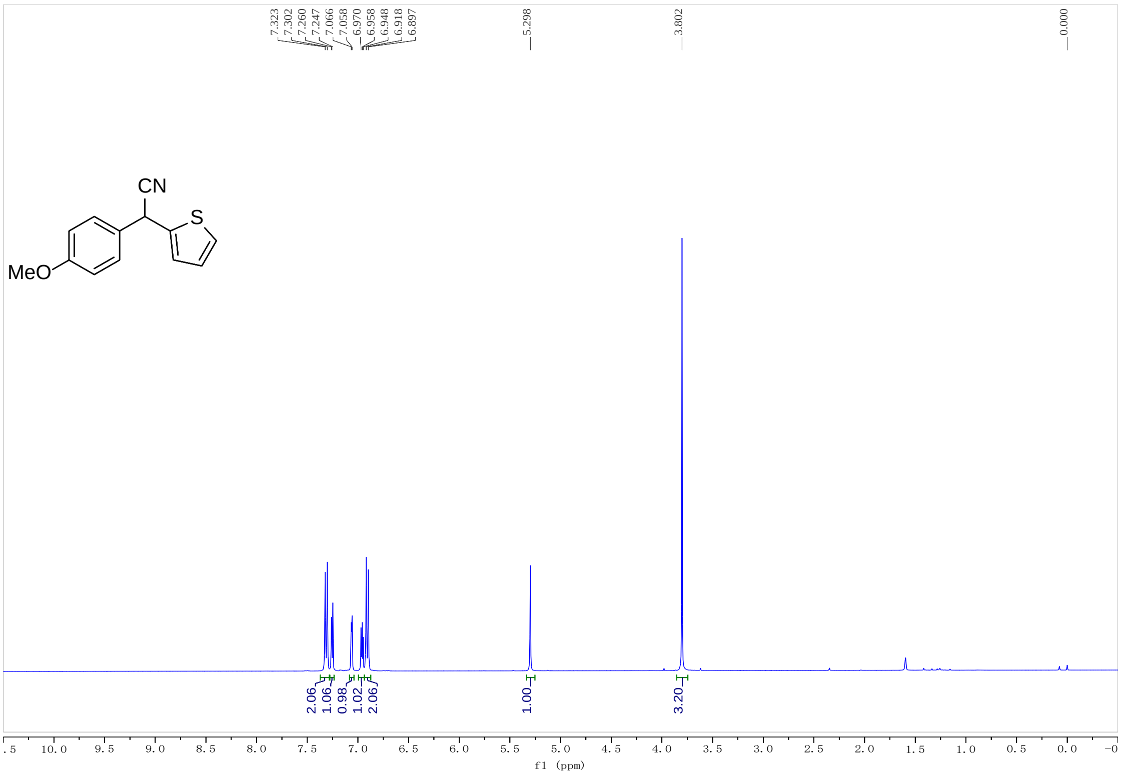

^{13}C NMR (100 MHz, $CDCl_3$)

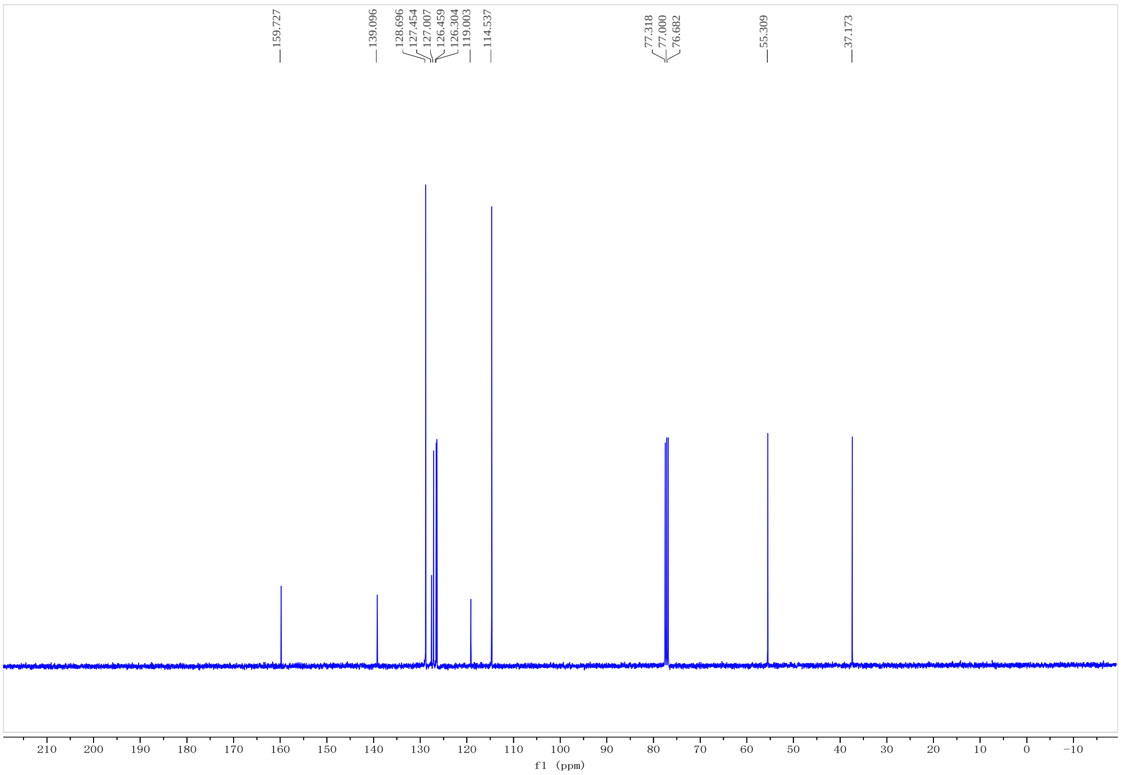

^{1}H and ^{13}C NMR Spectra for Compound 3m:

^{1}H NMR (400 MHz, $CDCl_3$)

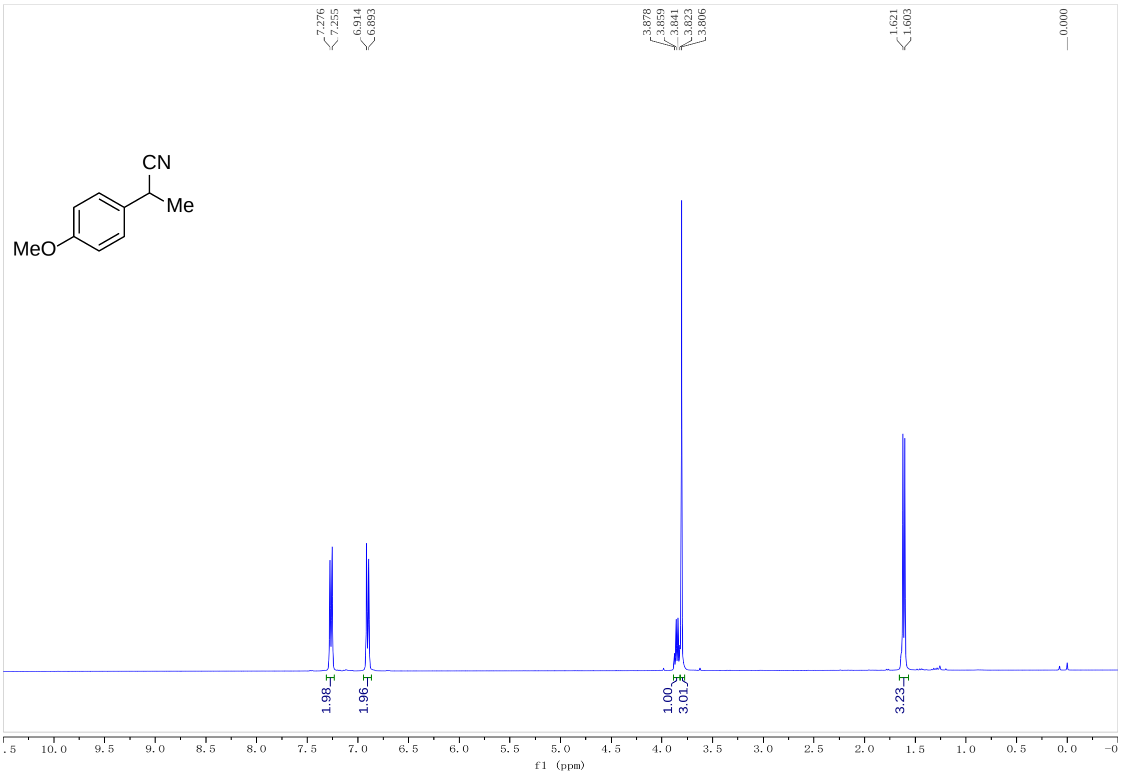

^{13}C NMR (100 MHz, $CDCl_3$)

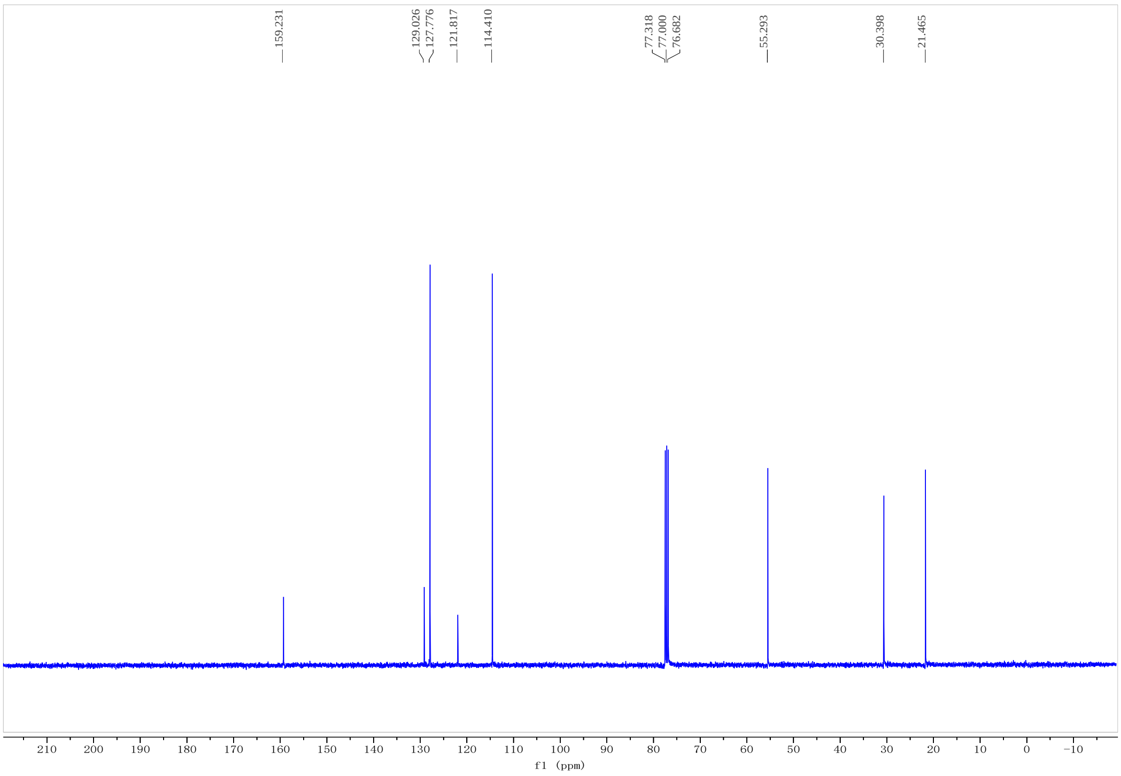

^{1}H and ^{13}C NMR Spectra for Compound 3n:

^{1}H NMR (400 MHz, $CDCl_3$)

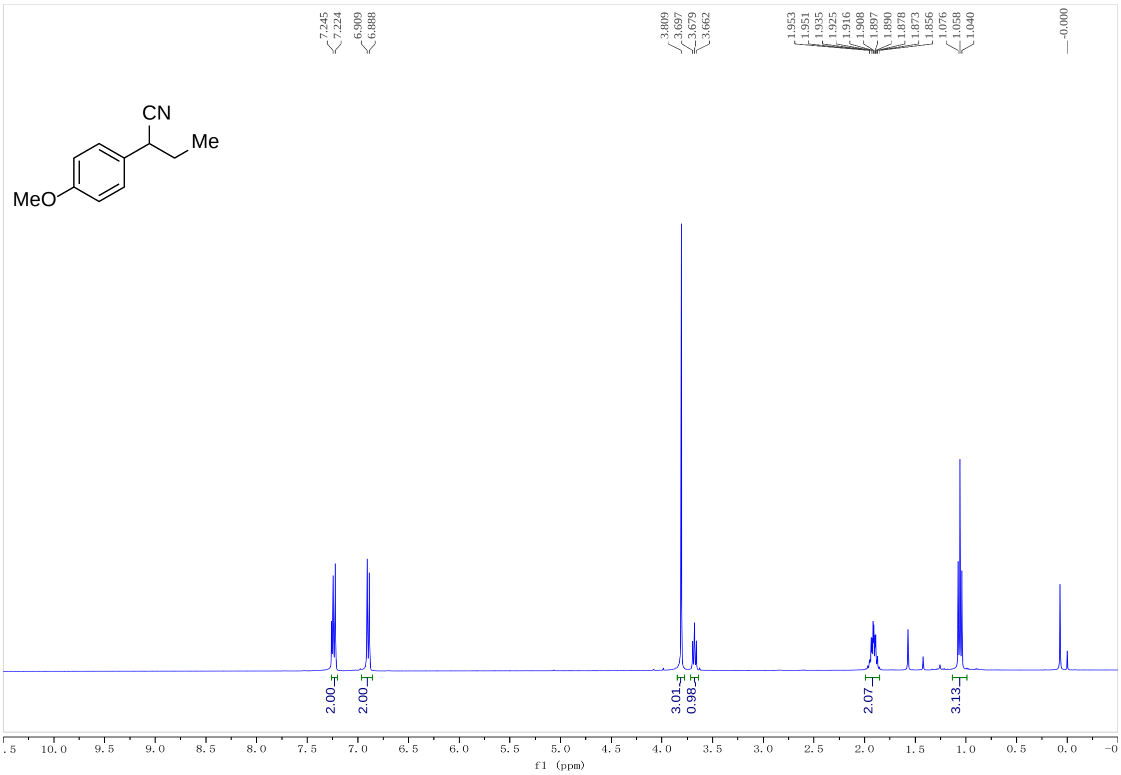

^{13}C NMR (100 MHz, $CDCl_3$)

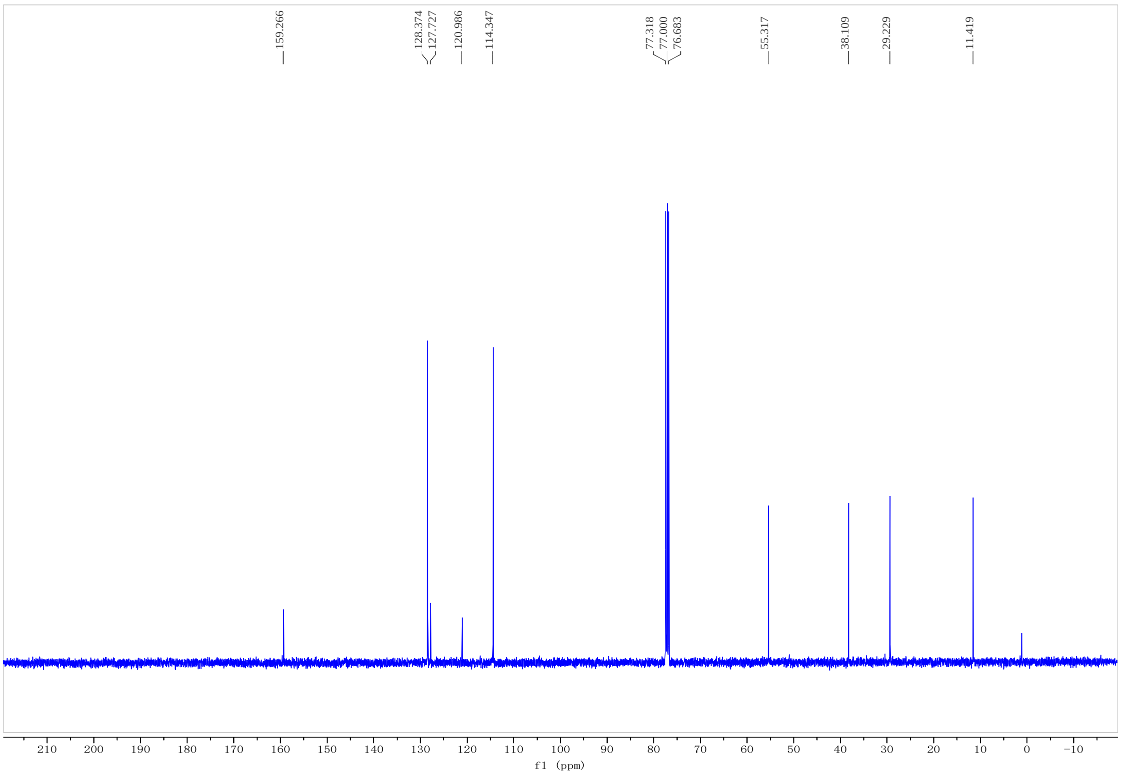

^{1}H and ^{13}C NMR Spectra for Compound 3o:

^{1}H NMR (400 MHz, $CDCl_3$)

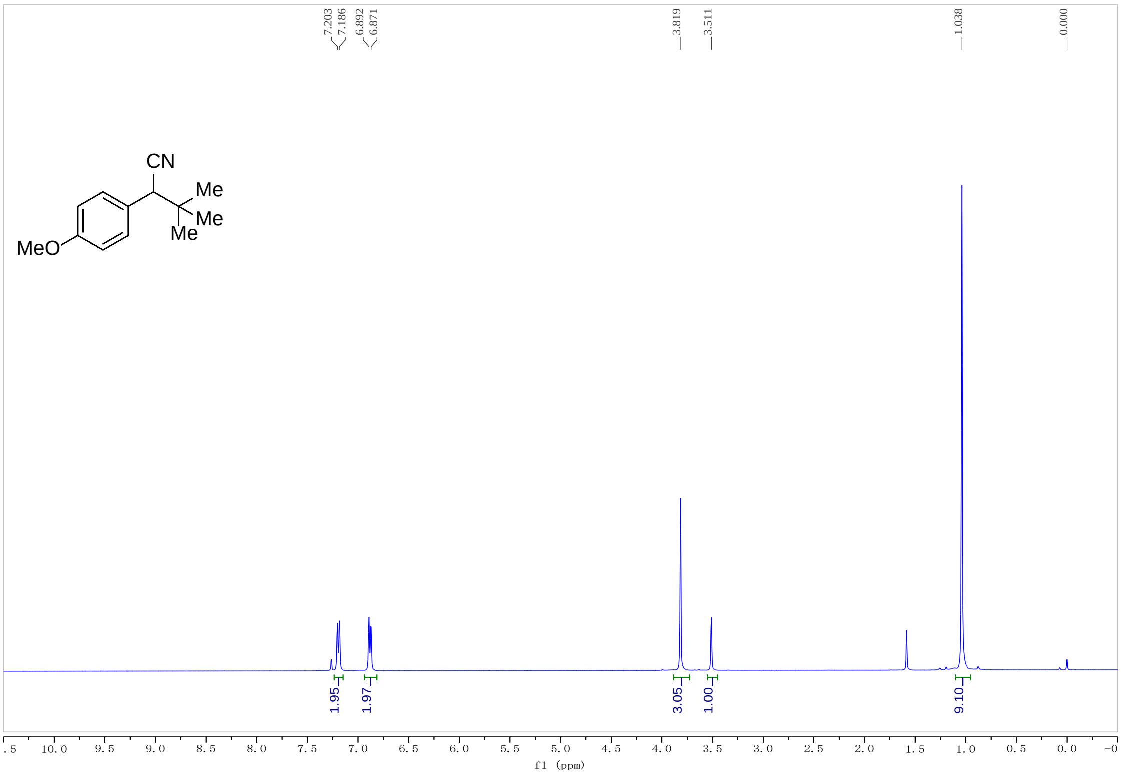

^{13}C NMR (100 MHz, $CDCl_3$)

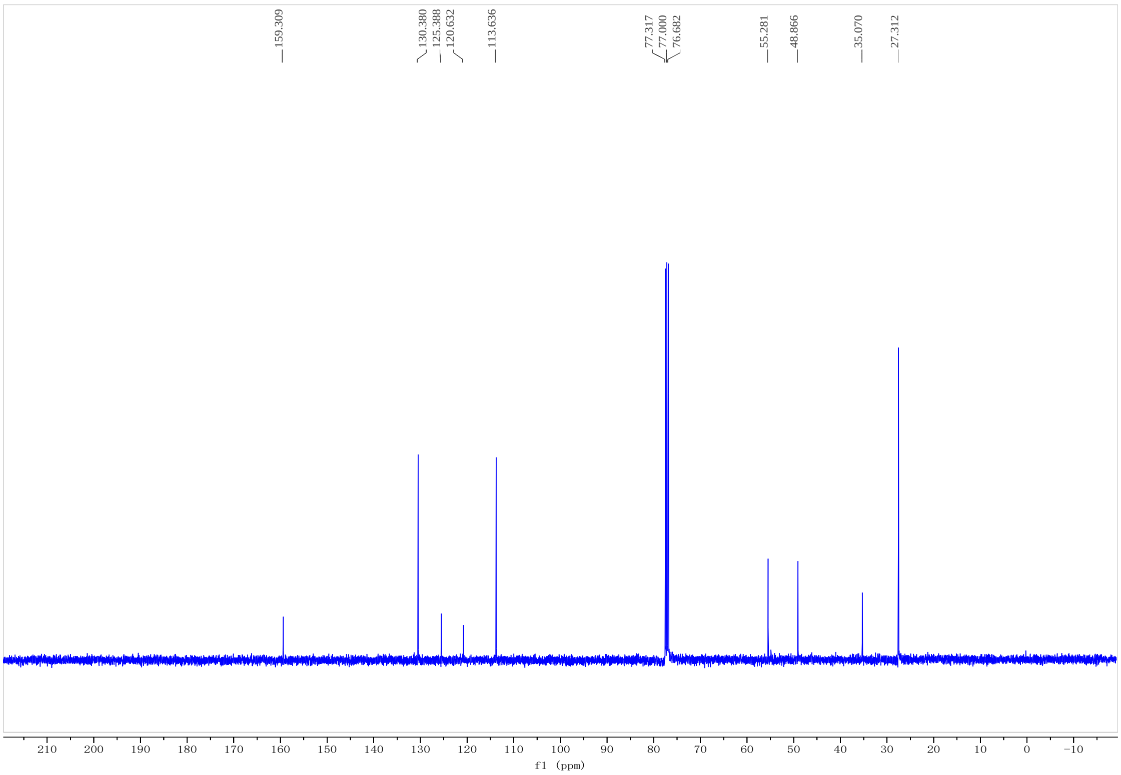

^{1}H and ^{13}C NMR Spectra for Compound 3p:

^{1}H NMR (400 MHz, $CDCl_3$)

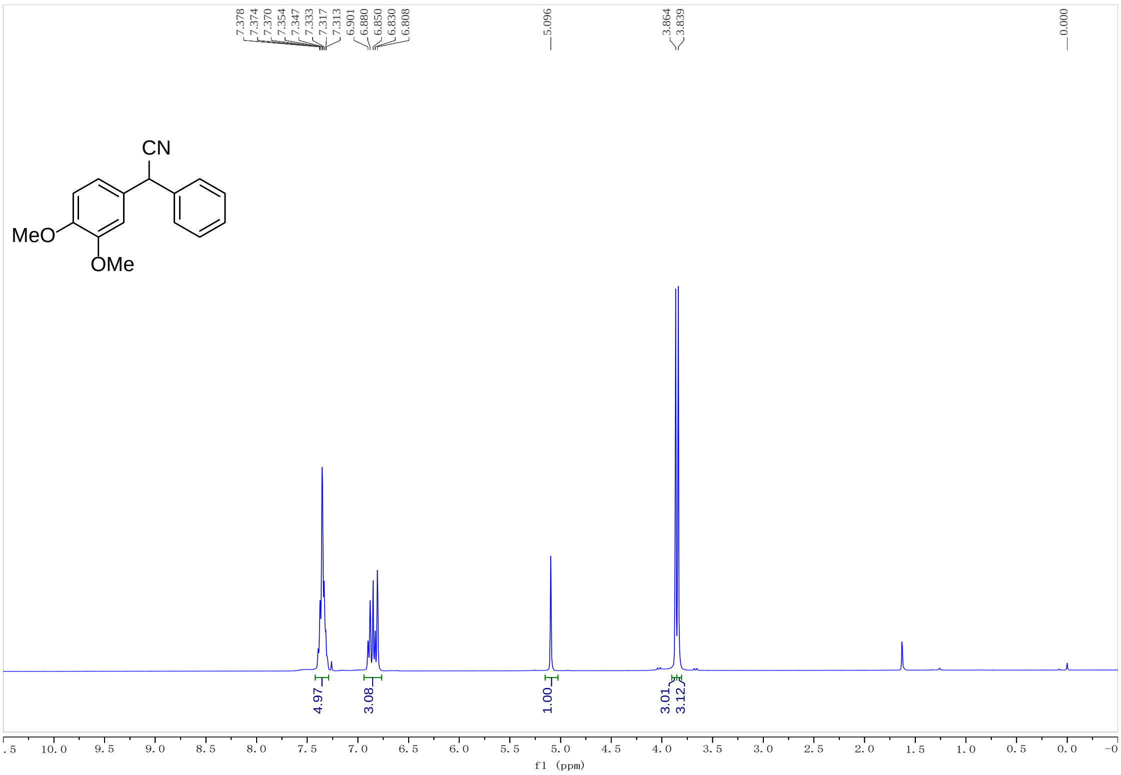

^{13}C NMR (100 MHz, $CDCl_3$)

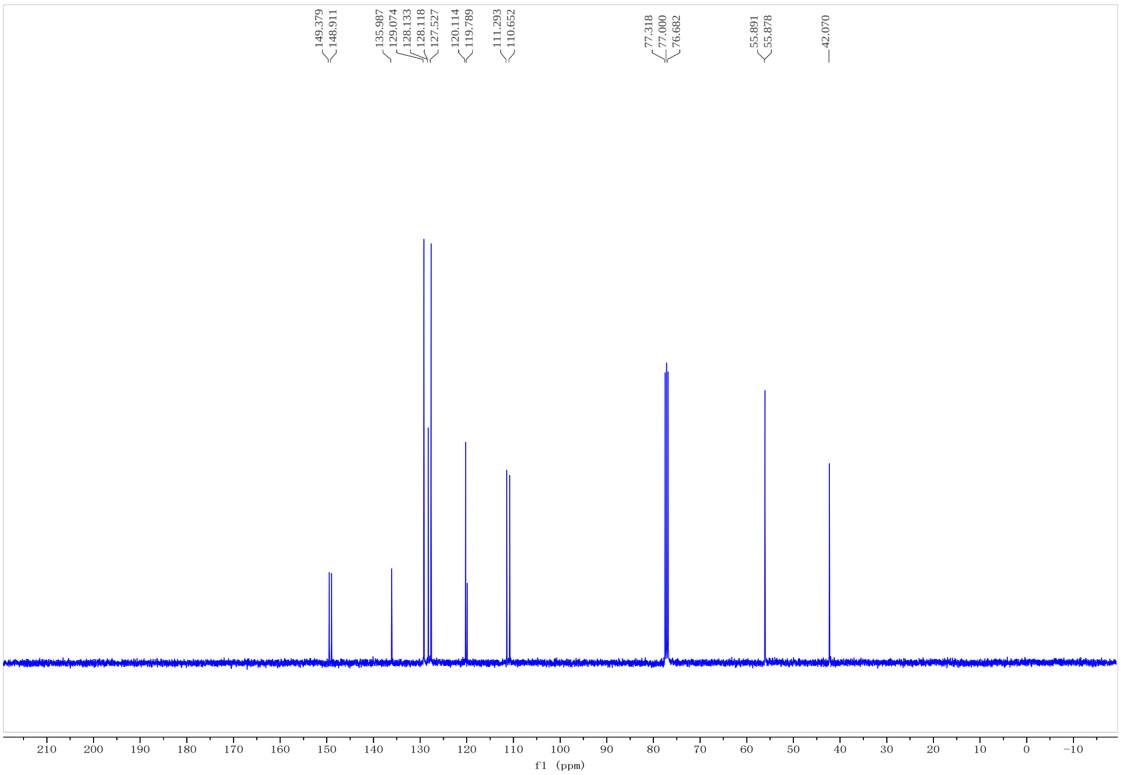

^{1}H and ^{13}C NMR Spectra for Compound 3q:

^{1}H NMR (400 MHz, $CDCl_3$)

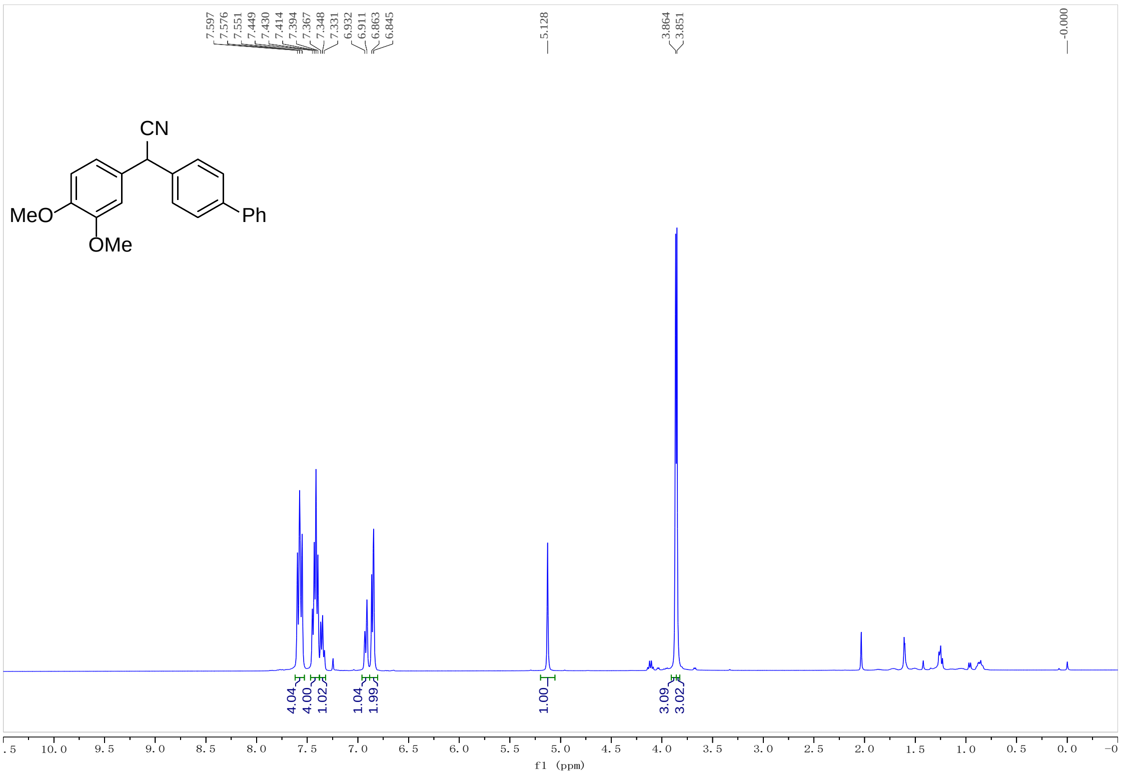

^{13}C NMR (100 MHz, $CDCl_3$)

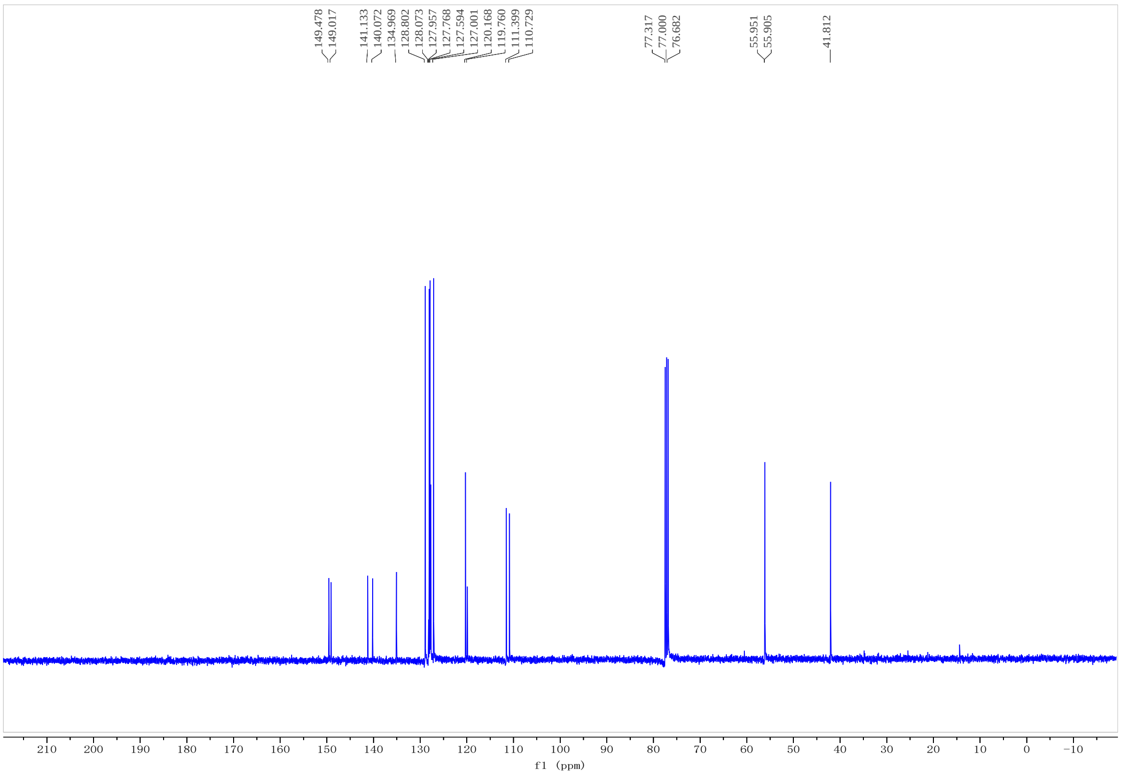

^{1}H and ^{13}C NMR Spectra for Compound 3r:

^{1}H NMR (400 MHz, DMSO-d6)

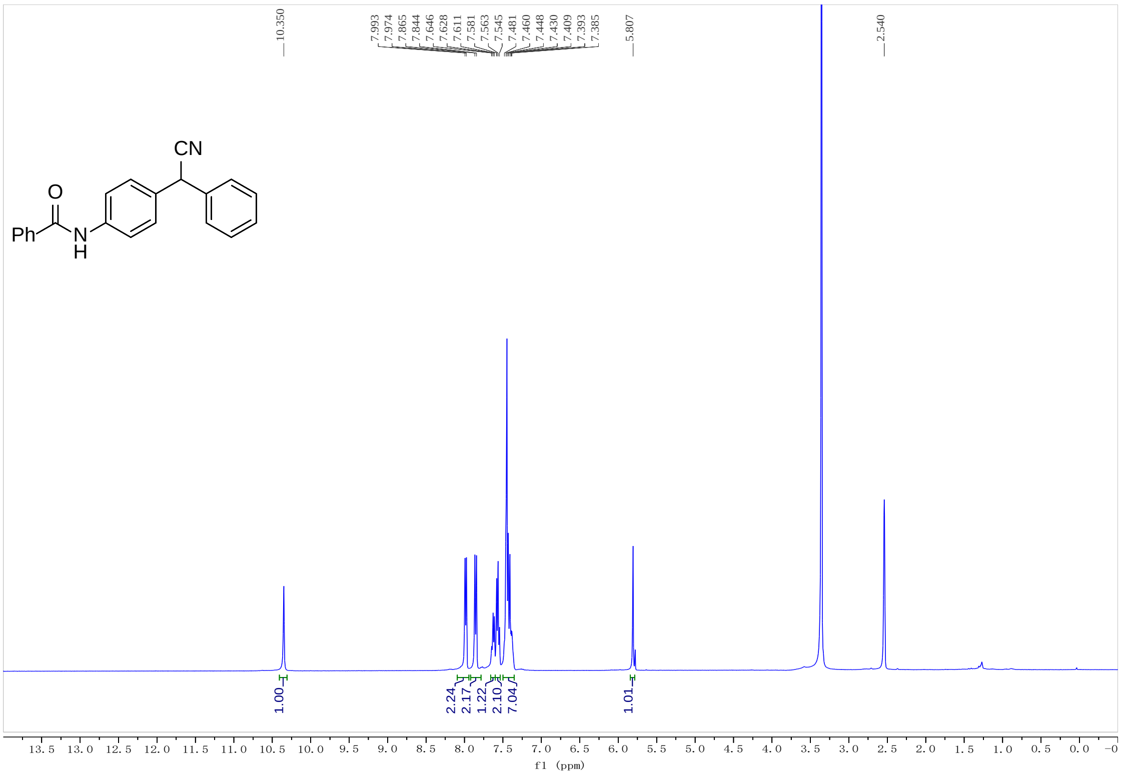

^{13}C NMR (100 MHz, DMSO-d6)

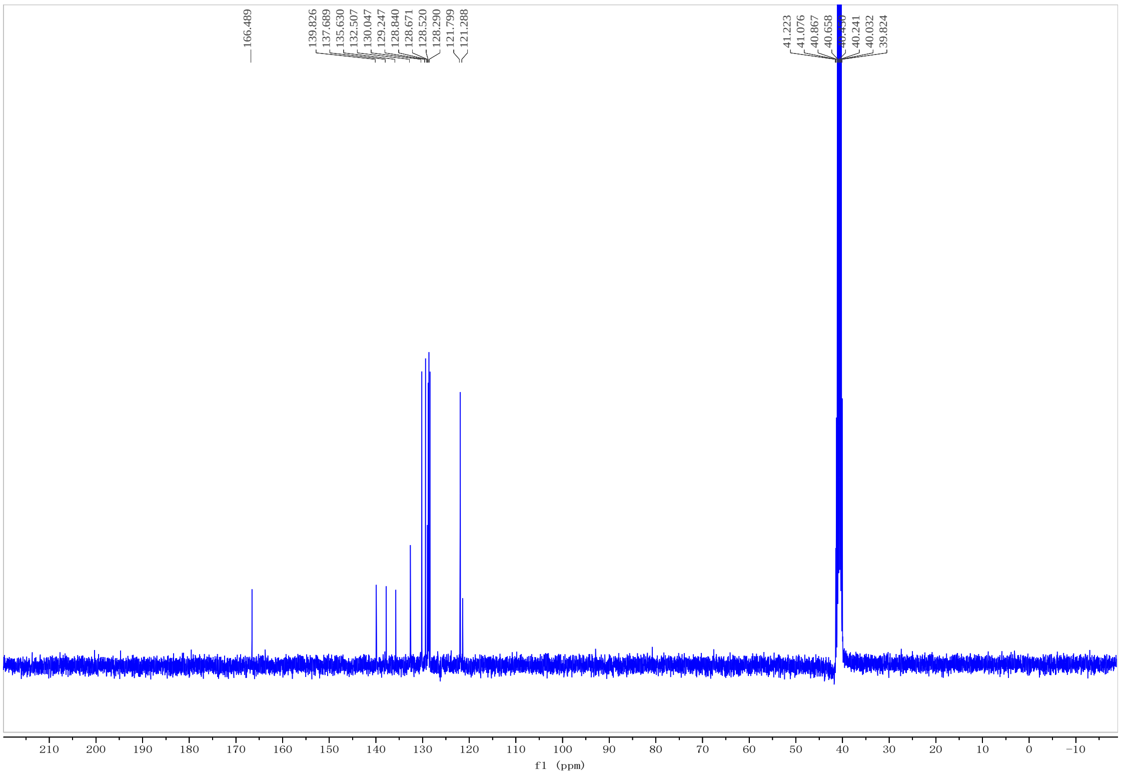

^{1}H and ^{13}C NMR Spectra for Compound 3s:

^{1}H NMR (400 MHz, $CDCl_3$)

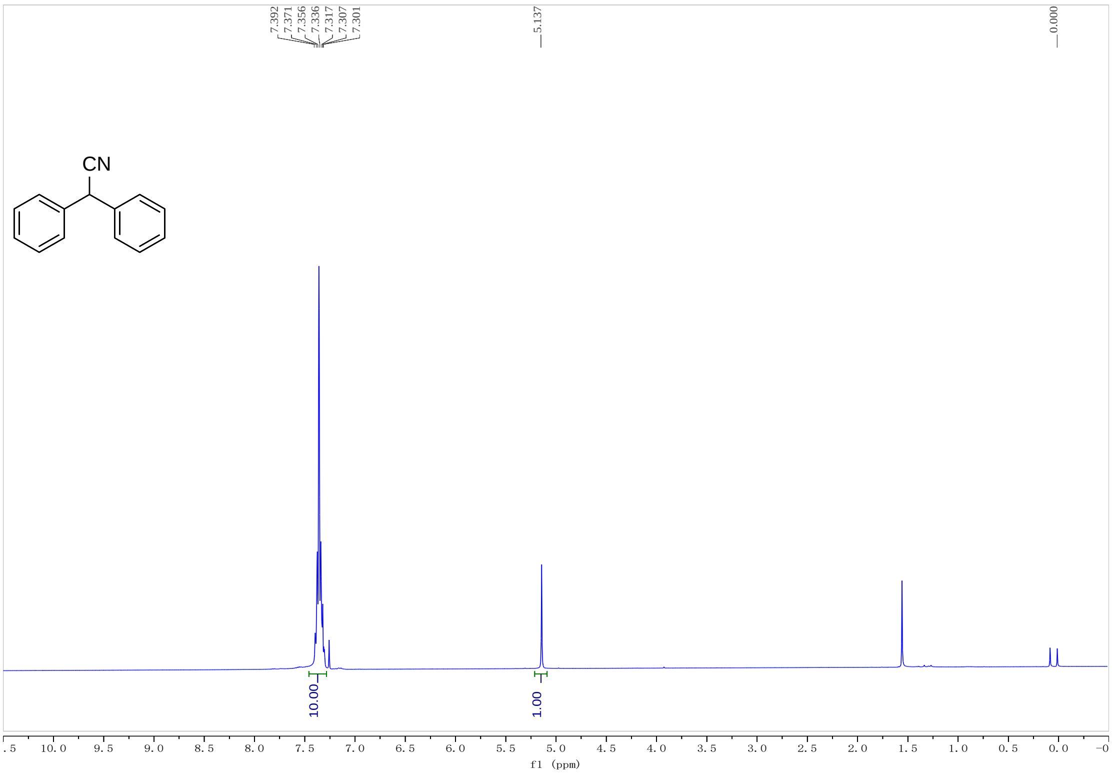

^{13}C NMR (100 MHz, $CDCl_3$)

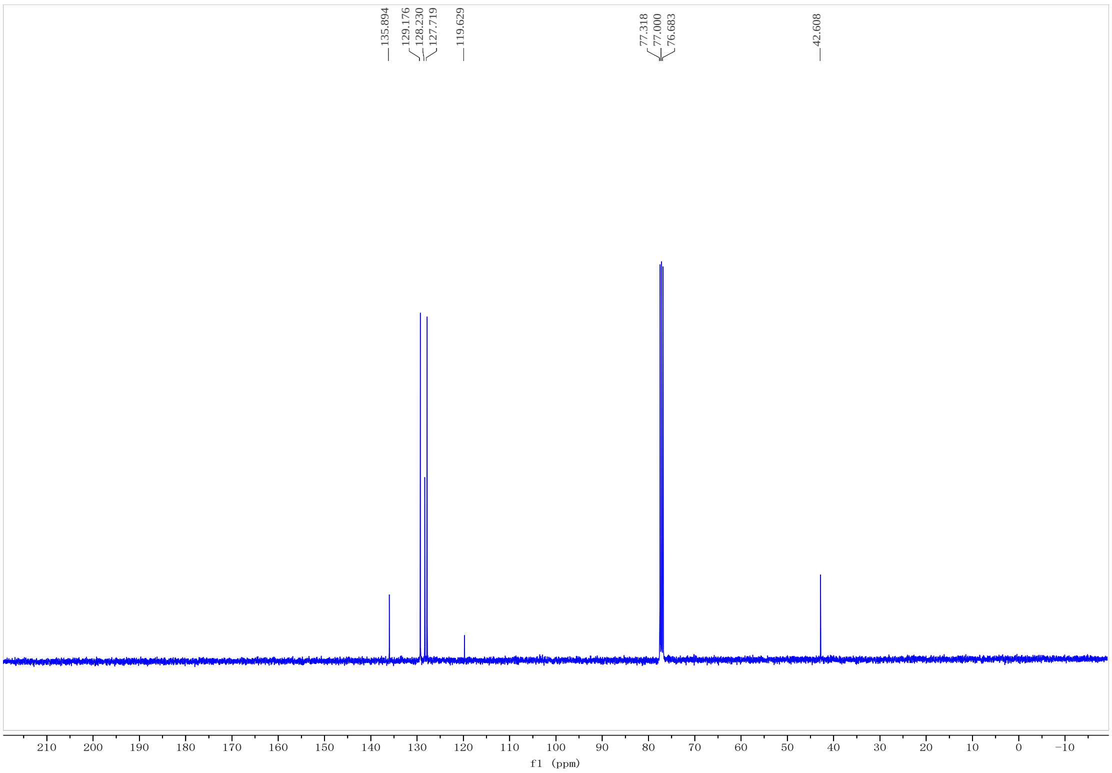

^{1}H and ^{13}C NMR Spectra for Compound 3t:

^{1}H NMR (400 MHz, $CDCl_3$)

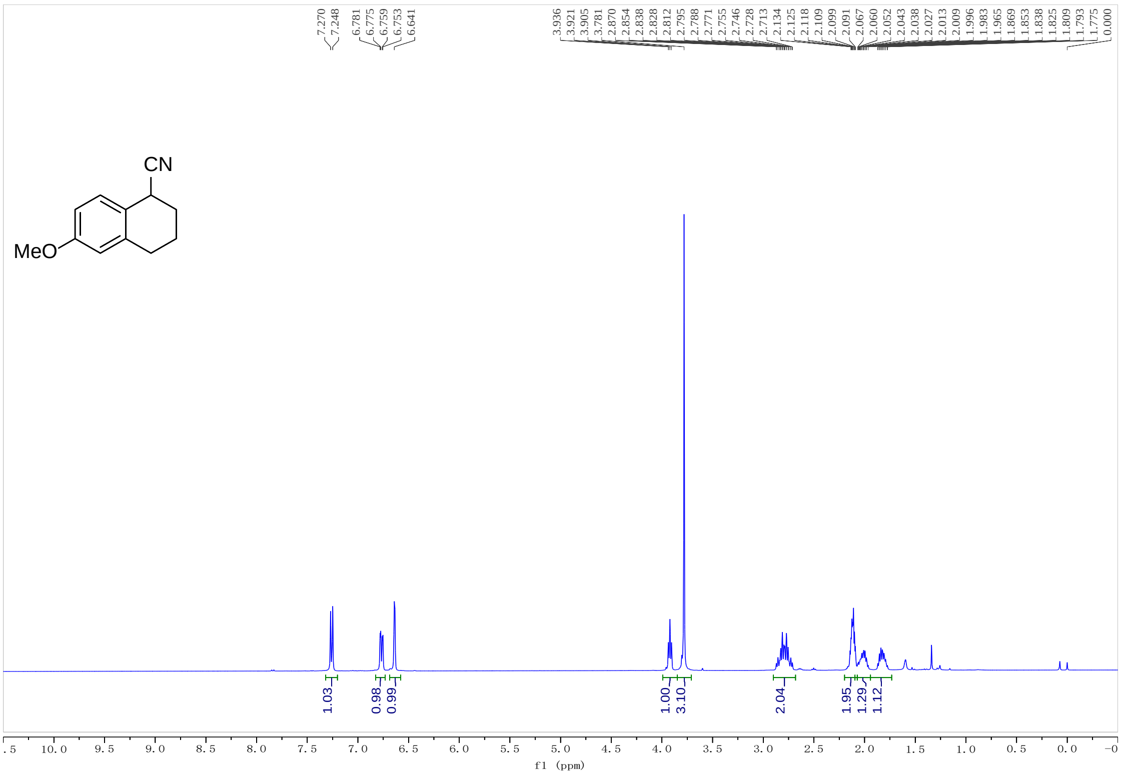

^{13}C NMR (100 MHz, $CDCl_3$)

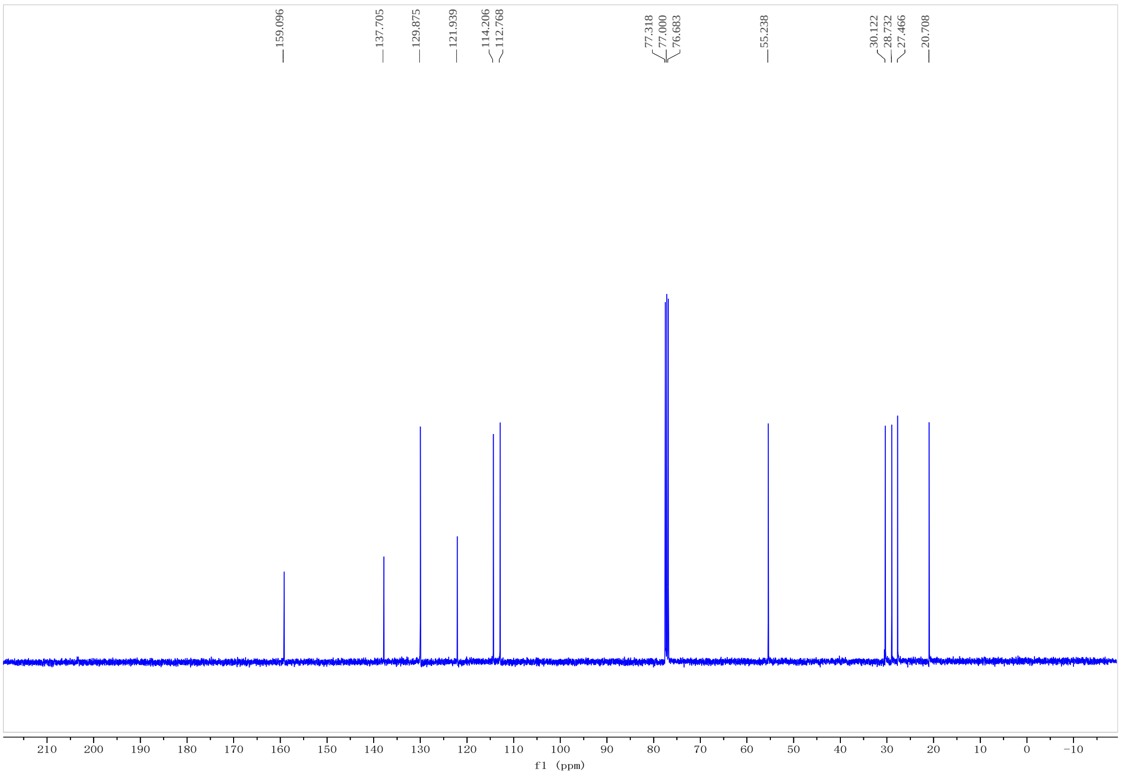

^{1}H and ^{13}C NMR Spectra for Compound 3u:

^{1}H NMR (400 MHz, $CDCl_3$)

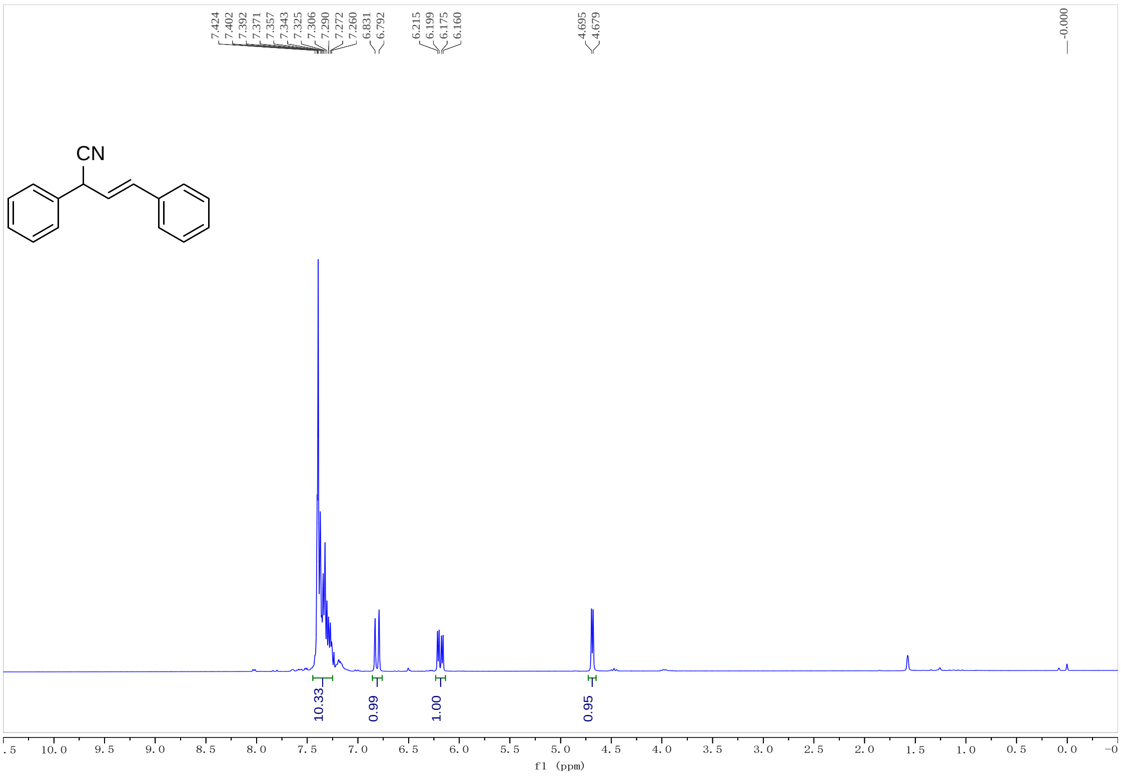

^{13}C NMR (100 MHz, $CDCl_3$)

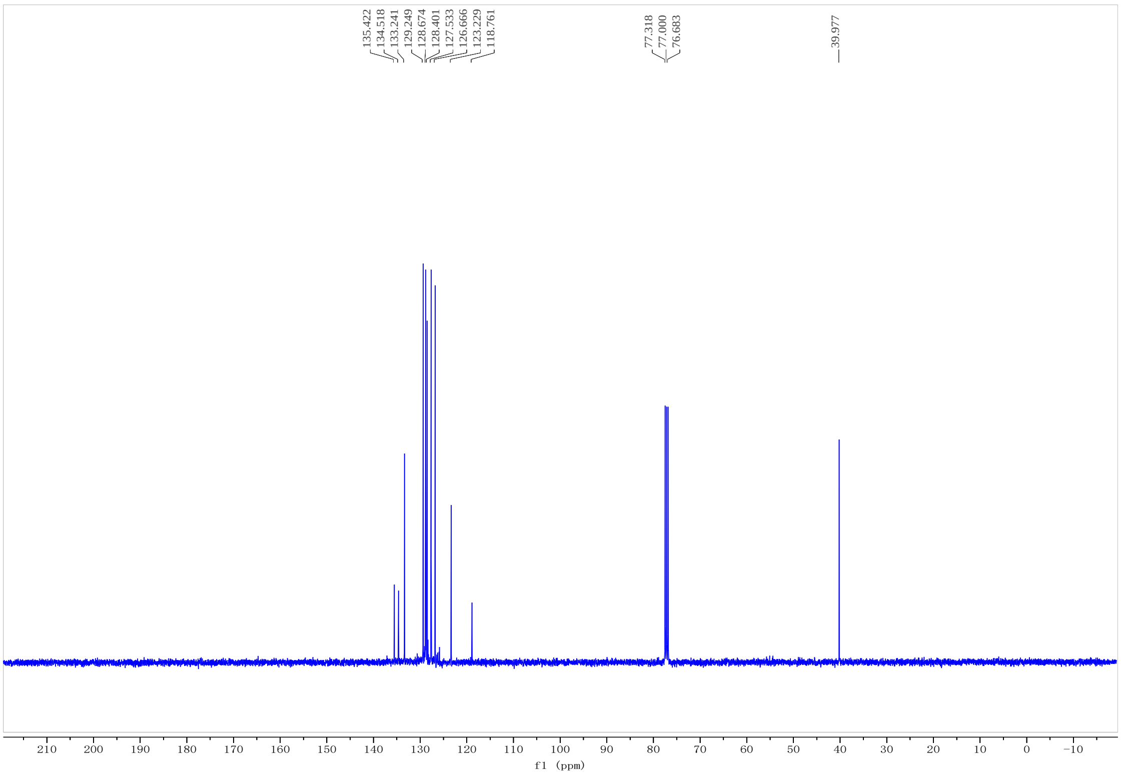

^{1}H and ^{13}C NMR Spectra for Compound 3v:

^{1}H NMR (400 MHz, $CDCl_3$)

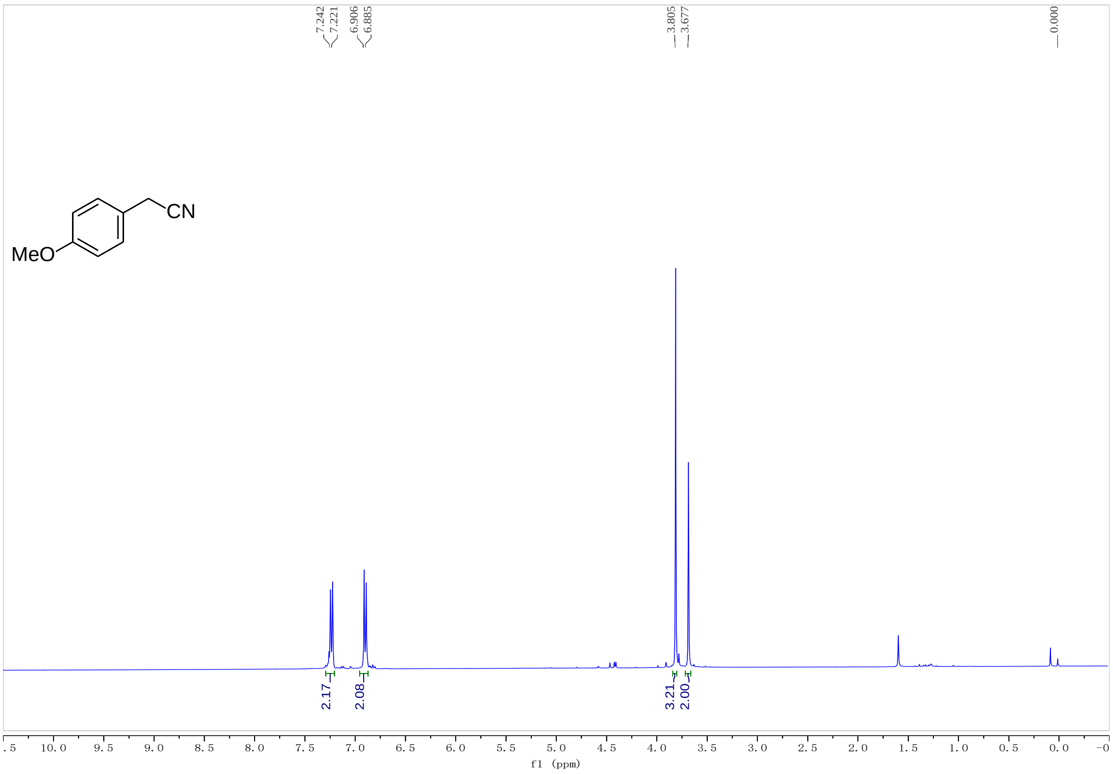

^{13}C NMR (100 MHz, $CDCl_3$)

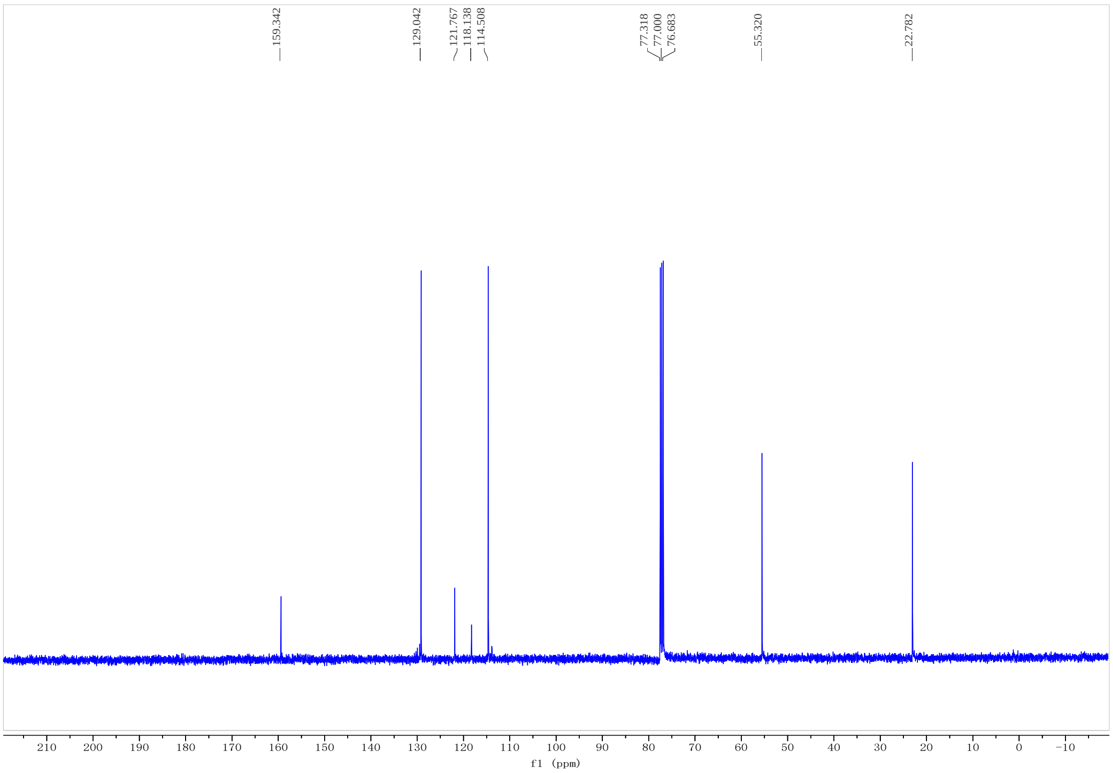

^{1}H and ^{13}C NMR Spectra for Compound 3w:

^{1}H NMR (400 MHz, $CDCl_3$)

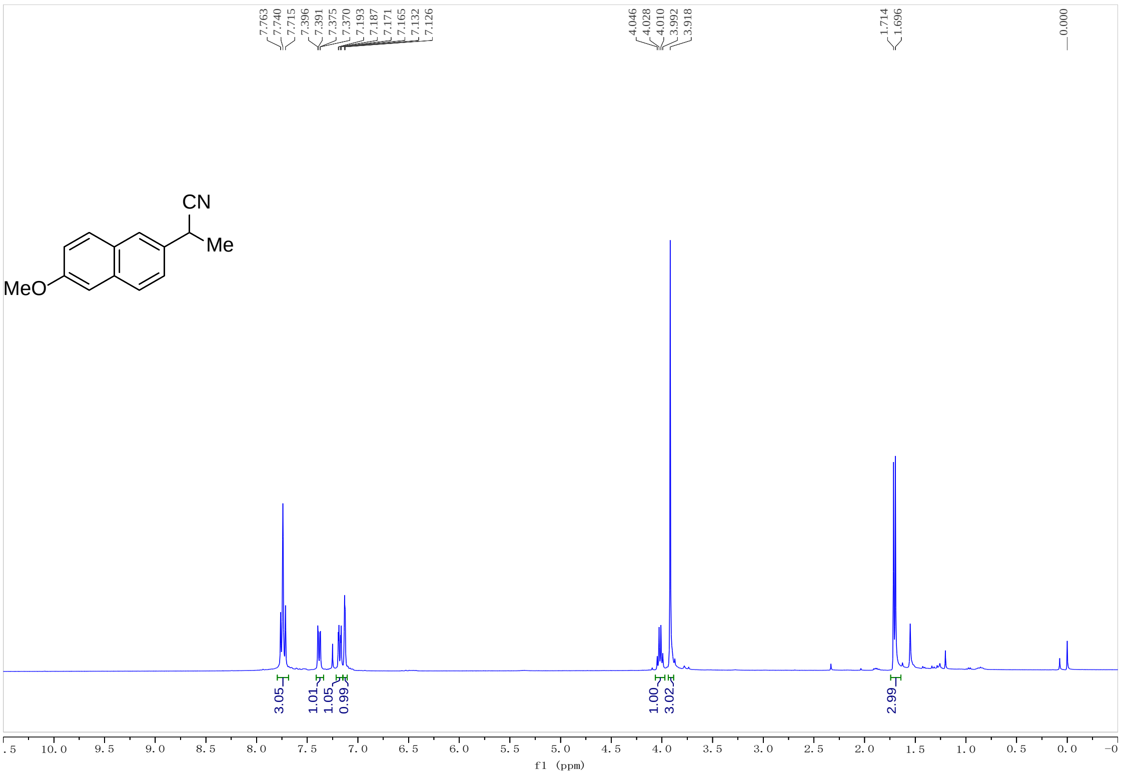

^{13}C NMR (100 MHz, $CDCl_3$)

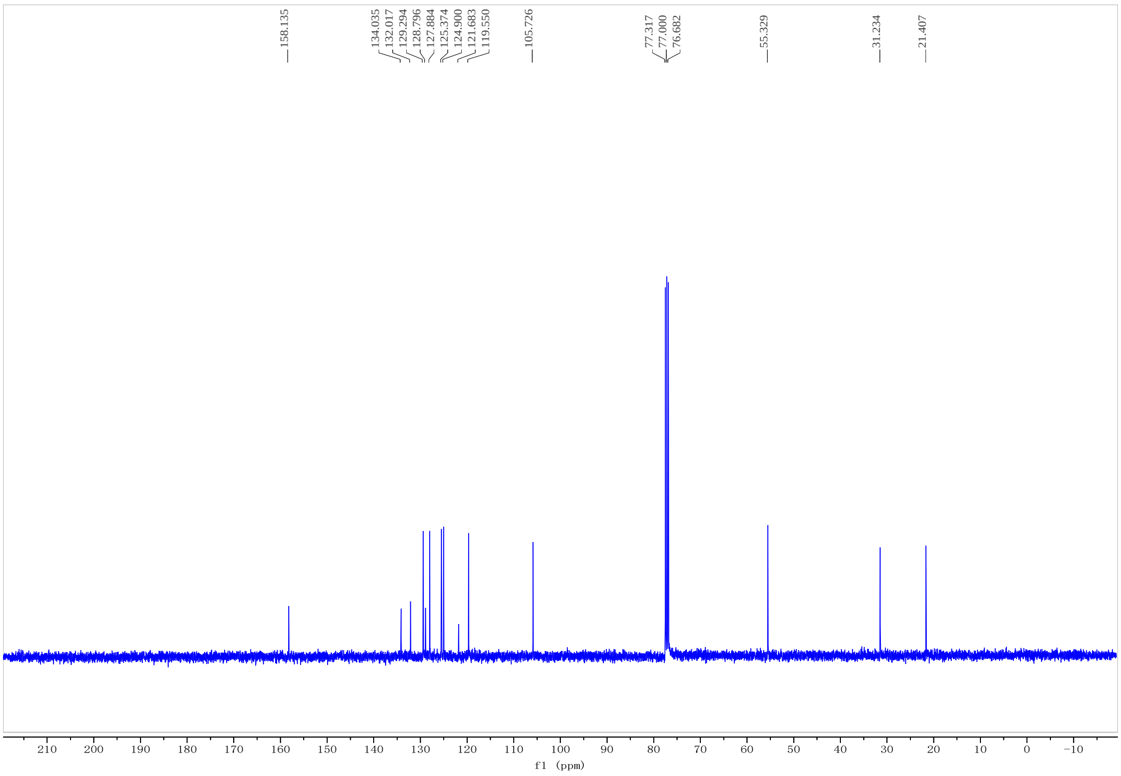

^{1}H and ^{13}C NMR Spectra for Compound 3x:

^{1}H NMR (400 MHz, $CDCl_3$)

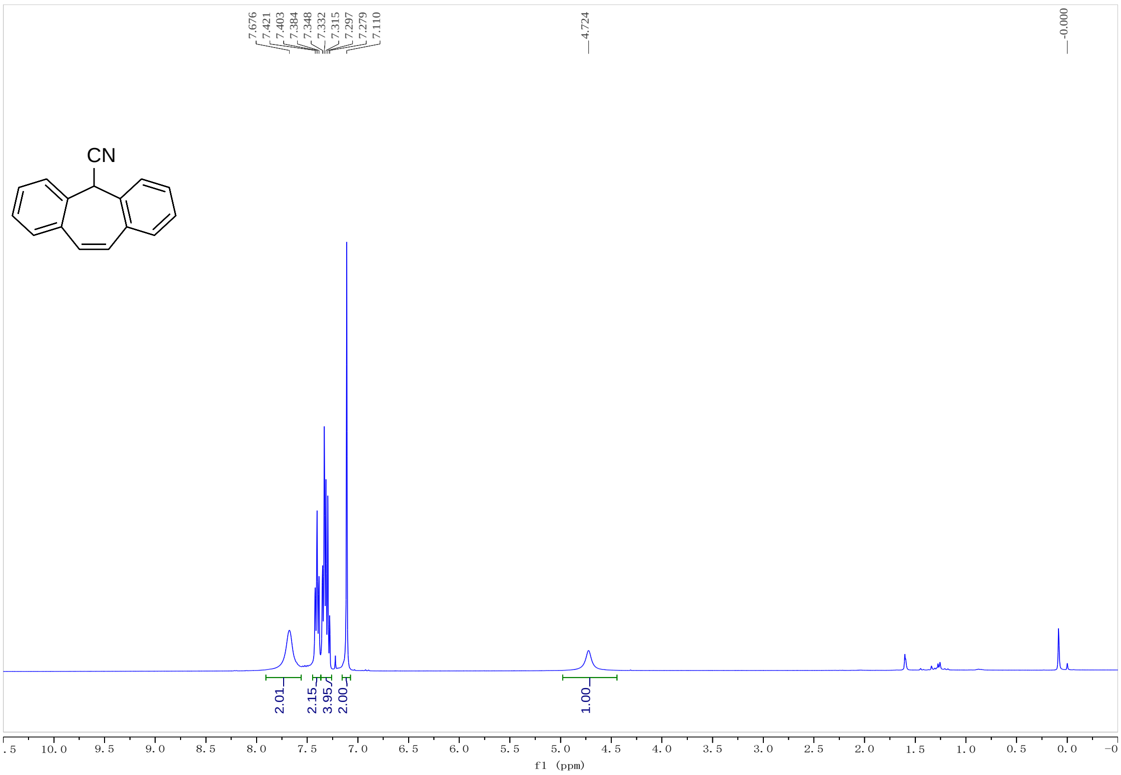

^{13}C NMR (100 MHz, $CDCl_3$)

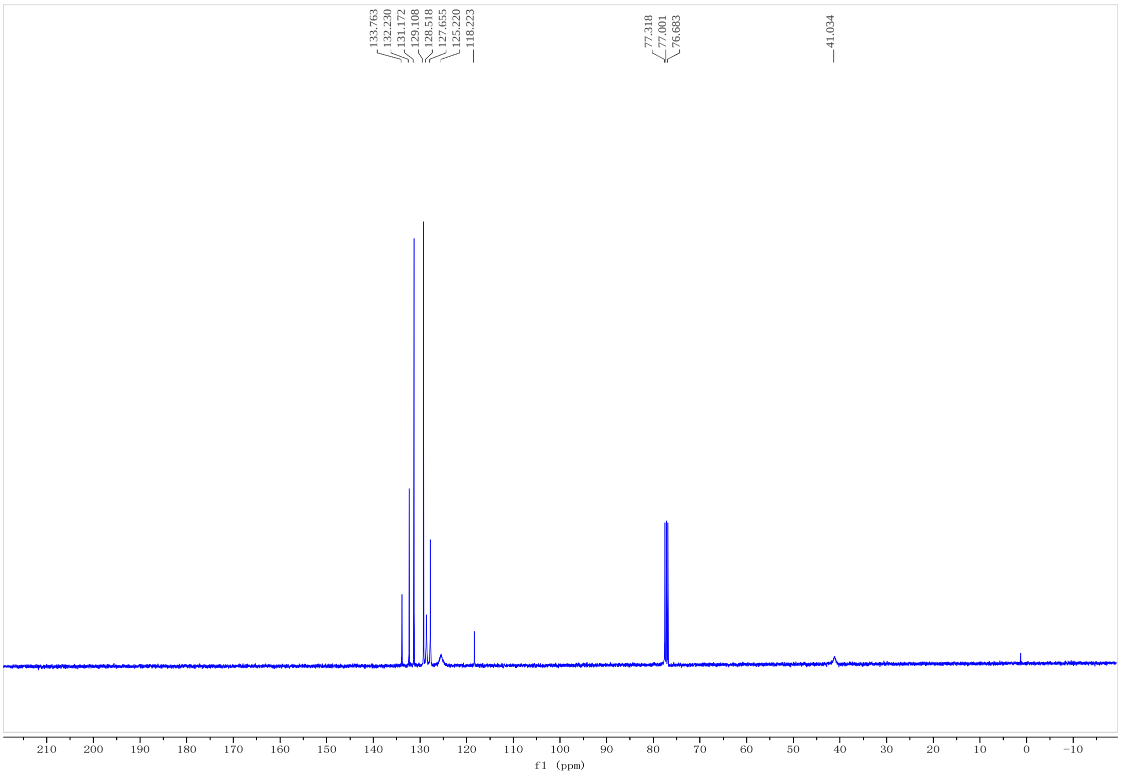

^{1}H and ^{13}C NMR Spectra for Compound 3y:

^{1}H NMR (400 MHz, CDCl$_3$)

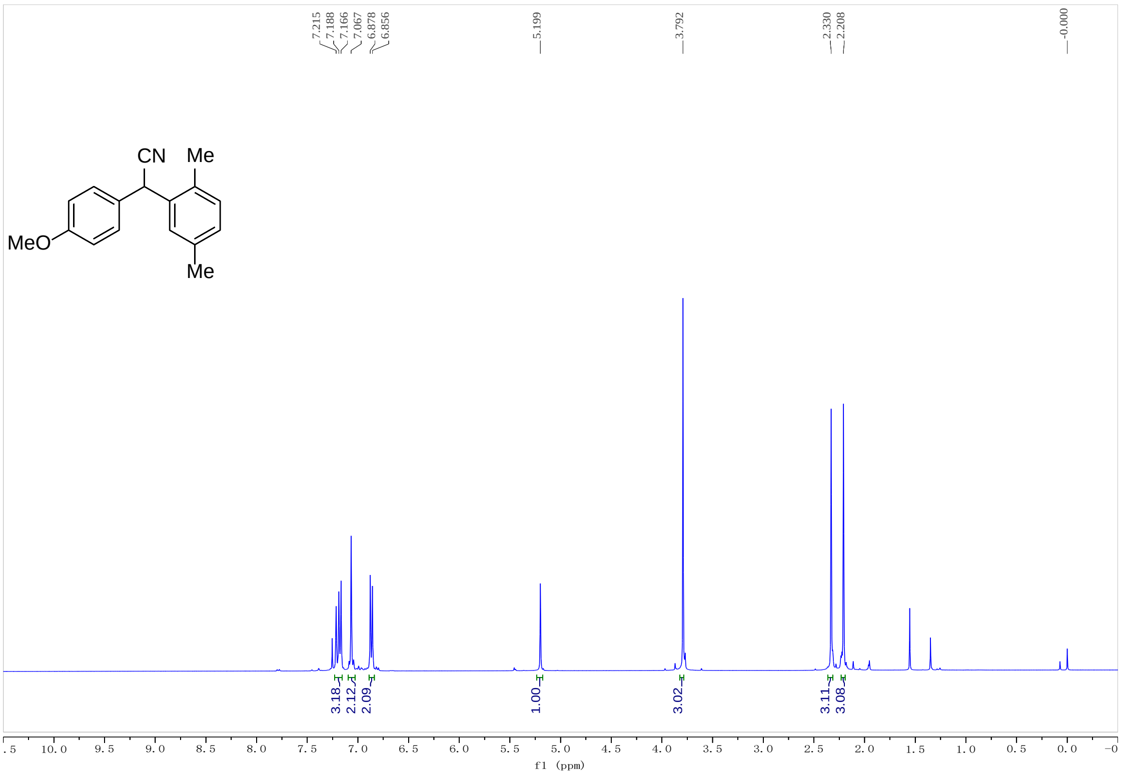

^{13}C NMR (100 MHz, CDCl$_3$)

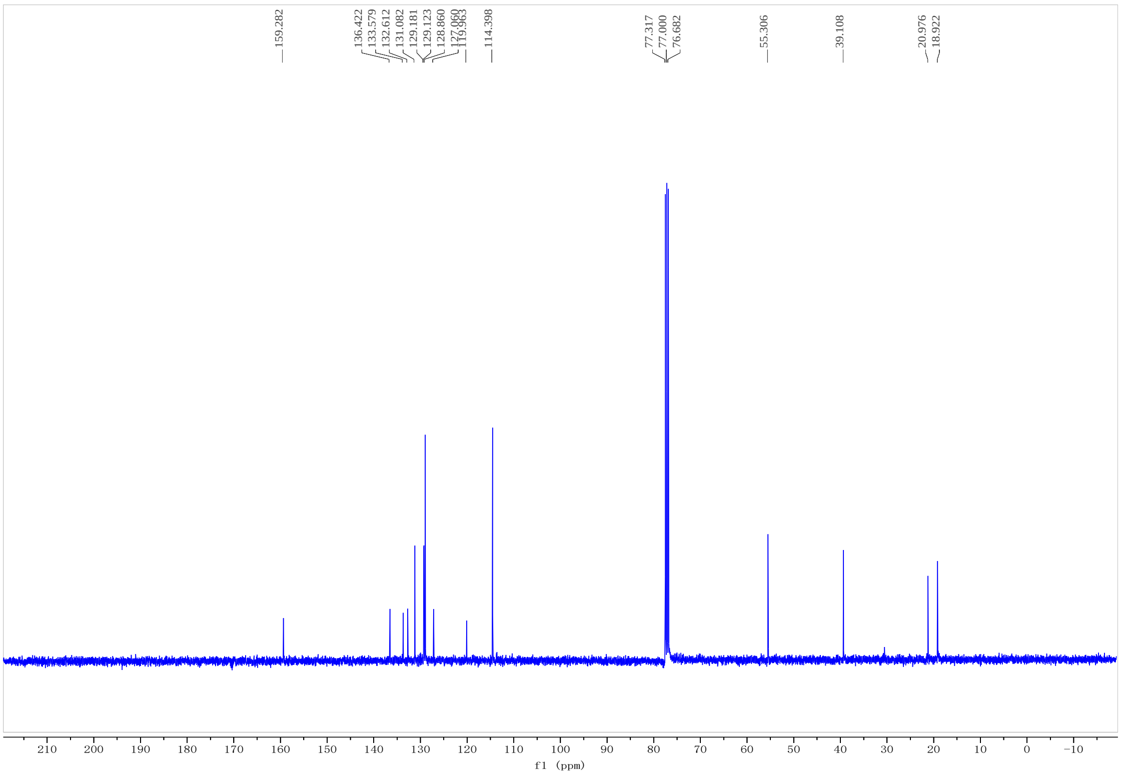

^{1}H and ^{13}C NMR Spectra for Compound 4y:

^{1}H NMR (400 MHz, $CDCl_3$)

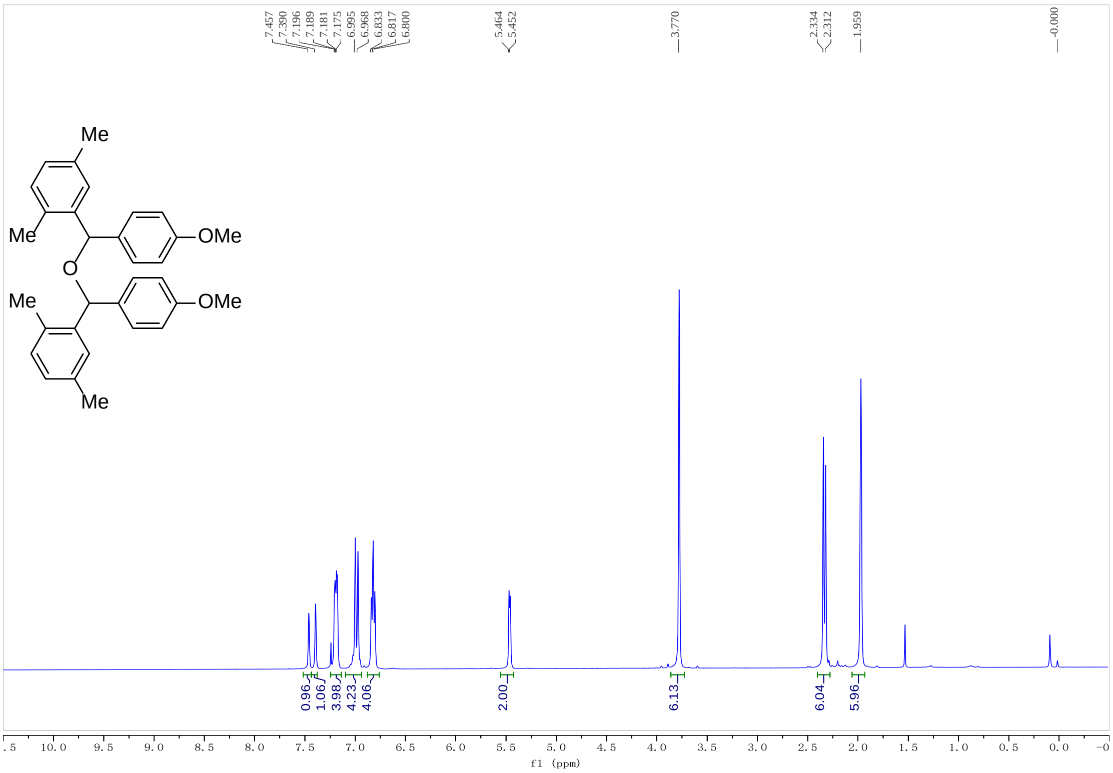

^{13}C NMR (100 MHz, $CDCl_3$)

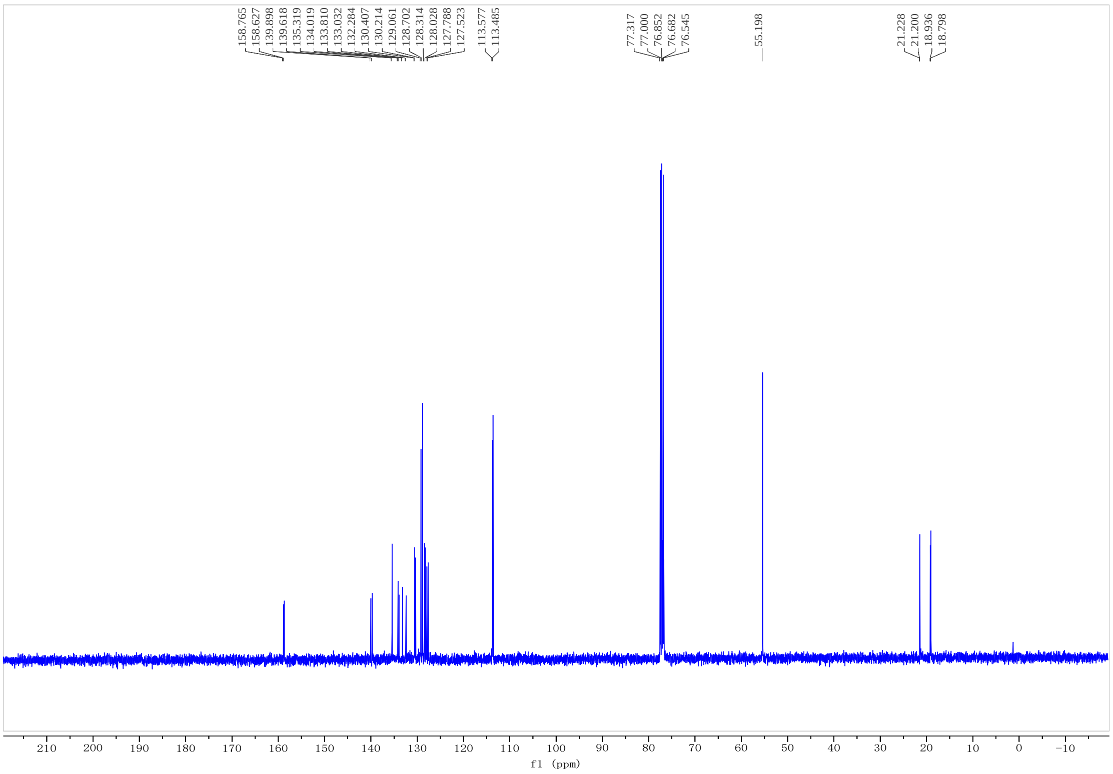

^{1}H and ^{13}C NMR Spectra for Naproxen:

^{1}H NMR (400 MHz, $CDCl_3$)

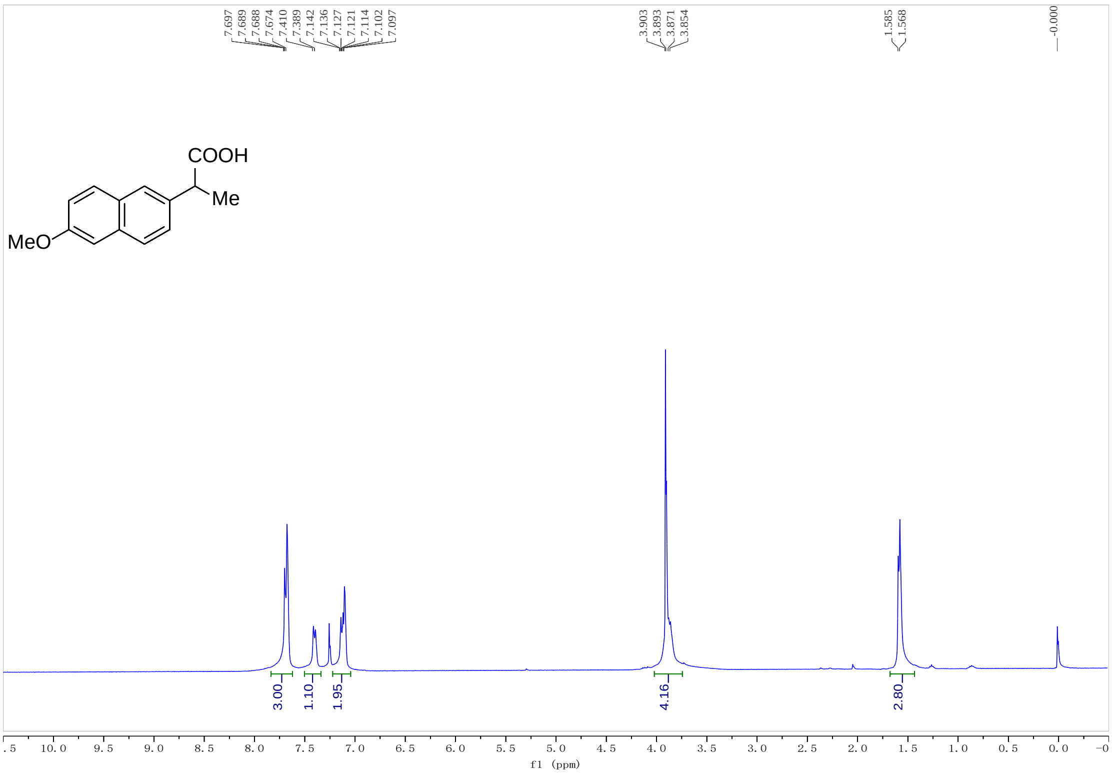

^{13}C NMR (100 MHz, $CDCl_3$)

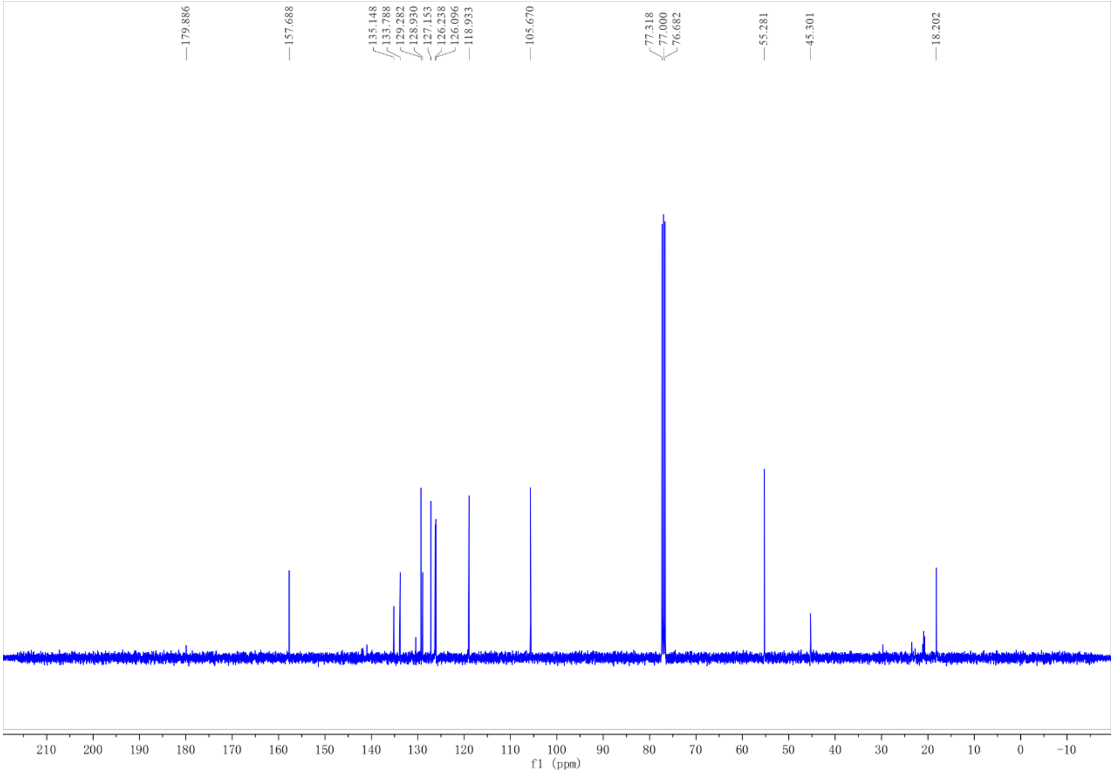

HPLC spectra:

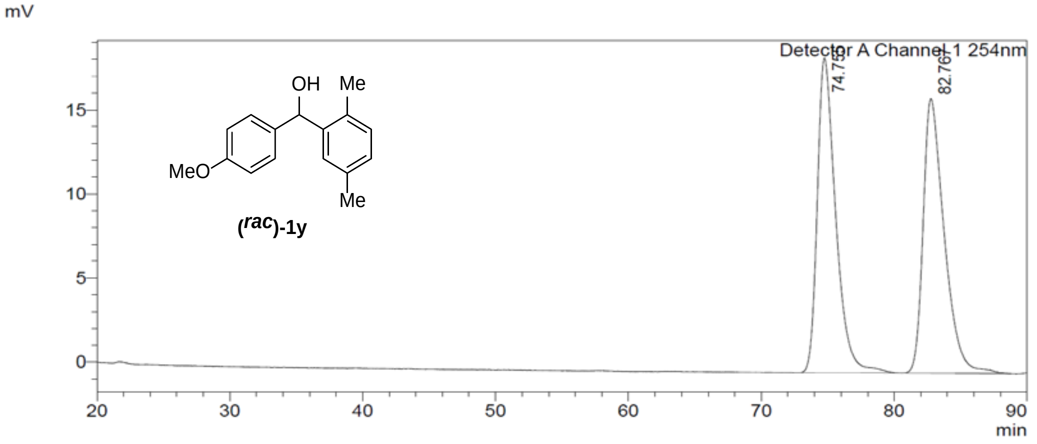

Peak#	Ret. Time	Area	Height	Peak Start	Area%	Width at 50% Height
1	74.755	1807604	18687	70.250	50.017	1.427
2	82.767	1806407	16312	80.508	49.983	1.644
Total		3614011	34999		100.000	

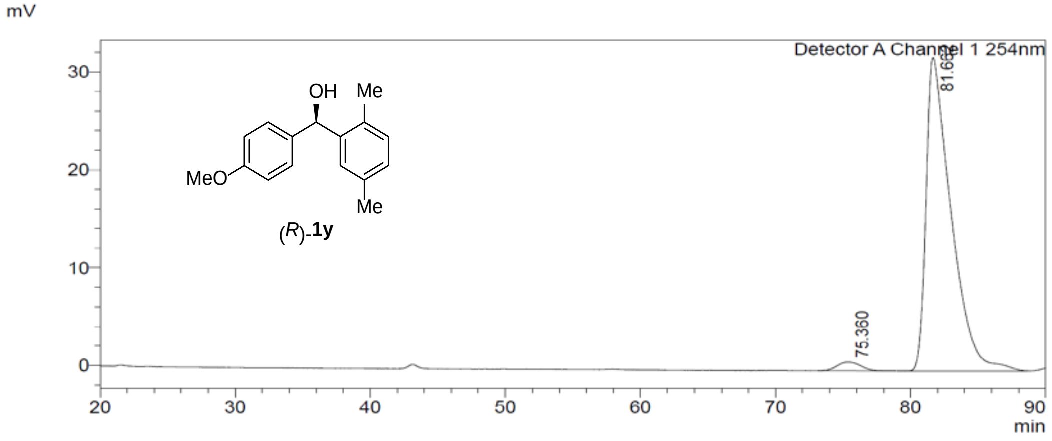

Peak#	Ret. Time	Area	Height	Peak Start	Area%	Width at 50% Height
1	75.360	105570	869	73.150	2.501	1.947
2	81.662	4115868	32019	78.992	97.499	1.905
Total		4221439	32888		100.000	

95% *ee* of (*R*)-**1y** was determined by HPLC: OJ-H Column, 5/95 *i*PrOH/hexane, 0.5 mL/min, 254 nm, 35 ^{o}C; retention time = 75.36 min (minor), 81.66 min (major).

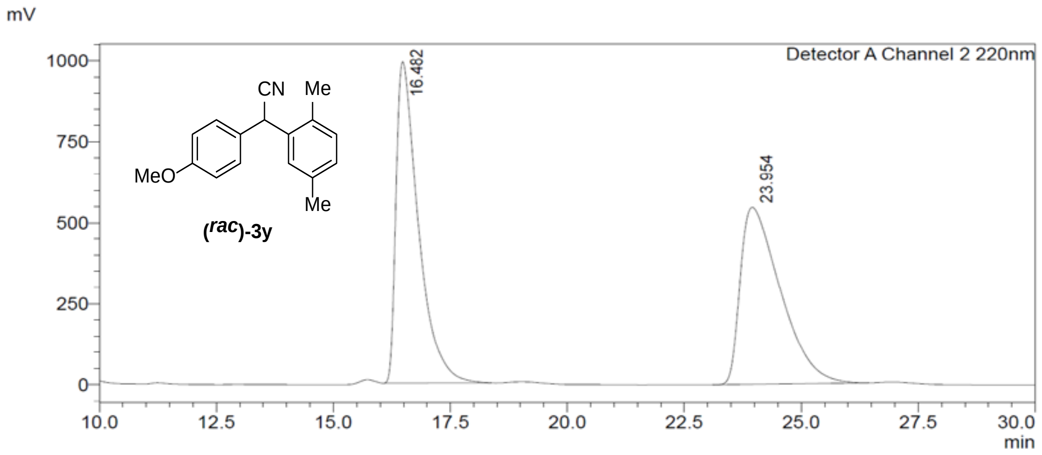

Peak#	Ret. Time	Area	Height	Peak Start	Area%	Width at 50% Height
1	16.482	32943578	991114	16.092	50.191	0.488
2	23.954	32692862	546447	23.117	49.809	0.925
Total		65636440	1537561		100.000	

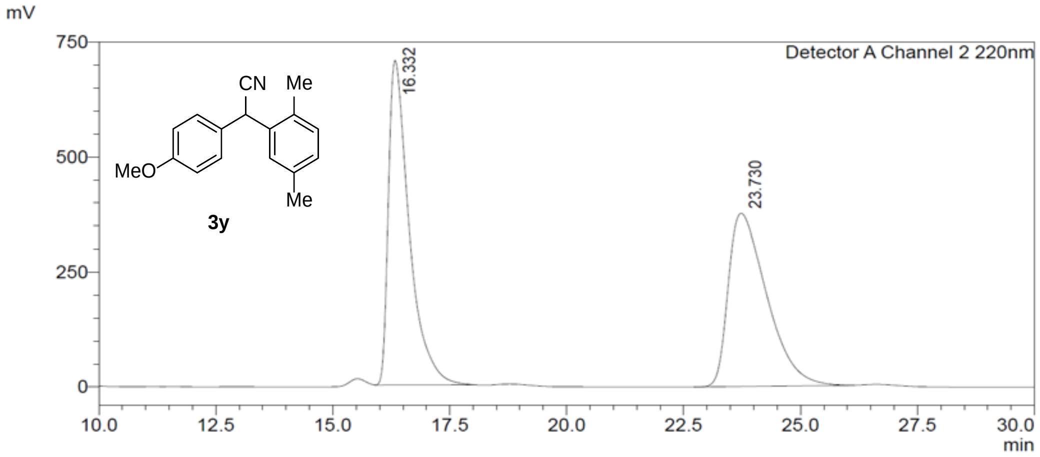

Peak#	Ret. Time	Area	Height	Peak Start	Area%	Width at 50% Height
1	16.332	21841649	706234	15.900	50.303	0.449
2	23.730	21578503	376470	22.725	49.697	0.881
Total		43420152	1082704		100.000	

0% *ee* of **3y** was determined by HPLC: AS-H Column, 5/95 *i*PrOH/hexane, 0.8 mL/min, 220 nm, 35 ^{o}C; retention time = 16.33 min (minor), 23.73 min (major)).

Other Pathways for the Direct Cyanation of Benzyl Alcohols：

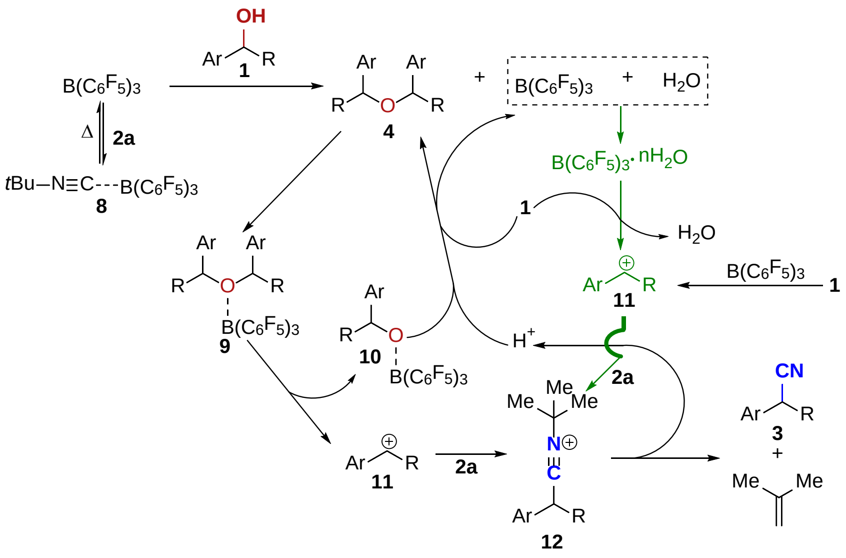

Scheme S1. Proposed mechanism.
